# Product reformulation in non-alcoholic beverages and foods after the implementation of front-of-pack warning labels in Mexico

**DOI:** 10.1371/journal.pmed.1004533

**Published:** 2025-03-18

**Authors:** Juan Carlos Salgado, Lilia S. Pedraza, Alejandra Contreras-Manzano, Tania C. Aburto, Lizbeth Tolentino-Mayo, Simon Barquera

**Affiliations:** 1 Centro de Investigación en Nutrición y Salud, Instituto Nacional de Salud Pública, Universidad No 655 Colonia Santa María, Cuernavaca, Morelos, Mexico; 2 Secretaría de Ciencia, Humanidades, Tecnología e Innovación, Mexico City, Mexico; University of Cambridge, UNITED KINGDOM OF GREAT BRITAIN AND NORTHERN IRELAND

## Abstract

**Background:**

In late March 2020, the Mexican government announced an updated norm to include front-of-pack warning labels for packaged foods and non-alcoholic beverages. Warning labels came into effect in October 2020. To avoid displaying warning labels, producers can reformulate their products by reducing the content of calories or critical nutrients targeted by the policy (added sugars, saturated fat, and sodium) or removing non-caloric sweeteners or added caffeine. The objective of this study is to assess changes in the percentage of products above warning-label cutoffs for calories and critical nutrients and changes in the content of calories and critical nutrients associated with warning labels in Mexico.

**Methods and findings:**

We used nutritional panel data collected by the Mexican National Institute of Public Health from ≈1,000 top-purchased products, which represented ≥60% of the market share for each of the included food groups according to household purchases in the Nielsen Consumer Panel commercial dataset for Mexico in 2016. Nutritional panel data is available for three periods: 2016−2017, T0 (pre-policy); Jul–Sep 2020, T1 (post-warning-label announcement); and Feb–Apr 2021, T2 (post-warning-label implementation). We assessed changes in T1 versus T0 (potential anticipatory reformulation before the warning-label implementation) and T2 versus T0 (reformulation after the warning-label implementation) by food group using generalized estimating equations for the percentage of products above warning-label cutoffs or containing non-caloric sweeteners or added caffeine, and fixed-effects linear models and quantile regressions for the content of calories and critical nutrients. Included food groups were cereal-based desserts, bread and other cereals, salty snacks, sweetened beverages, solid dairy, liquid dairy, instant food, and candies. At T0, the food group level with the lowest percentage of products with at least one calorie/nutrient content above warning-label cutoffs was instant food (77.8%); at T2, this fell to 52.6%. Based on our statistical models, we found that all food groups showed reductions in at least one type of warning label. The most common reductions in the percentage of products exceeding warning-label cutoffs were for sodium (up to −63.1 percentage points for bread and other cereals; 95% CI: −77.5, −48.6; *p*-value < 0.001), saturated fat (up to −26.3 percentage points for salty snacks; 95% CI: −35.8, −16.8; *p*-value < 0.001), and products containing non-caloric sweeteners (up to −29.0 percentage points for solid dairy; 95% CI: −40.7, −17.2; *p*-value < 0.001). The reductions in products above warning-label cutoffs were coupled with reductions in products’ content of calories and critical nutrients. According to quantile regressions, these reductions mostly occurred at the 50th–75th percentiles. Product reformulation mainly occurred in T2.

**Conclusion:**

Our findings show product reformulation due to reductions in critical nutrients/calories after the warning-label policy implementation, which entails improving the nutritional profile of the packaged food and beverage supply in Mexico.

## Author summary

**Why was this study done?**

- In October 2020, Mexico implemented warning labels, which were announced in March 2020, for packaged foods and non-alcoholic beverages as a mandatory food policy to control and prevent unhealthy diet-related non-communicable diseases.
- When a product’s energy or critical nutrient content exceeds policy-defined content cutoffs, that product must display the respective warning label. Moreover, products must also display a label when containing non-caloric sweeteners or added caffeine.
- Warning labels encourage product reformulation, as reductions in calories and policy-subject critical nutrients can lead to products displaying no or fewer warning labels.

**What did the researcher do and find?**

- We assembled a panel of the top-purchased packaged foods and non-alcoholic beverages in Mexico for three key periods: pre-policy, T0; post-warning-label announcement, T1; and post-warning-label implementation, T2.
- By comparing T1 versus T0 (potential anticipatory reformulation in advance of the policy implementation) and T2 versus T0 (post-policy reformulation), we aimed to assess product reformulation in terms of changes in calorie/critical nutrient content and the percentage of products exceeding warning-label cutoffs or containing non-caloric sweeteners or added caffeine.
- After the warning-label policy implementation, all analyzed food groups reported reductions in at least one type of warning label (the most common reductions were for the warning labels for “Excess sodium”, “Excess saturated fat”, and non-caloric sweeteners) coupled with reductions in products’ energy and critical nutrient contents. Some reductions by comparing T1 versus T0 suggest an anticipatory reformulation before the warning-label policy implementation; however, these reductions tended to be lower and less frequent than those in T2.
- Our findings are limited to top-purchased packaged foods and non-alcoholic beverages.
- The reduction in the use of non-caloric sweeteners after the warning-label policy implementation in Mexico contrasts with their increased use in Chile and Peru, whose warning-label systems do not target this kind of sweetener.
- Our reformulation findings point towards an improvement in the nutrient profile of packaged foods and non-alcoholic beverages after the implementation of the warning-label policy. Future studies should analyze the interplay of reformulation and warning-label use and their implications for population diet and health.

**What do these findings mean?**

- Our findings are limited to top-purchased packaged foods and non-alcoholic beverages.
- The reduction in the use of non-caloric sweeteners after the warning-label policy implementation in Mexico contrasts with their increased use in Chile and Peru, whose warning-label systems do not target this kind of sweetener.
- Our reformulation findings point towards an improvement in the nutrient profile of packaged foods and non-alcoholic beverages after the implementation of the warning-label policy. Future studies should analyze the interplay of reformulation and warning-label use and their implications for population diet and health.

## Abstract 3

Juan Carlos Salgado and colleagues assess changes in the content of calories and critical nutrients in packaged foods and non-alcoholic beverages in Mexico associated with the implementation of front-of-pack warning labels in 2020.

## Introduction

In Mexico, the overweight and obesity prevalence is close to 40% for children and teenagers and 75% for adults [1,2]. Meanwhile, 18% and 48% of Mexican adults live with diabetes or hypertension, respectively [3,4]. Costs related to overweight and obesity in Mexico represented 2% of the gross domestic product in 2019, and this figure is predicted to reach nearly 5% in 2060 [5]. Evidence shows that ultra-processed diets are a risk factor for obesity and related non-communicable chronic diseases [6,7]. In Mexico, the consumption of sugar-sweetened beverages and foods high in saturated fat and added sugar exceeds the energy contribution to the overall diet according to the recommendations from the Mexican dietary guidelines [8]. In light of this epidemic of unhealthy diet-related non-communicable diseases, in late March 2020, the government updated the Official Mexican Norm 051, which regulates the packages of non-alcoholic beverages and food. The updated norm includes front-of-pack warning labels and advertising package regulations [9]. All pre-packaged foods and non-alcoholic beverages are subject to the Official Mexican Norm 051, except breast milk substitutes, foods and beverages for infants and young children (under 2 years), oils and fats, sugar, and salt [9]. Products bought in bulk and prepared or packaged at the point of sale are not considered pre-packaged and are therefore excluded from Official Mexican Norm 051. Likewise, pre-packaged alcoholic beverages, cocktails, or mixers containing added sugars are excluded from the Official Mexican Norm 051 [9].

Front-of-pack labeling provides supplementary nutrition information on pre-packaged products to assist consumers in making healthier eating decisions by providing easier-to-understand information than the nutritional facts panel on the back of packages [10]. Specifically for warning labels, these consistently and easily inform on the excessive content of critical nutrients in non-essential food to discourage their consumption [11]. The updated Official Mexican Norm 051 indicates that packaged products with added sugar, sodium, and/or fat and critical nutrients/calories above specific cutoffs could display up to five black octagonal warning labels: “Excess calories”, “Excess sugars”, “Excess saturated fat”, “Excess *trans-*fat”, and “Excess sodium” [9]. Products containing added caffeine or non-caloric sweeteners must also present specific precautionary legends indicating that these substances are not recommended for children. The warning label system in Mexico differs from those in Chile and Peru, early warning-label policy adopters, by setting tighter nutrient-related cutoffs for warning labels and including the precautionary legends for added caffeine or non-caloric sweeteners [12]. In addition to the warning labels, the Official Mexican Norm 051 prohibits package advertising for children and nutrient-specific health/nutritional claims when products display warning labels [9]. While warning labels came into effect in October 2020, the implementation of the other package regulations was scheduled for April 2021. The government provided an additional 60-day extension to comply with all these regulations [13,14].

Evidence shows that food policies targeting the content of critical nutrients are effective in encouraging product reformulation. Such is the case of warning labels that encourage producers to reduce the content of policy-subject critical nutrients to decrease the number of warning labels displayed in their packages, thus mitigating the potential reduction in sales linked to the information conveyed by warning labels. Chile, the first country to implement a warning label system in 2016 for calories and a set of critical nutrients (sugar, saturated fat, and sodium), showed a meaningful and heterogeneous reformulation across food groups after the warning-label implementation. Overall, there was a seven percentage-point reduction from 51% to 44% of packaged food and beverages that exceeded at least one of the warning-label cutoffs [15]. The most common reductions, robust to different model specifications, were for the products above warning-label cutoffs for sugar (beverages, milks and milk-based drinks, breakfast cereals, and savory spreads) and sodium (savory spreads, cheeses, and sausages) [15]. Across these groups, most of them had reductions ≥50% of products whose sugar or sodium content was below the relevant warning-label cutoffs after the policy implementation [15]. Meanwhile, reductions in products above the calorie warning-label cutoff were specific for breakfast cereals and savory spreads, and reductions in products above the saturated fat warning-label cutoff were limited to savory spreads [15]. Regarding the content of calories and critical nutrients, reformulation generally occurred when the relevant warning-label cutoff was below the 75th of the content distribution [15]. Moreover, the product’s content of critical nutrients tended to bunch around the warning-label cutoffs [15,16]. It is worth noting that no major anticipatory reformulation occurred before the warning label policy was in place in Chile [17]. In Peru, after the warning-label implementation, there was a reduction in sugar in ultra-processed beverages and a reduction of saturated and *trans*-fats for ultra-processed food, coupled with a reduction between 20 and 28 percentage points of products subject to displaying any warning labels [18]. Evidence shows that voluntary front-of-pack systems, such as the Health Star Rating, are also associated with food reformulation [19]. However, mandatory food policies, compared to voluntary food policies, are expected to lead to a greater extent of product reformulation [20]. Other nutrient-profile policies, such as taxes on sugar content for sugar-sweetened beverages in South Africa and the United Kingdom, are linked to products’ sugar reductions [21–23]. For the case of the United Kingdom, evidence shows early reformulation in anticipation of the sugar-density tax implementation [24,25]. In the context of the warning label policy in Mexico, leading packaged food and beverage firms have reported an average reformulation of their product portfolio of around 50%, with a firm reaching up to 80% [26].

Our study aims to assess the gradual product reformulation associated with the warning label policy in Mexico in terms of changes in the percentage of products above warning-label cutoffs and the content of calories and critical nutrients for a set of packaged food and non-alcoholic beverages. Our study’s findings are relevant in three regards. First, we provide evidence of an immediate potential result of the warning label policy in Mexico, i.e., a change in the nutritional profile in the supply of packaged food and beverages. Second, product reformulation can inform future warning-label evaluations assessing changes in food purchases and related critical nutrients in Mexico. Third, because Mexico is the first country to include warning labels for non-caloric sweeteners and caffeine, our results are relevant for countries including similar warning labels (i.e., Argentina and Colombia) or countries designing new warning-label systems.

## Methods

### Data sources

Our primary information source is the NutrINSPector system developed by the Mexican National Institute of Public Health, which consists of a mobile app and a web platform. The app captures photographs of all sides of food and beverage packages, which are then synchronized and uploaded to the platform. Nutritional information, price, ingredients list, health claims, promotions, and other label details are recorded following the INFORMAS food labeling protocol [27]. The product sample in NutrINSPector was selected using the Nielsen Consumer Panel commercial dataset for Mexico in 2016, which contains information on household purchases of packaged food and beverages [28]. Products contributing at least 60% of the total volume and sales within each food category were included, while categories irrelevant to the warning-label evaluation (e.g., those not subject to regulation or lacking critical nutrients) were excluded. The NutrINSPector information in this study includes two data collection waves. The first wave collected information from 1,003 products after the warning-label policy announcement, but before its implementation (i.e., Jul–Sep 2020). For this data collection, trained fieldworkers visited major and minor retailers in Mexico City and Cuernavaca. Due to COVID-19 restrictions, products were bought and subsequently photographed, coded, and entered into the NutrINSPector system. The second wave was collected after the warning-label implementation (i.e., Feb–Apr 2021) with an expanded sample of 1,126 products, which intended to include the ones collected in the first wave to achieve maximum comparability. In the second NutrINSPector wave, products were photographed on-site, without purchasing, using the same data entry and cleaning methods. It is worth noting that products in the second NutrINSPector wave keep representing at least 60% of their food groups’ market share according to household purchases in 2021 (based on Kantar WorldPanel Mexico, 2021).

In the context of this study, we defined a product as a unique combination of brand, flavor, and regular/light version. We merged products in NutrINSPector with their counterparts in a product dataset collected by the Mexican National Institute of Public Health in 2016−17 as part of the INFORMAS study [12,29], prioritizing information from 2017 when available except for dry products such as powder drink mixes, coffee and tea, chocolate powder-based milk, and instant food (e.g., soups, noodles, chicken broth powder) whose 2017 nutritional information was not reconstituted (or in an as consumed form). To merge the 2016−2017 dataset and NutrINSpector, we first used the products’ universal product code, and when it was not possible, we merged according to information based on our product definition. When more than one product in the 2016−2017 dataset met the latter merging criterion, we chose one product randomly. For products in both databases, 62% were merged based on universal product code and 38% based on our product definition. When a product was unavailable in the 2016−2017 dataset, we looked for it in Open Food Facts, a crowdsourced website with pictures of packaged products and nutritional information [30,31]. We only retrieved information from Open Food Facts when product packages (1) displayed the guideline-daily-amount labeling system, which preceded the warning label system in Mexico, thus ensuring that the nutritional information predated the warning-label implementation or (2) displayed no warning labels for products whose information was collected in countries other than Mexico [30,31]. Thus, we have information from three key periods: (1) data from food labels in 2016−2017 and Open Food Facts, which preceded for 3 years the warning-label announcement, (2) the first data collection in NutrINSPector, which was between the warning-label announcement (i.e., March 2020) and implementation (i.e., October 2020), and (3) the second data collection in NutrINSPector after the warning-label implementation (i.e., after October 2020). Hereafter, we will refer to these three key periods as T0 for the 2016−2017 data and Open Food Facts, and T1 and T2 for the first and second NutrINSPector data collections, respectively.

### Analytical data

**Fig 1:**
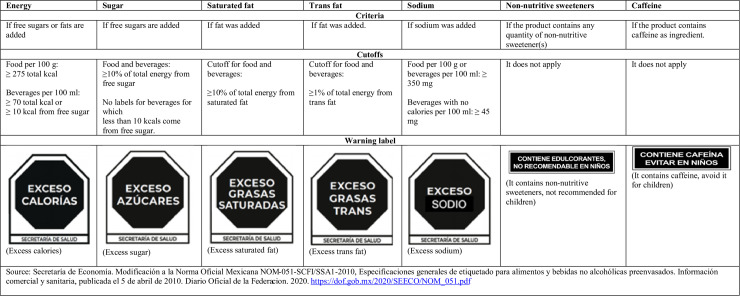
Criteria and cutoffs by critical nutrient for the first stage of the implementation (October 2020–September 2023) of the front-of-pack warning label policy in Mexico.

We briefly describe the main steps to set the above data into our analytical data and provide more details in File A in S1 Text. Before the warning-label implementation, reporting the content of added sugars and *trans-*fat was optional, making it impossible to assess their content from the label [32]. Therefore, we approximated added sugars for T0–T2 following the procedure by the Pan American Health Organization designed to estimate added sugars based on ingredients and other available information from the label, such as total sugars and product composition [33]. Meanwhile, we discarded using *trans-*fat information because this information was missing or equal to zero for most products. To determine the respective warning labels in T0–T2, we reconstituted not-ready-to-consume products (e.g., drink mix or instant soups) to their as-consumed form according to their preparation instructions, homologated products to grams (milliliters) when they were part of a food (beverage) group, and standardized content of calories and critical nutrients per 100 g-ml. For potential outliers for nutritional information and differences in reconstituted not-ready-to-consume products or units of measure, we corroborated or corrected information in the content of calories and critical nutrients. Subsequently, we identified products above each warning-label cutoff for calories and critical nutrients in each wave (i.e., T0, T1, and T2) following the nutrient profile for the first regulation phase (October 2020–September 2023) as in Fig 1. Meanwhile, we identified products containing non-caloric sweeteners or added caffeine based on the product’s ingredients list.

After dropping products not affected by the policy (no added sugar/sodium/fat/non-caloric sweeteners/caffeine for all periods with available information or not subject to the policy, such as infant formula), had missing information, or had information only in T1 or T2, our final analytic sample is an unbalanced panel with 1,051 products that included 994 observations in T0, 859 observations in T1, and 973 observations in T2.

We classify products in our analytical data into eight food groups that we considered relevant due to their common consumption by the Mexican population and their significant contribution to their intake of the critical nutrients targeted by the warning labels. These eight food groups are cereal-based desserts, bread and other cereals, salty snacks, sweetened beverages, liquid dairy, solid dairy, instant food, and candies. Groups are in grams except for sweetened beverages and liquid dairy in milliliters.

See Table A in S1 Text for more information on dropped observations in NutrINSPector, Table B in S1 Text for the analytical data size by food group, wave, and information source (i.e., INFORMAS, Open Food Fact, or NutrINSPector), and Table C in S1 Text for the subcategories conforming to included eight food groups and their contribution to the food group sample size. For Table B in S1 Text, it is worth noting that Open Food Fact products tend to represent less than 10% of observations in T0, with the lowest and highest figures equivalent to 4% and 23% for instant food and bread and other cereals, respectively. Meanwhile, Open Food Fact products from countries other than Mexico tend to account for less than 1% of observations in T0.

### Study design

Based on a before-and-after study design, we assess product reformulation over time in terms of (1) changes in the percentages of products above specific warning-label cutoffs for calories and critical nutrients or containing non-caloric sweeteners or added caffeine and (2) the changes in calories and critical nutrient content by comparing T1 versus T0 and T2 versus T0. The comparison of T1 versus T0 is intended to measure potential anticipatory reformulation once the warning label policy was announced in March 2020, but it was not yet in place, while T2 versus T0 corresponds to post-policy reformulation. In practice, these comparisons will enter the statistical models, which we explain in detail below, as a dummy variable for T1 and another dummy variable for T2, equivalent to our independent variables of interest. Meanwhile, the dependent variable will be (1) the set of binary variables to identify whether a product exceeds a specific warning-label cutoff for calories or critical nutrients or contains non-caloric sweeteners or added caffeine and (2) the set of continuous variables for calories and critical nutrient content. It is worth noting that we will run an individual model by food group and either by kind of warning label or calorie content and kind of critical nutrient.

### Statistical analyses

For descriptive purposes, we report the percentage of products above the specific warning-label cutoff for calories and critical nutrients or containing non-caloric sweeteners or added caffeine. Moreover, we calculate the percentile of the calorie/nutrient distribution at which the respective warning-label cutoff falls. Finally, we calculate Kernel densities at the calorie/critical nutrient level. We report all descriptive information by wave and food group.

For our statistical models, we select model specifications that allow us to account for the panel structure of our analytical data and thus accommodate the expected correlation arising at the product level from nutritional information for the same product in more than one period. Thus, we select generalized estimating equations to estimate the changes in the percentages of products above warning-label cutoffs for calories and critical nutrients or containing non-caloric sweeteners or added caffeine. For generalized estimating equations, we set a binomial family with a logit link function, exchangeable within-unit-of-analysis correlation, and robust standard errors. The selected family and link function will allow us to account for the binary nature of the dependent variable (e.g., whether a product exceeds or does not exceed a specific warning-label cutoff). We report as marginal effects the changes in percentages of products above warning-label cutoffs or containing non-caloric sweeteners or added caffeine in T1 versus T0 and T2 versus T0. It is worth highlighting that generalized estimating equation models do not report percentage-based results; instead, they present them as proportions. However, to convey information more directly and clearly, we scaled results from these models by a factor of 100 to go from proportions to percentages. We run no generalized estimating equations models (1) when the proportion for a period was zero or one because it resulted in perfect multi-collinearity between the proportion and the period nor (2) when few products had a change for the warning label of interest over time due to a lack of variation to identified models’ coefficients.

To estimate the average change in calories and critical nutrient content, we select linear regression models with product-fixed effects and cluster standard errors at the product level. Due to warning-label cutoffs being fixed across all food groups (with minor adjustments depending on food or beverages; see more details in Fig 1) and calorie and critical nutrient contents likely to vary across food groups, we could expect differential extents of changes over the distributions of calorie and critical nutrient contents by food group to avoid displaying warning labels. Thus, we complement the fixed effects models described above (focusing on average changes) with unconditional quantile regressions with product fixed effects to analyze changes in the distribution of the content of calories and critical nutrients, for which we focused on the 25th, 50th, and 75th percentiles. For these regressions, we ran 3 two-stage models (each for 25th, 50th, and 75th quantiles) by food group and critical nutrient/calorie content because we first needed to calculate the recentered influence function for the relevant quantile, which depends on the cumulative and density functions of the distribution of the dependent variable. Then, we used this estimate as the outcome variable in a linear model with product-fixed effects [34]. Due to this two-stage nature, we bootstrap standard errors at the product level with 500 repetitions based on the Stata command *xtrifreg* [34]. Moreover, and consistent with the linear fixed-effect models for average changes, we set clustered standard errors at the product level for quantile regressions.

Across all model specifications, we set a critical *p*-value of 0.05 to define statistical significance changes. All models described above account for the correlation arising from information for the same product (i.e., the unit of analysis) in more than one period by setting clustering standard errors at the product level. We conducted all statistical analyses in Stata 18 [35]. As supporting information, we provide the data (S1 Data) and codes (S1 Code for binary outcomes and S2 Code for continuous outcomes) on which analyses are based.

## Results

Table 1 shows the summary statistics of the percentage of products above specific warning-label cutoffs or containing non-caloric sweeteners or added caffeine by wave and food group. We observed a reduction in the display of “Any warning label” across most food groups between T0 and T2, with the largest reductions in bread and other cereals (from 100% in T0 to 61.4% in T2) and instant food (from 77.8% in T0 to 52.6% in T2). Even though the reductions by the type of warning label were heterogenous, reductions in products above the warning-label cutoffs for “Excess sodium” (e.g., cereal-based desserts, bread and other cereals, and instant food), “Excess saturated fat” (e.g., salty snacks and liquid and solid dairy), and non-caloric sweeteners (e.g., cereal-based desserts and liquid and solid dairy) from T0 and T2 are worth noting. For salty snacks, we observed 12.4% of products containing non-caloric sweeteners in T1, which dropped to zero in T2. Moreover, no dairy products were above the warning-label cutoff for “Excess sodium”. Finally, we saw no meaningful change in added caffeine use for sweetened beverages, the only group with this ingredient in our analytic data. Changes between T1 and T0 tend to be not as large as in the case of T2 and T0.

**Table 1:** Summary statistics of the percentage of products above warning-label cutoffs for calories and critical nutrients.

Outcome	T0	T1	T2
	Percentage	Percentage	Percentage
Cereal-based desserts	*n* = 228 obs	*n* = 221 obs	*n* = 221 obs
Any warning label	99.1 (9.3)	99.5 (6.7)	97.3 (16.3)
Excess calories (Cutoff at T0: 6th percentile)	94.3 (23.2)	94.1 (23.6)	90.5 (29.4)
Excess sugar (Cutoff at T0: 8th percentile)	92.1 (27.0)	92.8 (26.0)	88.7 (31.7)
Excess saturated fat (Cutoff at T0: 46th percentile)	53.9 (50.0)	56.1 (49.7)	40.7 (49.2)
Excess sodium (Cutoff at T0: 52th percentile)	48.2 (50.1)	41.6 (49.4)	19.0 (39.3)
Non-caloric sweeteners	24.6 (43.1)	24.4 (43.1)	13.6 (34.3)
Caffeine	0.0 (0.0)	0.0 (0.0)	0.0 (0.0)
**Bread and other cereals**	***n* = 43 obs**	***n* = 44 obs**	***n* = 44 obs**
Any warning label	100.0 (0.0)	81.8 (39.0)	61.4 (49.3)
Excess calories (Cutoff at T0: 51th percentile)	48.8 (50.6)	34.1 (47.9)	27.3 (45.1)
Excess sugar (Cutoff at T0: 51th percentile)	48.8 (50.6)	36.4 (48.7)	43.2 (50.1)
Excess saturated fat (Cutoff at T0: 91th percentile)	9.3 (29.4)	4.5 (21.1)	4.5 (21.1)
Excess sodium (Cutoff at T0: 7th percentile)	93.0 (25.8)	70.5 (46.2)	29.5 (46.2)
Non-caloric sweeteners	4.7 (21.3)	4.5 (21.1)	2.3 (15.1)
Caffeine	0.0 (0.0)	0.0 (0.0)	0.0 (0.0)
**Salty snacks**	***n* = 135 obs**	***n* = 105 obs**	***n* = 128 obs**
Any warning label	96.3 (19.0)	95.2 (21.4)	91.4 (28.1)
Excess calories (Cutoff at T0: 1th percentile)	85.2 (35.7)	81.0 (39.5)	83.6 (37.2)
Excess sugar (Cutoff at T0: 94th percentile)	5.9 (23.7)	8.6 (28.1)	6.3 (24.3)
Excess saturated fat (Cutoff at T0: 39th percentile)	61.5 (48.8)	46.7 (50.1)	34.4 (47.7)
Excess sodium (Cutoff at T0: 13th percentile)	87.4 (33.3)	83.8 (37.0)	74.2 (43.9)
Non-caloric sweeteners	0.7 (8.6)	12.4 (33.1)	0.0 (0.0)
Caffeine	0.0 (0.0)	0.0 (0.0)	0.0 (0.0)
**Sweetened beverages**	***n* = 176 obs**	***n* = 143 obs**	***n* = 170 obs**
Any warning label	96.0 (19.6)	97.9 (14.4)	92.9 (25.7)
Excess calories (Cutoff at T0: 99th percentile)^a^	64.2 (48.1)	56.6 (49.7)	52.4 (50.1)
Excess sugar (Cutoff at T0: 28th percentile)^a^	64.2 (48.1)	56.6 (49.7)	52.4 (50.1)
Excess saturated fat (Cutoff at T0: 97th percentile)	2.8 (16.7)	0.7 (8.4)	2.9 (16.9)
Excess sodium (Cutoff at T0: 99th percentile)^a^	1.1 (10.6)	2.1 (14.4)	0.0 (0.0)
Non-caloric sweeteners	62.5 (48.6)	71.3 (45.4)	66.5 (47.3)
Caffeine	9.1 (28.8)	7.0 (25.6)	10.0 (30.1)
**Liquid dairy**	***n* = 115 obs**	***n* = 86 obs**	***n* = 119 obs**
Any warning label	88.7 (31.8)	94.2 (23.5)	85.7 (35.1)
Excess calories (Cutoff at T0: 63th percentile)^a^	74.8 (43.6)	77.9 (41.7)	69.7 (46.1)
Excess sugar (Cutoff at T0: 20th percentile)^a^	74.8 (43.6)	77.9 (41.7)	69.7 (46.1)
Excess saturated fat (Cutoff at T0: 80th percentile)	20.0 (40.2)	23.3 (42.5)	10.1 (30.2)
Excess sodium (Cutoff at T0: 99th percentile)^a^	0.0 (0.0)	0.0 (0.0)	0.0 (0.0)
Non-caloric sweeteners	34.8 (47.8)	40.7 (49.4)	21.8 (41.5)
Caffeine	0.0 (0.0)	0.0 (0.0)	0.0 (0.0)
**Solid dairy**	***n* = 66 obs**	***n* = 62 obs**	***n* = 69 obs**
Any warning label	98.5 (12.3)	75.8 (43.2)	85.5 (35.5)
Excess calories (Cutoff at T0: 94th percentile)	4.5 (21.0)	0.0 (0.0)	0.0 (0.0)
Excess sugar (Cutoff at T0: 30th percentile)	69.7 (46.3)	62.9 (48.7)	69.6 (46.4)
Excess saturated fat (Cutoff at T0: 52th percentile)	48.5 (50.4)	14.5 (35.5)	27.5 (45.0)
Excess sodium (Cutoff at T0: 99th percentile)	0.0 (0.0)	0.0 (0.0)	0.0 (0.0)
Non-caloric sweeteners	40.9 (49.5)	30.6 (46.5)	13.0 (33.9)
Caffeine	0.0 (0.0)	0.0 (0.0)	0.0 (0.0)
**Instant food**	***n* = 99 obs**	***n* = 89 obs**	***n* = 97 obs**
Any warning label	77.8 (41.8)	77.5 (42.0)	52.6 (50.2)
Excess calories (Cutoff at T0: 98th percentile)	2.0 (14.1)	0.0 (0.0)	1.0 (10.2)
Excess sugar (Cutoff at T0: 78th percentile)	22.2 (41.8)	24.7 (43.4)	16.5 (37.3)
Excess saturated fat (Cutoff at T0: 75th percentile)	25.3 (43.7)	22.5 (42.0)	17.5 (38.2)
Excess sodium (Cutoff at T0: 34th percentile)	65.7 (47.7)	58.4 (49.6)	38.1 (48.8)
Non-caloric sweeteners	0.0 (0.0)	0.0 (0.0)	0.0 (0.0)
Caffeine	0.0 (0.0)	0.0 (0.0)	0.0 (0.0)
**Candies**	***n* = 132 obs**	***n* = 109 obs**	***n* = 125 obs**
Any warning label	100.0 (0.0)	100.0 (0.0)	99.2 (8.9)
Excess calories (Cutoff at T0: 46th percentile)	53.8 (50.0)	52.3 (50.2)	52.8 (50.1)
Excess sugar (Cutoff at T0: 11th percentile)	88.6 (31.9)	92.7 (26.2)	89.6 (30.6)
Excess saturated fat (Cutoff at T0: 45th percentile)	54.5 (50.0)	60.6 (49.1)	56.8 (49.7)
Excess sodium (Cutoff at T0: 89th percentile)	10.6 (30.9)	9.2 (29.0)	12.0 (32.6)
Non-caloric sweeteners	15.9 (36.7)	13.8 (34.6)	8.8 (28.4)
Caffeine	0.0 (0.0)	0.0 (0.0)	0.0 (0.0)

Note: Standard deviations are in parentheses. Summary statistics are rounded to one decimal place. “Any warning label” equals one when the product displays at least one warning label for calories, sugar, saturated fat, sodium, non-caloric sweeteners, or caffeine.

^a^Beverages are also subject to the following criteria: when ≥10 calories come from free sugar, the product has to display the warning label of “excess calories”; when less than 10 calories come from free sugar, the product is exempted from displaying the warning label of “excess sugar”; when ≥45 mg sodium/100 ml and the product has no calories, the product has to display the warning label of “excess sodium”.

Table 1 also shows the percentiles of the calorie/nutrient distribution in T0 where the specific warning-label cutoff falls at the food group level. Generally, this cutoff is close to or above 50th percentile. Table E in S1 Text shows equivalent cutoff-related percentiles for all waves (i.e., T0, T1, and T2). In this table, we can see that these percentiles, in general, inversely mirror the changes in the percentage of products above warning-label cutoffs, i.e., reductions in these percentages generally entail a shift in the percentiles towards the right. Figs A–H in S1 Text show the Kernel densities of critical nutrients/calorie content. Based on these figures, we observed, in general, over time, a distribution shift of the relevant critical nutrient towards the left. For some food groups, we see a bunching of the critical nutrient in T2 right below the relevant warning-label cutoff (i.e., sodium for cereal-based desserts, bread and other cereals, and instant food; calories for bread and other cereals; and saturated fat for salty snacks).

Based on generalized estimating equations, column one in Table 2 shows the estimated percentage of products above warning-label cutoffs for calories and critical nutrients by food group in T0, and columns two and three present the change in percentage points in T1 and T2 compared to T0, respectively. It is worth noting that while similar information is shown in Tables 1 and 2, this information can slightly vary in magnitude because percentages in the latter table are model-based estimates, which in turn allowed us to test whether changes over time were statistically significant.

**Table 2:** Change in the percentage of products above warning-label cutoffs.

Outcome	(1)	(2)	(3)
	T0	T1 vs. T0	T2 vs. T0
	Estimated percentage	Change in percentage points^a^	Change in percentage points^a^
**Cereal based desserts (*n* = 670 obs)**			
Any warning label	98.9	0.6 [0.357]	−1.6 [0.124]
	(97.6,100.2)	(−0.6,1.8)	(−3.6,0.4)
Excess calories (Cutoff at T0: 6th percentile)	93.3	1.0 [.161]	−2.3 [0.090]
	(90.2,96.5)	(−0.4,2.3)	(−4.9,0.4)
Excess sugar (Cutoff at T0: 8th percentile)	92.0	0.1 [0.837]	**−3.0 [0.021]**
	(88.6,95.4)	(−1.2,1.5)	**(−5.6,−0.5)**
Excess saturated fat (Cutoff at T0: 46th percentile)	54.0	1.9 [0.119]	**−12.5 [<0.001]**
	(47.7,60.3)	(−0.5,4.2)	**(−17.3,−7.7)**
Excess sodium (Cutoff at T0: 52th percentile)	47.5	**−5.7 [0.013]**	**−28.7 [<0.001]**
	(41.1,53.9)	**(−10.2,-1.2)**	**(−35.0,-22.3)**
Non-caloric sweeteners	24.5	0.0 [0.972]	**−11.5 [<0.001]**
	(19.1,30.0)	(−2.7,2.8)	**(−16.4,−6.6)**
**Bread and other cereals (*n* = 131 obs)**			
Any warning label^b^	---	---	---
Excess calories (Cutoff at T0: 51th percentile)	54.0	**−21.1 [<0.001]**	**−27.9 [<0.001]**
	(38.6,69.5)	**(−33.5,−8.6)**	**(−41.9,−14.0)**
Excess sugar (Cutoff at T0: 51th percentile)	50.0	**−13.3 [0.045]**	−10.2 [0.088]
	(35.3,64.7)	**(−26.3,−0.3)**	(−21.8,1.5)
Excess saturated fat (Cutoff at T0: 91th percentile)	11.6	−7.5 [0.065]	−7.5 [0.065]
	(1.7,21.5)	(−15.4,0.5)	(−15.4,0.5)
Excess sodium (Cutoff at T0: 7th percentile)	93.0	**−21.8 [<0.001]**	**−63.1 [<0.001]**
	(85.3,100.7)	**(−34.1,−9.5)**	**(−77.5,−48.6)**
Non-caloric sweeteners	4.5	0.4 [0.934]	−2.0 [0.626]
	(−1.9,10.8)	(−8.5,9.2)	(−10.1,6.1)
**Salty snacks (*n* = 368 obs)**			
Any warning label	96.1	0.2 [0.908]	**−5.0 [0.043]**
	(92.8,99.3)	(−3.7,4.2)	**(−9.8,−0.2)**
Excess calories (Cutoff at T0: 1th percentile)	83.9	−0.1 [0.941]	−0.9 [0.622]
	(77.8,90.0)	(−3.2,3.0)	(−4.3,2.6)
Excess sugar (Cutoff at T0: 94th percentile)	7.0	0.7 [0.516]	−1.5 [0.190]
	(2.8,11.3)	(−1.4,2.8)	(−3.6,0.7)
Excess saturated fat (Cutoff at T0: 39th percentile)	60.5	**−9.4 [0.016]**	**−26.3 [<0.001]**
	(52.4,68.7)	**(−17.1,−1.7)**	**(−35.8,-16.8)**
Excess sodium (Cutoff at T0: 13th percentile)	86.7	−1.5 [0.571]	**−13.2 [<0.001]**
	(81.0,92.3)	(−6.6,3.6)	**(−20.2,−6.1)**
Non-caloric sweeteners^b^	---	---	---
**Sweetened beverages (*n* = 489 obs)**			
Any warning label	96.0	0.6 [0.555]	**−3.1 [0.044]**
	(93.1,98.8)	(−1.3,2.5)	**(−6.1,−0.1)**
Excess calories (Cutoff at T0: 99th percentile)^c^	63.6	**−7.7 [<0.001]**	**−10.7 [<0.001]**
	(56.6,70.6)	**(−12.2,−3.1)**	**(−15.9,−5.6)**
Excess sugar (Cutoff at T0: 28th percentile)^c^	63.6	**−7.7 [<0.001]**	**−10.7 [<0.001]**
	(56.6,70.6)	**(−12.2,−3.1)**	**(−15.9,−5.6)**
Excess saturated fat (Cutoff at T0: 97th percentile)	2.8	−0.4 [0.625]	0.3 [0.258]
	(0.4,5.2)	(−2.1,1.2)	(−0.2,0.7)
Excess sodium^b^^,^^c^	---	---	---
Non-caloric sweeteners	62.3	**7.2 [0.004]**	2.5 [0.388]
	(55.2,69.4)	**(2.3,12.1)**	(−3.2,8.2)
Caffeine	8.9	−1.3 [0.391]	0.6 [0.689]
	(4.7,13.1)	(−4.3,1.7)	(−2.4,3.7)
**Liquid dairy (*n* = 320 obs)**			
Any warning label	88.8	**3.8 [0.009]**	−3.5 [0.088]
	(83.3,94.4)	**(0.9,6.7)**	(−7.6,0.5)
Excess calories (Cutoff at T0: 63th percentile)^c^	74.3	−1.0 [0.649]	−4.9 [0.066]
	(66.6,82.1)	(−5.2,3.2)	(−10.1,0.3)
Excess sugar (Cutoff at T0: 20th percentile)^c^	74.3	−1.0 [0.649]	−4.9 [0.066]
	(66.6,82.1)	(−5.2,3.2)	(−10.1,0.3)
Excess saturated fat (Cutoff at T0: 80th percentile)	20.2	2.2 [0.572]	**−9.8 [0.001]**
	(13.0,27.4)	(−5.3,9.7)	**(−15.8,−3.8)**
Excess sodium^b^^,^^c^	---	---	---
Non-caloric sweeteners	35.3	6.1 [0.131]	**−14.5 [0.003]**
	(26.7,43.9)	(−1.8,14.0)	**(−24.0,−5.0)**
**Solid dairy (*n* = 197 obs)**			
Any warning label	98.6	**−22.0 [<0.001]**	**−13.2 [0.004]**
	(95.6,101.6)	**(−32.2,−11.7)**	**(−22.3,−4.1)**
Excess calories^b^	---	---	---
Excess sugar (Cutoff at T0: 30th percentile)	69.8	−2.4 [0.186]	−0.8 [0.636]
	(59.1,80.5)	(−6.0,1.2)	(−4.1,2.5)
Excess saturated fat (Cutoff at T0: 52th percentile)	48.9	**−34.0 [<0.001]**	**−21.4 [0.004]**
	(36.7,61.0)	**(−47.6,−20.3)**	**(−35.9,−6.9)**
Excess sodium^b^	---	---	---
Non-caloric sweeteners	42.2	**−12.1 [0.012]**	**−29.0 [<0.001]**
	(30.2,54.2)	**(−21.6,−2.7)**	**(−40.7,−17.2)**
**Instant food (*n* = 285 obs)**			
Any warning label	77.5	−0.3 [0.929]	**−24.3 [<0.001]**
	(69.3,85.7)	(−6.7,6.1)	**(−34.2,−14.5)**
Excess calories^b^	---	---	---
Excess sugar (Cutoff at T0: 78th percentile)	23.0	0.0 [0.976]	−5.0 [0.093]
	(14.8,31.2)	(−2.5,2.5)	(−10.8,0.8)
Excess saturated fat (Cutoff at T0: 75th percentile)	24.7	0.9 [0.699]	−5.8 [0.091]
	(16.2,33.1)	(−3.7,5.5)	(−12.5,0.9)
Excess sodium (Cutoff at T0: 34th percentile)	65.7	−7.1 [0.091]	**−26.5 [<0.001]**
	(56.3,75.0)	(−15.3,1.1)	**(−36.5,−16.4)**
Non-caloric sweeteners^b^	---	---	---
**Candies (*n* = 366 obs)**			
Any warning label^b^	---	---	---
Excess calories (Cutoff at T0: 46th percentile)	53.3	−1.8 [0.136]	−0.8 [0.580]
	(45.0,61.6)	(−4.2,0.6)	(−3.6,2.0)
Excess sugar (Cutoff at T0: 11th percentile)	89.3	0.3 [0.558]	0.1 [0.961]
	(84.2,94.5)	(−0.8,1.5)	(−2.2,2.3)
Excess saturated fat (Cutoff at T0: 45th percentile)	54.6	−0.3 [0.897]	2.1 [0.324]
	(46.3,62.8)	(−4.8,4.2)	(−2.1,6.3)
Excess sodium^b^	---	---	---
Non-caloric sweeteners	16.1	−0.4 [0.843]	**−6.6 [0.008]**
	(10.0,22.3)	(−4.8,3.9)	**(−11.5,−1.7)**

Note: *P*-values in square brackets. Ninety-five percent confidence intervals in parentheses. *P*-values are rounded to three decimal places. Coefficients and confidence intervals are rounded to one decimal place. Statistically significant estimates when *p*-value < 0.05 are bolded. “Any warning label” equals one when the product displays at least one warning label for calories, sugar, saturated fat, sodium, non-caloric sweeteners, or caffeine.

^a^Changes in proportions are calculated as marginal effects.

^b^We did not run a model for this label for multi-collinearity problems between the proportion (either zero or one) and the period or due to the lack of variation for the label within products.

^c^Beverages are also subject to the following criteria: when ≥10 calories come from free sugar, the product has to display the warning label of “excess calories”; when less than 10 calories come from free sugar, the product is exempted from displaying the warning label of “excess sugar”; when ≥45 mg sodium/100 ml and the product has no calories, the product has to display the warning label of “excess sodium”.

Overall, in Table 2, we see that there were reductions in the percentage of products that would be subject to “Any warning label” in T2, ranging between 3.1 percentage points for sweetened beverages (95% CI: −6.1, −0.1; *p*-value = 0.044) and 24.3 percentagepoints for instant food (95% CI: −34.2, −14.5; *p*-value < 0.001). The most common statistically significant reductions in T2 compared to T0 were for products above the warning-label cutoffs for “Excess sodium”, “Excess saturated fat”, and containing non-caloric sweeteners. Reductions in T2 for “Excess sodium” include cereal-based desserts (−28.7 percentage points; 95% CI: −35, −22.3; *p*-value < 0.001), bread and other cereals (−63.1 percentage points; 95% CI: −77.5, −48.6; *p*-value < 0.001), salty snacks (−13.2 percentage points, 95% CI: −20.2, −6.1; *p*-value < 0.001), and instant food (−26.5 percentage points; 95% CI: −36.5, −16.4; *p*-value < 0.001). Meanwhile reductions in T2 for “Excess saturated fat” occurred for cereal-based desserts (−12.5 percentage points; 95% CI: −17.3, −7.7; *p*-value < 0.001), salty snacks (−26.3 percentage points; 95% CI: −35.8, −16.8; *p*-value < 0.001), liquid dairy (−9.8 percentage points; 95% CI: −15.8, −3.8; *p*-value = 0.001), and solid dairy (−21.4 percentage points; 95% CI: −35.9, −6.9; *p*-value = 0.004). Finally, the reduction in non-caloric sweetener use in T2 was for cereal-based desserts (−11.5 percentage points; 95% CI: −16.4, −6.6; *p*-value < 0.001), liquid dairy (−14.5 percentage points; 95% CI: −24, −5; *p*-value = 0.003), solid dairy (−29 percentage points; 95% CI: −40.7, −17.2; *p*-value < 0.001), and candies (−6.6 percentage points; 95% CI: −11.5, −1.7; *p*-value = 0.008). Reductions in products above the warning-label cutoff for “Excess calories” in T2 were 27.9 percentage points in bread and other cereals (95% CI: −41.9, −14: *p*-value < 0.001) and 10.7 percentage points for sweetened beverages (95% CI: −15.9, −5.6; *p*-value < 0.001), with an equivalent reduction for the warning-label cutoff for “Excess sugars” for the latter group (−10.7; 95% CI: −15.9, −5.6; *p*-value < 0.001). The statistically significant reductions in the percentage of products above warning-label cutoffs in T2 tended to be above 10 percentage points, reaching up to 63.1 percentage points for the case of “Excess sodium” for bread and other cereals (95% CI: −77.5, −48.6; *p*-value < 0.001). Cereal-based desserts were the food group with the highest number of reductions of products whose calorie/nutrient content was above the respective warning-label cutoffs. However, all food groups experienced a statistically significant reduction in at least one kind of warning label in T2.

In general, reductions in the percentage of products above warning-label cutoffs were more frequent and larger in T2 than in T1. However, when comparing T1 versus T0, there was a reduction in the percentage of products above the warning-label cutoff for “Excess sugars” for bread and other cereals, and some percentage increases for some food groups (i.e., “Any warning label” for liquid dairy and non-caloric sweetener use for sweetened beverages), which turned statistically insignificant when comparing T2 versus T0. We observed that reductions in the percentage of products were more common when the relevant cutoff is close to or above the percentile 50th in T0, except for reductions in the case of “Excess sodium” for bread and other cereals, salty snacks, and instant food for which most of their products were above the sodium warning-label cutoff in T0.

**Fig 2:**
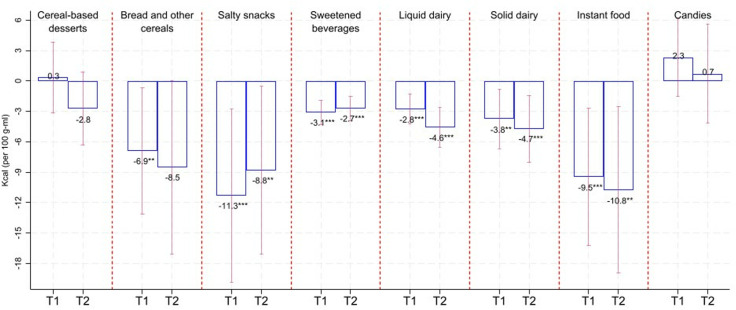
Change in calories compared to T0. Estimates based on linear fixed-effect-product regressions. Estimates are rounded to one decimal place. ***p-value < 0.01, **p-value < 0.05. Confidence intervals (95%) in red-capped spikes. Sample size: 670 observations for “Cereal-based desserts”, 131 observations for “Bread and other cereals”, 368 observations for “Salty snacks”, 489 observations for “Sweetened beverages”, 320 observations for “Liquid dairy”, 197 observations for “Solid dairy”, 285 observations for “Instant food”, and 366 observations for “Candies”.

**Fig 3:**
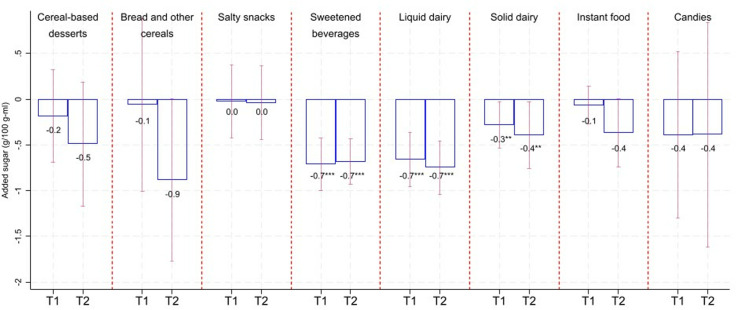
Change in added sugar compared to T0. Estimates based on linear fixed-effect-product regressions. Estimates are rounded to one decimal place. ***p-value < 0.01, **p-value < 0.05. Confidence intervals (95%) in red-capped spikes. Sample size: 670 observations for “Cereal-based desserts”, 131 observations for “Bread and other cereals”, 368 observations for “Salty snacks”, 489 observations for “Sweetened beverages”, 320 observations for “Liquid dairy”, 197 observations for “Solid dairy”, 285 observations for “Instant food”, and 366 observations for “Candies”.

**Fig 4:**
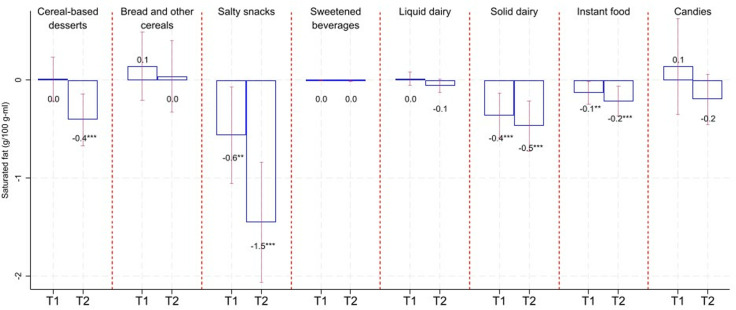
Change in saturated fat compared to T0. Estimates based on linear fixed-effect-product regressions. Estimates are rounded to one decimal place. ***p-value < 0.01, **p-value < 0.05. Confidence intervals (95%) in red-capped spikes. Sample size: 670 observations for “Cereal-based desserts”, 131 observations for “Bread and other cereals”, 368 observations for “Salty snacks”, 489 observations for “Sweetened beverages”, 320 observations for “Liquid dairy”, 197 observations for “Solid dairy”, 285 observations for “Instant food”, and 366 observations for “Candies”.

**Fig 5:**
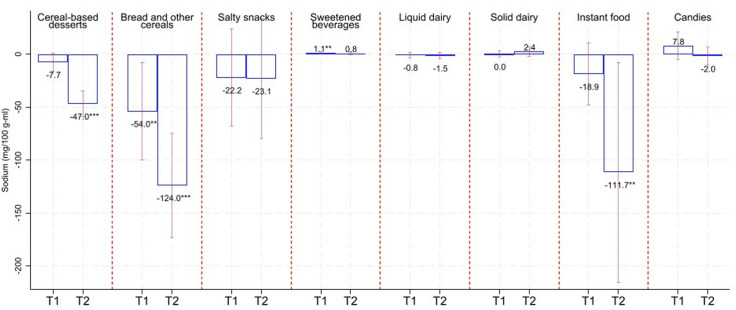
Change in sodium compared to T0. Estimates based on linear fixed-effect-product regressions. Estimates are rounded to one decimal place. ***p-value < 0.01, **p-value < 0.05. Confidence intervals (95%) in red-capped spikes. Sample size: 670 observations for “Cereal-based desserts”, 131 observations for “Bread and other cereals”, 368 observations for “Salty snacks”, 489 observations for “Sweetened beverages”, 320 observations for “Liquid dairy”, 197 observations for “Solid dairy”, 285 observations for “Instant food”, and 366 observations for “Candies”.

Figs 2–5 show the average changes in T1 and T2 compared to T0 by calorie/critical nutrient content and food group based on fixed effects models. Table F in S1 Text includes results as in Figs 1–4, adding 95% confidence intervals and p-values. Based on Figs 2–5, we see that calorie reductions in T1 and T2 ranged between 2.7 kcal/100 g-ml (sweetened beverages in T2; 95% CI: −3.9, −1.5; *p*-value < 0.001) and 11.3 kcal/100 g-ml (salty snacks in T1; 95% CI: −19.8, −2.8; *p*-value = 0.01) compared to T0. Bread and other cereals were the only group among those with statistically significant calorie reductions, with significant reductions only in T1. Reductions in added sugar ranged between 0.3 and 0.4 g/100 g-ml for solid dairy in T1 and T2 (*p*-value < 0.05) and 0.7 g/100 g-ml for sweetened beverages (*p*-value < 0.001) and liquid dairy (*p*-value < 0.001) in T1 and T2. The largest reduction in saturated fat was for salty snacks (−0.6 g/100 g in T1; 95% CI: −1.1, −0.1; *p*-value = 0.025; and −1.5 g/100 g in T2; 95% CI: −2.1, −0.8; *p*-value < 0.001); however, reductions were also statistically significant for solid dairy, instant food, and cereals-based desserts. Sodium content decreased for bread and other cereals in T1 (−54 mg/100 g; 95% CI: −100.3, −7.7; *p*-value = 0.023) and for cereal-based desserts, instant food, and bread and cereals in T2 ranging between −47 mg/100 g (for cereal-based desserts; 95% CI: −59.3, −34.7; *p*-value < 0.001) and −124 mg/100 g (for bread and other cereals; 95% CI: −173.3, −74.6; *p*-value < 0.001). It is worth noting that for all the reductions described above, these tended to be larger in T2 compared to those in T1. A small sodium increase for sweetened beverages in T1 turned statistically insignificant in T2.

The joint results from Figs 2 to 5 and Table 2 show that for calories and most of the critical nutrients for which we found statistically significant reductions, there was, as expected, a reduction in the proportion of products above their related warning-label cutoffs. Table F in S1 Text shows estimates of the changes in the calories and critical nutrient content distributions. Most of the significant average reductions were likely to be driven at the 50th and/or 75th percentile. For sodium for bread and other cereals and calories for sweetened beverages, reductions in T2 occurred across all analyzed percentiles. Table F in S1 Text also shows analyses when the outcomes are the percentage of energy coming from added sugar and saturated fat that, in general, resemble our main findings when analyzing added sugar and saturated fat as grams.

## Discussion

In this study, which is to our knowledge the first systematic analysis of product reformulation associated with warning labels in Mexico, we focus on assessing gradual changes in both the percentage of products with energy and critical nutrient content above warning-label cutoffs and the content of energy and critical nutrients for a set of packaged food and non-alcoholic beverages. We observed that after the warning-label policy implementation, there were significant reductions in the percentage of products that would be subject to “any warning label”, equivalent to an increase in products with no labels for salty snacks, sweetened beverages, solid dairy, and instant food. While all food groups had reductions in at least one type of warning label in T2, the most common reductions were for products above warning-label cutoffs for “Excess sodium”, “Excess saturated fat”, and containing non-caloric sweeteners. For salty snacks, the null use of non-caloric sweeteners in T2 suggests that the warning-label policy implementation was associated with a turnaround of the increasing non-caloric sweetener trend between T0 and T1. Most of the reductions in the content of calories and critical nutrients occurred at the 50th and/or 75th percentile of their distribution, except for sodium in bread and other cereals and calories in sweetened beverages, whose overall distributions shifted towards the left in T2. For salty snacks, solid dairy, and instant food, reductions in calories might result from their reduction in saturated fat due to no meaningful change in the percentage of products above the warning-label cutoff for “Excess calories”. While all the reductions described above occurred after the warning-label policy implementation in T2, we found that in T1 (i.e., once the policy was announced but not in place yet), there was a likely anticipated product reformulation before the policy implementation as there were reductions in calories and critical nutrients and in products above related warning-label cutoffs. However, our findings show that these reductions tended to be larger and more frequent in T2 than in T1. Overall, the findings of our study disagree with the food and beverage industry, which stated that the warning label policy in Mexico, the one with the most stringent nutrient criteria in Latin America, would not encourage product reformulation [12,36].

The variability in nutrient reformulation across different food groups can be attributed to factors such as health perception, technical feasibility, initial nutrient levels, and consumer preferences. Our findings indicate that reformulation was minimal in the food group of candies, likely because these products are already perceived as unhealthy, thus having fewer incentives for reformulation since warning labels are unlikely to alter their health perception [37]. In contrast, the food group of cereal-based desserts had significant reductions in sodium and saturated fat. Products with an intermediate perception of healthfulness, such as breakfast cereals (included in the category of cereal-based desserts), are more likely to be reformulated in response to warning labels, as manufacturers aim to make them more appealing to health-conscious consumers. Technical feasibility also impacts reformulation. In certain products, specific nutrients are critical for maintaining structure and taste. For instance, sugar is a primary ingredient in most candies, making significant reductions unlikely without compromising the product’s flavor and characteristics. Additionally, there are practical limits to how much certain nutrients can be reduced. For example, in the United Kingdom, bread was reformulated after the implementation of a salt reduction program from a mean content of 450–392 mg of sodium per 100 g [38]. In the context of our study, these results might entail a potential floor effect for sodium reductions. Moreover, further sodium cuts might be infeasible as salt is required in bread for texture and flavor. Similar constraints likely exist for other nutrients and products. Additionally, products with inherently low levels of certain nutrients, like sodium and saturated fat in sweetened beverages, show minimal potential for further reduction. Finally, consumer preferences might also play a significant role, as reformulations must balance the reduction of critical nutrients with consumers’ acceptance [39].

Our results point in the same direction as those of Chile and Peru, early adopters of the warning label system, showing reductions of critical nutrients and products above warning-label cutoffs. However, differences emerge across countries in the patterns of these reductions. For example, in Chile, reductions in the warning label for sugar were the most common reduction by type of warning label, occurring among 5 out of the 16 analyzed food-beverage groups [15]. Likewise, in Peru, there was a significant reduction in the proportion of products that would be subject to warning label for sugar across the analyzed food-beverage groups after the policy implementation [18]. Conversely, reductions in products above the warning-label cutoff for “Excess sugar” in our study were restricted to cereal-based desserts and sweetened beverages. This difference in findings could result from the fact that warning label systems in Chile and Peru do not target non-caloric sweeteners. Specifically, evidence in these countries pointed towards a potential sugar substitution with non-caloric sweeteners, considering an increase in their use after the policy implementation [18,40]. Meanwhile, our findings showed either reductions or no increases in the use of non-caloric sweeteners. Another important difference in results between Chile and Mexico is the warning label for saturated fat. For the former country, only one food group (i.e., savory spreads) reached significant reductions in the warning label for saturated fat [15]; meanwhile, four food groups in Mexico (cereal-based desserts, salty snacks, liquid dairy, and solid dairy) reported equivalent reductions for the same warning label. We pose that this difference can arise from the fact that a lower percentage of products exceed the warning-label cutoff for saturated fat in Chile (in general, <25%) compared to the equivalent figure in Mexico (in general, >50%). Thus, there are fewer products in Chile for which producers are incentivized to reformulate. The lower percentage of products in Chile exceeding the warning-label cutoff for saturated fat compared to Mexico could result from Mexico’s warning-label cutoffs tending to be more rigorous than those in Chile [12]. Previously, it has been shown that Chilean warning-label cutoff criteria applied to the packaged food and beverage market in Mexico would lead to fewer products with warning labels compared to the scenario under the Mexican warning-label cutoff criteria [12]. It is worth noting that all food groups, except one (liquid dairy in Mexico), with reductions in the warning label for saturated fat in Chile and Mexico had a baseline percentage of products subject to that warning label close to 50%.

Regarding similarities in reformulation results between Chile and Mexico, reductions in products exceeding the warning-label cutoff for sodium were common across several food groups in both countries. However, relative reductions tend to be larger in Chile, ranging between 40% and 65% [15]. Conversely, in Mexico, some groups had relative reductions equivalent to less than 40% (e.g., salty snacks and instant food), which contrasted with other groups’ reductions of around 60%–70% (e.g., cereal-based desserts, bread, and other cereals). The larger reductions in Chile could result from less restrictive warning-label cutoffs for sodium compared to Mexico. Specifically, in the first stage of the warning label policy, this cutoff for food was 800 mg/100 g in Chile and 350 mg/100 g in Mexico [12,15]. Thus, it could have been less challenging in Chile to place more products’ sodium content below the relevant warning-label cutoff. Finally, it is worth highlighting that our results from assessing reductions over the critical nutrient distribution are similar to those in Chile. While we found most of these reductions occurred at the 50th–75th percentiles, evidence in Chile places these changes for warning-label cutoffs below the 75th percentile of the relevant nutrient of concern distribution [15].

Evidence of reformulation from mandatory food policies other than warning labels comes from sugar-density taxes for sugar-sweetened beverages. For the multi-tiered sugar tax in the United Kingdom, evidence shows a reduction in the proportion of products with a sugar content ≥ 5 g/100 ml, corresponding to the first threshold to be subject to a tax (i.e., £0.18 per liter) [25]. The tax burden gets higher with sugar content > 8 g/100 ml (i.e., £0.24 per liter) [25]. Meanwhile, descriptive statistics suggest that the sugar-density tax for sugar-sweetened beverages in South Africa, which sets a 0.021 ZAR tax per each additional gram over 4 g/100 ml, prompted reformulation over the sugar distribution [21]. It is challenging to determine how our findings compare to reformulation under sugar-density taxes because these taxes can encourage reformulation around more than one cutoff (e.g., the multi-tier tax in the United Kingdom) or over the full sugar distribution (e.g., the continuous sugar-density tax in South Africa). Meanwhile, reformulation linked to warning labels in Mexico is likely to be primarily set around the respective warning-label cutoffs.

Regarding the potential timing of the reformulation, evidence in Chile shows no major early reformulation in light of an average reduction of 1% in calories and critical nutrients before the warning-label policy implementation, which would entail less than 2% of products avoiding warning labels [17]. Meanwhile, evidence from other nutrient-profile policies showed reformulation for breakfast cereals in Belgium (sugar and sodium reductions coupled with fiber and protein rises) and energy drinks in the United Kingdom (reductions in sugar of 10%) before the implementation of the voluntary Nutri-Score labels and a mandatory multi-tiered sugar tax, respectively [24,41]. However, the study’s authors in Belgium acknowledged that other industry-led reformulation initiatives were in place simultaneously with the Nutri-Score label policy, so their findings might not be necessarily linked to this policy [41]. Another study assessed product reformulation under the mandatory multi-tiered sugar tax in the United Kingdom based on a strong empirical approach (i.e., interrupted time series analyses complemented with a control group) and comprehensive data spanning close to 90 months, which included pre-policy-announcement, post-policy-announcement, and post-policy-implementation information [25]. These rich data allowed the authors to simulate a counterfactual trend in the absence of the policy, compared to which there was a reduction in the proportion of sugar-sweetened beverages with a sugar content subject to the tax in advance of the tax implementation, with this reduction turning larger once the tax came into effect [25]. These results were robust when compared to the control group trend [25]. Our anticipatory reformulation results before the policy implementation (i.e., when comparing T1 versus T0) are in the range of studies described above because while we saw no reformulation for some food groups, we observed some reductions in calorie and critical nutrient content and in products above warning-label cutoffs. However, after the warning-label policy implementation in T2, these reductions tended to be larger and more frequent across food groups. These latter results are consistent with the evidence from the mandatory multi-tiered sugar tax for sugar-sweetened beverages in the United Kingdom regarding a larger extent of product reformulation once the policy is in place [25].

Even though the total potential influence of warning labels on nutrient intake remains to be assessed through mechanisms, such as usage of the information conveyed by warning labels and marketing package restrictions, product reformulation is likely to play a major role in dietary intake changes associated with warning labels. Findings from an experimental study in Ireland showed that labels in that study, the Nutri-score system, which provides color-coded nutritional information, coupled with product reformulation could double the magnitude of the improvement in the healthfulness of a set of purchased snack foods compared to the scenario of labels and no reformulation [42]. For other nutrient-focused policies, such as sugar-based taxes for sugar-sweetened beverages, reformulation explained around 30% and 80% of the total reduction in sugar intake for South Africa and the United Kingdom, respectively [22,23]. In Mexico, simulation-based studies have shown the potential of sugar-density taxes for sugar-sweetened beverages coupled with reformulation (i.e., sugar reduction) to yield larger sugar intake drops and health gains compared to a scenario where this kind of tax does not prompt reformulation [43,44]. Second-income quintile households in Mexico are likely to experience the largest health gains from sugar-sweetened beverages taxes due to their higher baseline obesity prevalence and larger responsiveness to sugar-sweetened beverage price increases [44]. How the reformulation linked to warning labels would differentially impact diet outcomes across households that vary in income level remains to be explored. Evidence in Mexico shows that per capita calories from non-essential energy-dense foods and any kind of beverage hold a positive relationship with households’ income level; however, the lowest income households reported the highest increase in these calories in the period 1992–2016 [45]. Hence, reformulation linked to warning labels could be more beneficial for low-income households in Mexico by potentially mitigating their increasing trend in calorie intake from non-essential energy-dense foods and any kind of beverage. However, more research is needed to assess this mechanism in addition to a differential use of warning labels across households. In this latter regard, it is worth noting that no massive communication campaign was in place a year and a half after the implementation of the warning labels to promote their use [46].

Our study exhibits some limitations. First, our study is not representative of the overall packaged food and beverage market in Mexico because NutrINSPector focused on top products, representing at least 60% of purchases of their respective markets in 2016. This limitation arose from the COVID-19 lockdown in which NutrINSPector’s first data collection took place, which limited the inclusion of more products amid the restrictive conditions for in-person shopping. Second, our statistical models did not account for products’ market-share weights, which implies a homogenous weight across all products. This limitation is common in other studies assessing reformulation in the context of warning labels [15,40]. This limitation might not be restrictive because we focus on top-purchased products whose differences in market shares are likely to be less marked than the differences between top and less-demanded products. Third, due to the data collection criteria of NutrINSPector (i.e., top-purchased products in 2016), the data do not capture new products launched after the warning-label implementation that are likely to have a better nutritional profile to avoid displaying warning labels. Fourth, we acknowledge that measurement errors can arise from using the Pan American Health Organization approach to calculate added sugar and randomly selecting a product for merging the 2016−2017 dataset with NutrINSPector when merging based on the universal product code was not possible. While differences can exist between the Pan American Health Organization’s estimated values and a product’s actual sugar content, this approach is evidence-based and aligned with models used in other regions. Meanwhile, random product selection for merging purposes maintains data completeness and comparability across food groups with no arbitrary selection criteria. Hence, while the use of the Pan American Health Organization approach and the random product selection in the 2016−2017 data could introduce some imprecision, they reduce potential systematic bias, strengthening the reliability of our findings. Finally, the short time span of our analytical data, with only three periods, prevents us from setting a more comprehensive model to account for any underlying time trends in products’ calorie and nutrient content. This contrasts with the study by Scarborough and colleagues, where the authors set an interrupted time series analysis to assess the multi-tier sugar tax for sugar-sweetened beverages in the United Kingdom [25]. Hence, in light of our data structure and model specifications, our last study’s limitation is that we cannot claim our results entail causality. Specifically, the food industry reported product reformulation before the announcement of the warning label policy [26]. Nonetheless, product reformulation is a constant in the food industry, with new products and packaging launches occurring periodically. Although changes in critical nutrients in this study cannot be entirely attributable to the warning-label implementation, our results show, on average, an improvement in the nutrient profile of packaged products, shifting distributions in some food groups very close to the warning-label cutoffs.

From a public health perspective, our results are relevant in several regards. First, we showed an immediate improvement in the nutritional profile of the packaged food and beverage supply after the warning-label implementation in Mexico. Thus, even the roughly 30% of Mexicans who did not read warning labels in 2021 when purchasing packaged food and beverages will benefit from this nutritional profile improvement [47]. Second, Mexico was the first country whose warning labels included caffeine and non-caloric sweeteners. While we saw no change in caffeine use, we found meaningful reductions in non-caloric sweetener use in cereal-based desserts, liquid dairy, solid dairy, and candies. Moreover, the increasing non-caloric sweetener use in T1 versus T0 for sweetened beverages turned to no differential use in T2 versus T0. We observed a similar pattern for salty snacks; however, we could not test its statistical significance. These results suggest that the warning-label design in Mexico could discourage non-caloric sweetener use, and thus avoid potential product reformulation via the substitution of sugar for non-caloric sweeteners, as in Peru and Chile [18,40]. The World Health Organization has recently recommended not consuming NCS due to their associated adverse health outcomes [48]. Finally, the bunching of some products’ critical nutrient content right below the relevant warning-label cutoff for cereal-based desserts and bread and other cereals suggests that producers are sensitive to this cutoff. Due to the warning-label cutoffs being more stringent for the second phase of the warning-label norm [9], producers could likely keep reducing the content of critical nutrients, continuing to improve the food and beverage options available in Mexico. Therefore, future studies should keep monitoring the packaged food and beverage supply and how this supply evolves in response to upcoming updates in the warning-label cutoffs.

Moreover, future evaluations should assess reformulation in other components of ultra-processed foods, such as cosmetic additives and the addition of new sweeteners, flavorings, or preservatives, which may not necessarily be considered favorable consequences of the warning-label implementation. Nevertheless, it remains to be explored whether these modifications have led to products becoming more ultra-processed, albeit with fewer warning labels, and whether the warning labels have motivated individuals to modify their purchasing behaviors and reduce their intake of ultra-processed foods. However, the reductions in critical nutrients observed in our study represent a positive step towards healthier nutrient profiles in the food supply, independent of consumers’ behavior due to the potential short-term population benefits, especially in contexts like Mexico, where high rates of obesity and chronic diseases require urgent action. Finally, while the focus of our study is on product reformulation, evidence shows that this reformulation in the context of food policies such as warning labels or sugar-sweetened beverage taxes can be coupled with price adjustments due to likely upward production costs linked to reformulation [43,49]. Thus, future studies should extend the analysis of warning labels to assess other firm responses (e.g., price or marketing adjustments) and their implications for households’ food/beverage purchase decisions.

## Conclusions

In this study, we found that after the implementation of the warning label policy, there was a potential reformulation to healthier products in light of the reduction in the percentage of products above warning-label cutoffs in tandem with reductions in products’ energy and critical nutrient contents. While all analyzed food groups had reductions in at least one type of warning label, the most common ones were for products above warning-label cutoffs for sodium and saturated fat and the use of non-caloric sweeteners. Regarding the latter result, our study differs from those in Chile and Peru, early adopters of the warning label policy, where there was an increase in non-caloric sweeteners as a potential sugar substitution as these countries do not target this kind of sweeteners as part of their warning-label systems. The product reformulation after the warning-label policy implementation in Mexico entails an improvement in the nutritional profile in the supply of packaged food and non-alcoholic beverages. Future studies should assess how this nutritional improvement translates to population diet and health gains.

## Supporting information

S1 Text Supplemental appendix. (DOCX)

S1 Data Analytical data. (DTA)

S1 Code Code for binary outcomes. (DO)

S2 Code Code for continuous outcomes. (DO)
