# Supplementary material for: Product reformulation in non-alcoholic beverages and foods after the implementation of front-of-pack warning labels in Mexico
Source: PLoS Med. 2025 Mar 18;22(3):e1004533. doi: 10.1371/journal.pmed.1004533 (PMC11918434; doi:10.1371/journal.pmed.1004533)
Supplement: S1 Text — (DOCX) [file pmed.1004533.s001.docx]

**S1 Text: Supplemental appendix for “Product reformulation in non-alcoholic beverages and foods after the implementation of front-of-pack warning labels in Mexico”**

**Table of contents**

[File A. Steps for Constructing the Study’s Analytical Data 2](#_Toc188206101)

[Table A. Dropped observations in NutrINSPector 3](#_Toc188206102)

[Table B. Analytical data by food group 4](#_Toc188206103)

[Table C. Subcategories by food group 6](#_Toc188206104)

[Table D. Density factors 7](#_Toc188206105)

[Table E. Percentile for warning-label cutoff 8](#_Toc188206106)

[Figure A. Kernel densities of calories and critical nutrients for cereal-based desserts 9](#_Toc188206107)

[Figure B. Kernel densities of calories and critical nutrients for bread and other cereals 10](#_Toc188206108)

[Figure C. Kernel densities of calories and critical nutrients for salty snacks 11](#_Toc188206109)

[Figure D. Kernel densities of calories and critical nutrients for sweetened beverages 12](#_Toc188206110)

[Figure E. Kernel densities of calories and critical nutrients for liquid dairy 13](#_Toc188206111)

[Figure F. Kernel densities of calories and critical nutrients for solid dairy 14](#_Toc188206112)

[Figure G. Kernel densities of calories and critical nutrients for instant food 15](#_Toc188206113)

[Figure H. Kernel densities of calories and critical nutrients for candies 16](#_Toc188206114)

[Table F. Changes in calories and critical nutrients compared to T0 17](#_Toc188206115)

[References 20](#_Toc188206116)

## **File A.** Steps for Constructing the Study’s Analytical Data

We conducted the next methodological steps to get our analytical data.

1. Before the warning-label implementation, reporting added sugars and trans-fat was optional for producers [1]. Moreover, producers could round down trans-fat to zero when trans-fat contributed to less than five calories per serving [2]. Thus, we approximated added sugars using the Pan-American-Health-Organization Nutrient Profile Model approach (Panel D) in T0-T2 and excluded trans-fat from our analyses due to the high presence of zeros or missing data (around 87% in the analytical data) [3].
2. We reconstituted dry and not-ready-to-consume products (e.g., drink mix or instant soups) according to their preparation instructions to obtain nutritional information for their as-consumed form and double-checked data capture.
3. Based on density factors, we transformed products’ units to grams when their nutritional information was in milliliters and they were part of a solid food group. We did the opposite for products in grams in a beverage group. Table D includes information on relevant density factors.
4. We standardized all products’ nutritional information per 100 g or ml. Based on this information, we double-checked data capture when there were outliers in the density distributions of the standardized content, which we defined as those located at a distance from the main concentration of data. This resulted in the data capture (full or partial) of 11 observations.
5. Based on information from point four above, we determine the respective warning labels in T0-T2 following the nutrient profile for the first phase of the regulation (from October 2020 to September 2023), as shown in Fig 1 in the main manuscript. For the application of this nutrient profile, we used the criteria for food in all our food groups, except for the groups of sweetened beverages and liquid dairy, for which we used the criteria for beverages. The warning-label legends for non-caloric sweetener content and added caffeine were based on the ingredients list at the product level.
   1. When calculating the warning label of “Excess sodium” for products reporting salt content rather than sodium, we assumed that a gram of salt equals 400 mg of sodium.
   2. When calculating the warning labels of “Excess calories”, “Excess sugars”, and “Excess saturated fat”, which depend on energy from specific critical nutrients, we set a gram of added sugar equivalent to four calories and a gram of saturated fat equivalent to nine calories. We set a zero percent energy contribution from added sugar or saturated fat for products with no added sugar or no added fat, respectively. For fifteen observations whose added sugar contribution to energy was higher than 100% (ranging between 100.3 and 105.3%), we redefined it as 100%.
6. We excluded products with no added sugar/sodium/fat/non-caloric-sweetener/caffeine for all periods with available information (composed mainly of natural oats, plain cow’s milk, and pasta), not subject to the policy (e.g., infant formula), had missing information (see details in Table A), or had information only in T1 or T2.

We set “Step 3” to allow comparability in terms of presentation (i.e., food or liquid) across products within a food group, and thus allowing comparability when identifying which products were above related warning-label cutoffs for calories and critical nutrients according to the description in “Step 5”. Meanwhile, measures described in “5a” and “5b” are standard in the nutrition field.

## **Table A.** Dropped observations in NutrINSPector

| **NutrINSPector: Aug-Sep 2020 (T1)** | **NutrINSPector: Feb-Apr 2021 (T2)** |
| --- | --- |
| - 23 duplicated observations. - 1 observation because it is a milk formula, which is not subject to front-of-pack-warning-label policy. - 6 observations because it changes its reconstitution formula over time. - 41 observations with missing values for all nutrients per 100 grams. - 3 observations without enough information to be reconstituted. - 2 observations with incomplete information on at least one nutrient of concern/calories to assign the warning labels for labeling (excluding saturated fats) or non-nutritive sweeteners/caffeine. - 59 observations for products with no added sugar nor added fat nor added sodium for all periods with available information (mainly composed of natural oats, unflavored milk, pasta, and 100% natural juices). - 9 observations for products with information only in T1. | - 33 duplicated observations - 1 observation because it is a milk formula, which is not subject to front-of-pack-warning-label policy. - 3 observations because they are infant juice, which are not subject to front-of-pack-warning-label policy. - 5 observations because it changes its reconstitution formula over time. - 5 observations without enough information to be reconstituted. - 3 observations with incomplete information on at least one nutrient of concern/calories to assign the warning labels for labeling (excluding saturated fats) or non-nutritive sweeteners/caffeine. - 56 observations for products with no added sugar nor added fat nor added sodium for all periods with available information (mainly composed of natural oats, unflavored milk, pasta, and 100% natural juices). - 47 observations for products with information only in T2. |

## **Table B.** Analytical data by food group

| **Food group** | **Number of products and sample size** |
| --- | --- |
| Cereal-based desserts | - Number of products: 241 products, for which 188 had information in T0-T2, 20 in T0 and T1, 20 in T0 and T2, and 13 in T1 and T2. - Total sample size is 670 observations. From these observations, 212 observations are from the 2016-17 data, 14 from OFF Mexico, 2 from OFF world, and 442 from NutrINSPector. Finally, 228 observations are for T0, 221 for T1, and 221 for T2. |
| Bread and other cereals | - Number of products: 48 products, for which 35 had information in T0-T2, 4 in T0 and T1, 4 in T0 and T2, and 5 in T1 and T2. - Total sample size is 131 observations. From these observations, 33 observations are from the 2016-17 data, 6 from OFF Mexico, 4 from OFF world, and 88 from NutrINSPector. Finally, 43 observations are for T0, 44 for T1, and 44 for T2. |
| Salty snacks | - Number of products: 142 products, for which 84 had information in T0-T2, 14 in T0 and T1, 37 in T0 and T2, and 7 in T1 and T2. - Total sample size is 368 observations. From these observations, 125 observations are from the 2016-17 data, 9 from OFF Mexico, 1 from OFF world, and 233 from NutrINSPector. Finally, 135 observations are for T0, 105 for T1, and 128 for T2. |
| Sweetened beverages | - Number of products: 181 products, for which 127 had information in T0-T2, 11 in T0 and T1, 38 in T0 and T2, and 5 in T1 and T2. - Total sample size is 489 observations. From these observations, 167 observations are from the 2016-17 data, 8 from OFF Mexico, 1 from OFF world, and 313 from NutrINSPector. Finally, 176 observations are for T0, 143 for T1, and 170 for T2. |
| Liquid dairy | - Number of products: 125 products, for which 70 had information in T0-T2, 6 in T0 and T1, 39 in T0 and T2, and 10 in T1 and T2. - Total sample size is 320 observations. From these observations, 103 observations are from the 2016-17 data, 12 from OFF Mexico, 0 from OFF world, and 205 from NutrINSPector. Finally, 115 observations are for T0, 86 for T1, and 119 for T2. |
| Solid dairy | - Number of products: 71 products, for which 55 had information in T0-T2, 2 in T0 and T1, 9 in T0 and T2, and 5 in T1 and T2. - Total sample size is 197 observations. From these observations, 58 observations are from the 2016-17 data, 8 from OFF Mexico, 0 from OFF world, and 131 from NutrINSPector. Finally, 66 observations are for T0, 62 for T1, and 69 for T2. |
| Instant food | - Number of products: 102 products, for which 81 had information in T0-T2, 5 in T0 and T1, 13 in T0 and T2, and 3 in T1 and T2. - Total sample size is 285 observations. From these observations, 95 observations are from the 2016-17 data, 3 from OFF Mexico, 1 from OFF world, and 186 from NutrINSPector. Finally, 99 observations are for T0, 89 for T1, and 97 for T2. |
| Candies | - Number of products: 141 products, for which 84 had information in T0-T2, 16 in T0 and T1, 32 in T0 and T2, and 9 in T1 and T2. - Total sample size is 366 observations. From these observations, 125 observations are from the 2016-17 data, 6 from OFF Mexico, 1 from OFF world, and 234 from NutrINSPector. Finally, 132 observations are for T0, 109 for T1, and 125 for T2. |
| Note: OFF stands for Open Food Facts. | |

## **Table C.** Subcategories by food group

| **Food group** | **Food subcategories: percentage contribution in terms of observations at the food-category level** |
| --- | --- |
| Cereal-based desserts | - Cereal bars: 14.0% - Cookies: 41.2% - Flavored oatmeal: 5.4% - Ready-to-eat cereals and granola: 23.3% - Pastry: 16.1% |
| Bread and other cereals | - Bread (sliced bread, toast, hamburger buns, and hot-dog buns): 90.8% - Breadcrumbs and flour tortillas: 9.16% |
| Salty snacks | - Potatoes chips, cereal-based chip/toast, popcorns, and peanuts/seeds: 94.8% - Crackers: 5.2% |
| Sweetened beverages | - Regular/light soft drinks, ready-to-drink coffee and tea, fruit-based beverages, energy drinks, and flavored water: 77.7% - Drinx mix, coffee, tea, and sugar substitute: 22.3% |
| Liquid dairy | - Chocolate-powder-based milk: 14.1% - Coffee creamer: 8.7% - Drinkable yogurt, dairy products (milk substitute, evaporated milk, probiotic milk beverage), almond/soy-based drinks, flavored milk, and milkshakes: 77.2% |
| Solid dairy | - Solid yogurt, petit-suisse-cheese-based food, milk-based desserts (natilla, flan, rice pudding), and creams: 100% |
| Instant food | - Canned food (vegetables and tuna): 38.6% - Instant food (soups and powdered chicken/rice bouillon): 34.0% - Sauce and tomato puree: 27.4% |
| Candies | - Candies (chocolates, jellies, candies): 38.8% - Ice creams (ice pops and ice creams): 39.3% - Spreads (marmalade, peanut butter, cocoa spread, corn syrup, condensed milk, goat's milk caramel, chocolate syrup): 21.9% |

## **Table D.** Density factors

| **Food group** | **Density factors** |
| --- | --- |
| Liquid dairy | From grams to ml  Drinkable yogurt, probiotic milk beverage, and milkshakes: 1.02 g/ml (Source: USDA-FNDDS) [4].  Evaporated milk: 1.07 g/ml (Source: NutrINSPector products’ pictures). |
| Solid dairy | From ml to grams   - Creams: 0.97 g/ml (Source: USDA and supermarket’s products’ pictures) [4,5]. - Table cream (media crema): 1 g/ml (Source: USDA-FNDDS and supermarket’s products’ pictures) [4,5]. |
| Instant food | From ml to grams   - Sauces: between 1.07 and 1.13 g/ml depending on the kind of sauce (i.e., soy, hot, Worcestershire) (Source: USDA-FNDDS) [4]. - Reconstituted condensed soup: between 1.025 and 1.075 g/ml depending on the kind of soup (Source: USDA-FNDDS).[4] - Reconstituted powdered broth: 1.06 g/ml (Source: FAO/INFOODS Databases) [6]. - Reconstituted instant soup: 1 g/ml (Source: FAO/ INFOODS Databases) [6]. |
| Candies | From ml to grams:   - Ice cream: between 0.53 and 1.11 g/ml depending on the product (Source: NutrINSPector products’ pictures and USDA-FNDDS) [4]. - Condensed milk: 1.33 g/ml (Source: NutrINSPector products’ pictures). - Chocolate syrup: 1.3 g/ml (Source: NutrINSPector products’ pictures). |
| Note: USDA-FNDDS stands for United States Department of Agriculture’s Food and Nutrients Database for Dietary Studies, and FAO stands for Food and Agricultural Organization. | |

## **Table E.** Percentile for warning-label cutoff

|  | **T0** | **T1** | **T2** |
| --- | --- | --- | --- |
| **Outcome** | **Percentile** | **Percentile** | **Percentile** |
| **Cereal based desserts** | **n= 228 obs** | **n= 221 obs** | **n= 221 obs** |
| Excess calories | 6 | 6 | 8 |
| Excess sugar | 8 | 7 | 11 |
| Excess saturated fat | 46 | 44 | 59 |
| Excess sodium | 52 | 58 | 81 |
| **Bread and other cereals** | **n= 43 obs** | **n= 44 obs** | **n= 44 obs** |
| Excess calories | 51 | 64 | 70 |
| Excess sugar | 51 | 64 | 57 |
| Excess saturated fat | 91 | 95 | 95 |
| Excess sodium | 7 | 30 | 68 |
| **Salty snacks** | **n= 135 obs** | **n= 105 obs** | **n= 128 obs** |
| Excess calories | 1 | 1 | 1 |
| Excess sugar | 94 | 91 | 94 |
| Excess saturated fat | 39 | 53 | 66 |
| Excess sodium | 13 | 17 | 26 |
| **Sweetened beverages** | **n= 176 obs** | **n= 143 obs** | **n= 170 obs** |
| Excess calories^a^ | 99 | 99 | 99 |
| Excess sugar^a^ | 28 | 30 | 29 |
| Excess saturated fat | 97 | 99 | 97 |
| Excess sodium^a^ | 99 | 99 | 99 |
| **Liquid dairy** | **n= 115 obs** | **n= 86 obs** | **n= 119 obs** |
| Excess calories^a^ | 63 | 71 | 78 |
| Excess sugar^a^ | 20 | 17 | 22 |
| Excess saturated fat | 80 | 77 | 90 |
| Excess sodium^a^ | 99 | 99 | 99 |
| **Solid dairy** | **n= 66 obs** | **n= 62 obs** | **n= 69 obs** |
| Excess calories | 94 | 95 | 99 |
| Excess sugar | 30 | 37 | 30 |
| Excess saturated fat | 52 | 85 | 72 |
| Excess sodium | 99 | 99 | 99 |
| **Canned food** | **n= 99 obs** | **n= 89 obs** | **n= 97 obs** |
| Excess calories | 98 | 99 | 99 |
| Excess sugar | 78 | 75 | 84 |
| Excess saturated fat | 75 | 78 | 82 |
| Excess sodium | 34 | 42 | 62 |
| **Candies** | **n= 132 obs** | **n= 109 obs** | **n= 125 obs** |
| Excess calories | 46 | 47 | 46 |
| Excess sugar | 11 | 7 | 10 |
| Excess saturated fat | 45 | 39 | 43 |
| Excess sodium | 89 | 91 | 88 |
| ^a^Beverages are also subject to the following criteria: when ≥ 10 calories come from free sugar, the product has to display the warning label of "excess calories"; when less than 10 calories come from free sugar, the product is exempted from displaying the warning label of "excess sugar"; when ≥ 45 mg sodium/ 100ml and the product has no calories, the product has to display the warning label of "excess sodium". | | | |

## **Figure A.** Kernel densities of calories and critical nutrients for cereal-based desserts

| **a)**  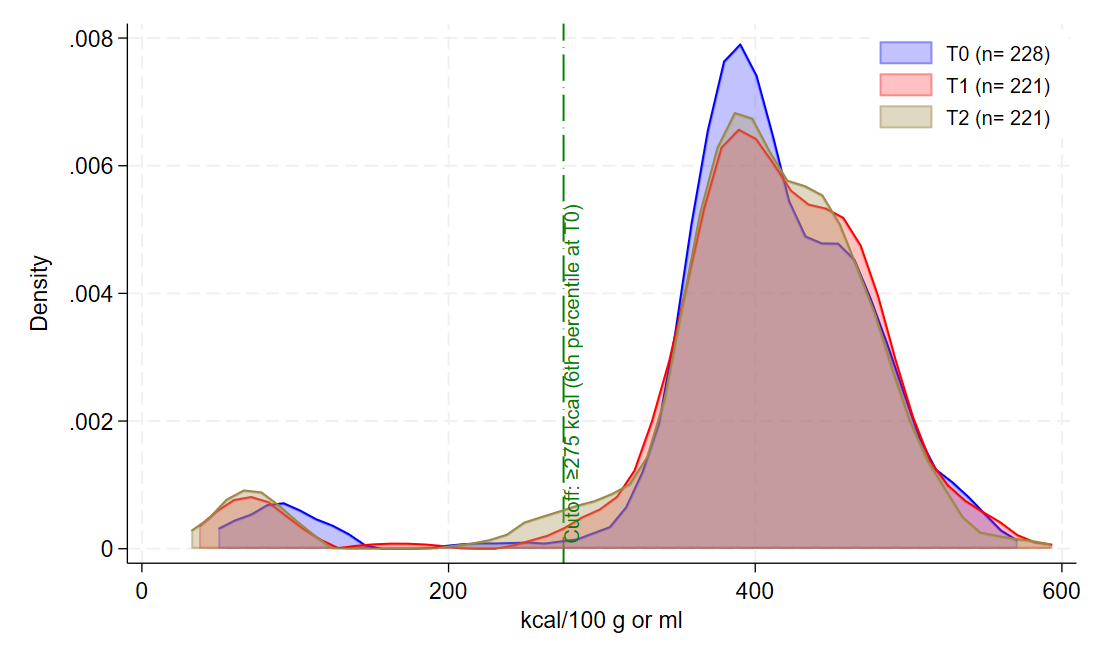 | **b)**  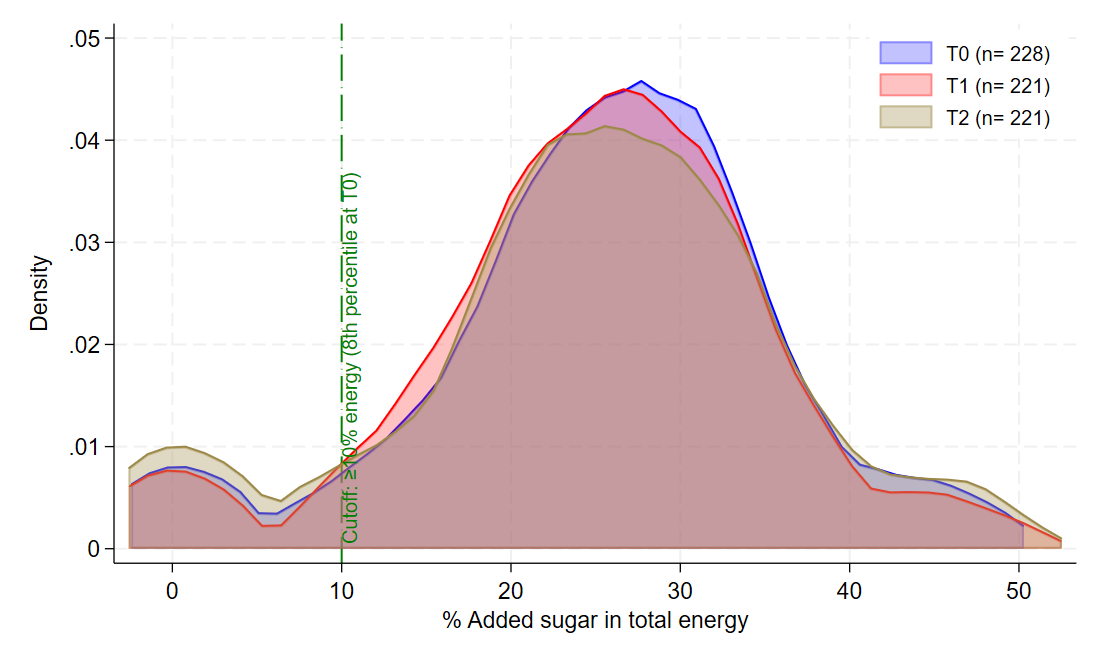 |
| --- | --- |
| **c)**  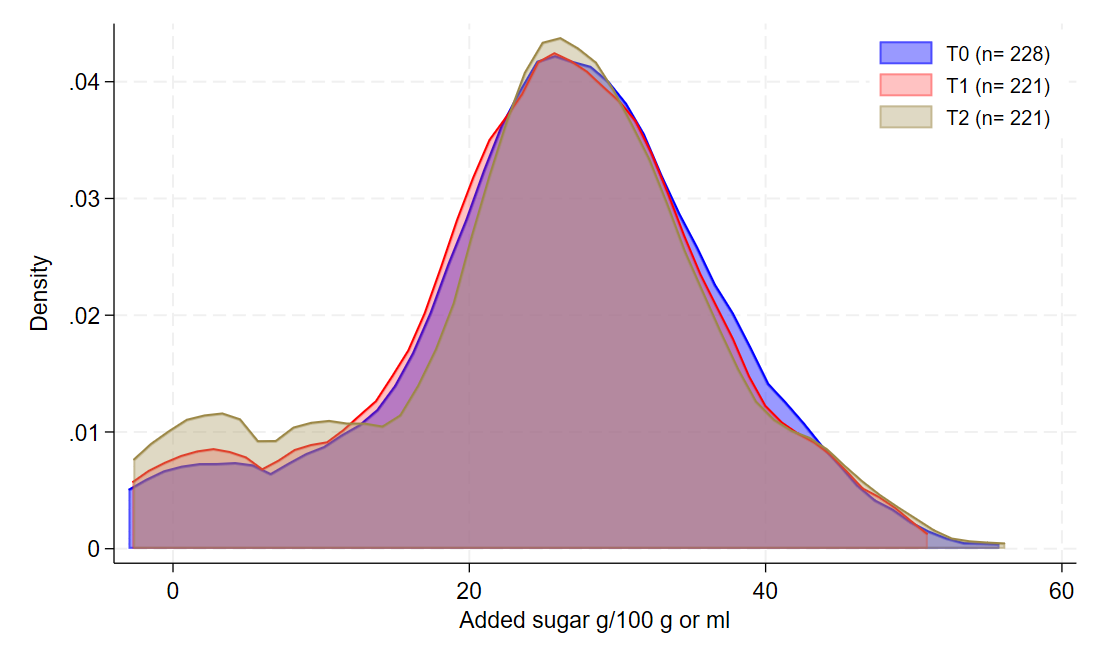 | **d)**  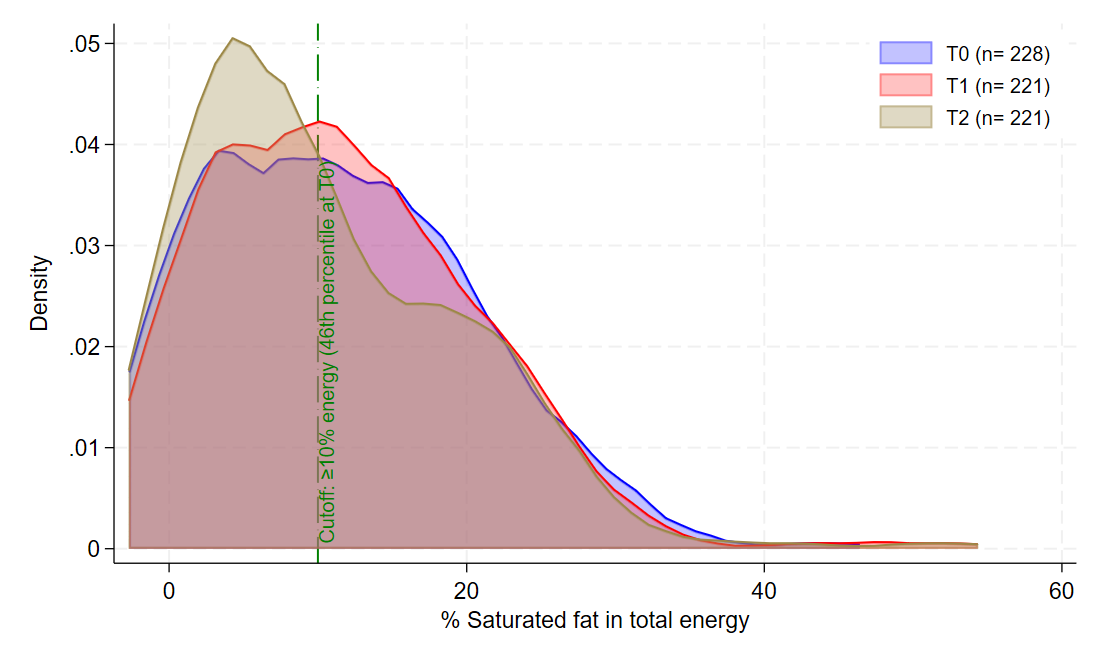 |
| **e)**  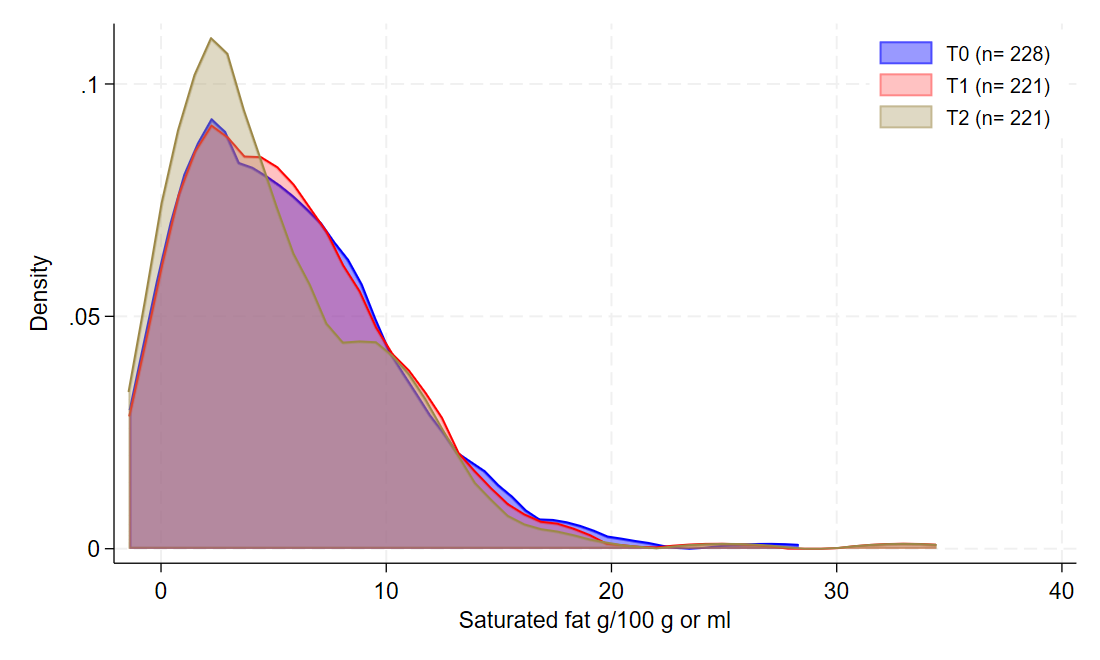 | **f)**  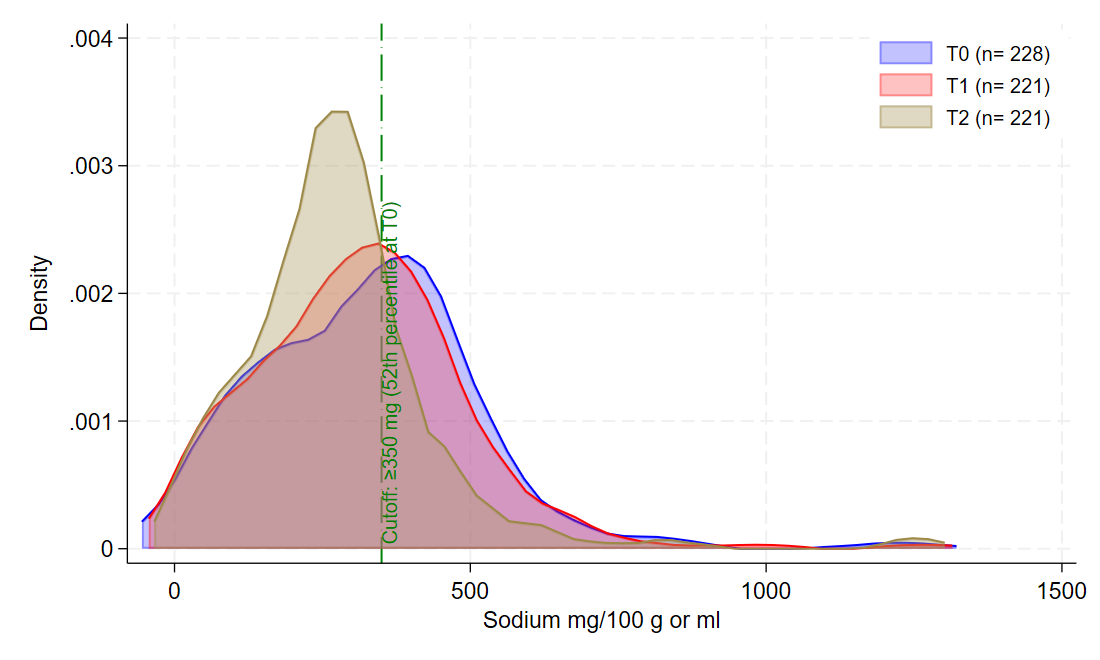 |
| **Note:** a) kcal/100 g or ml, b) Added sugar as % of total energy, c) Added sugar g/100 g or ml, d) Saturated fat as % of total energy, e) Saturated fat g/100 g or ml, f) Sodium mg/100 g or ml. All cutoffs are based on the criteria of the first stage of the front-of-package warning labels, as described in Table 1. | |

## **Figure B.** Kernel densities of calories and critical nutrients for bread and other cereals

| **a)**  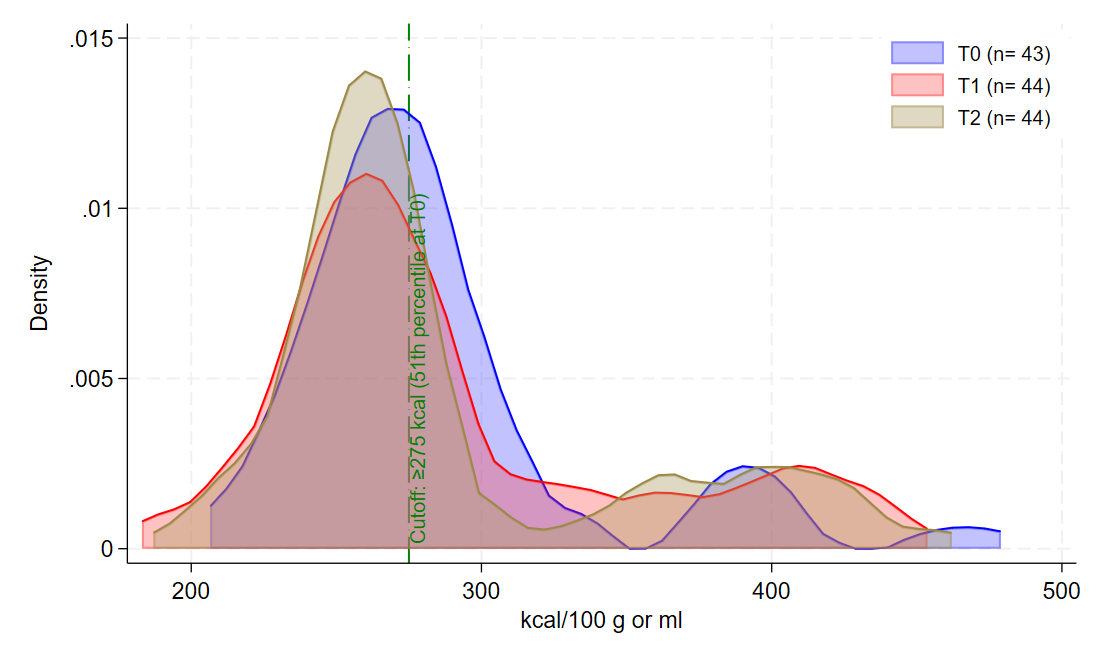 | **b)**  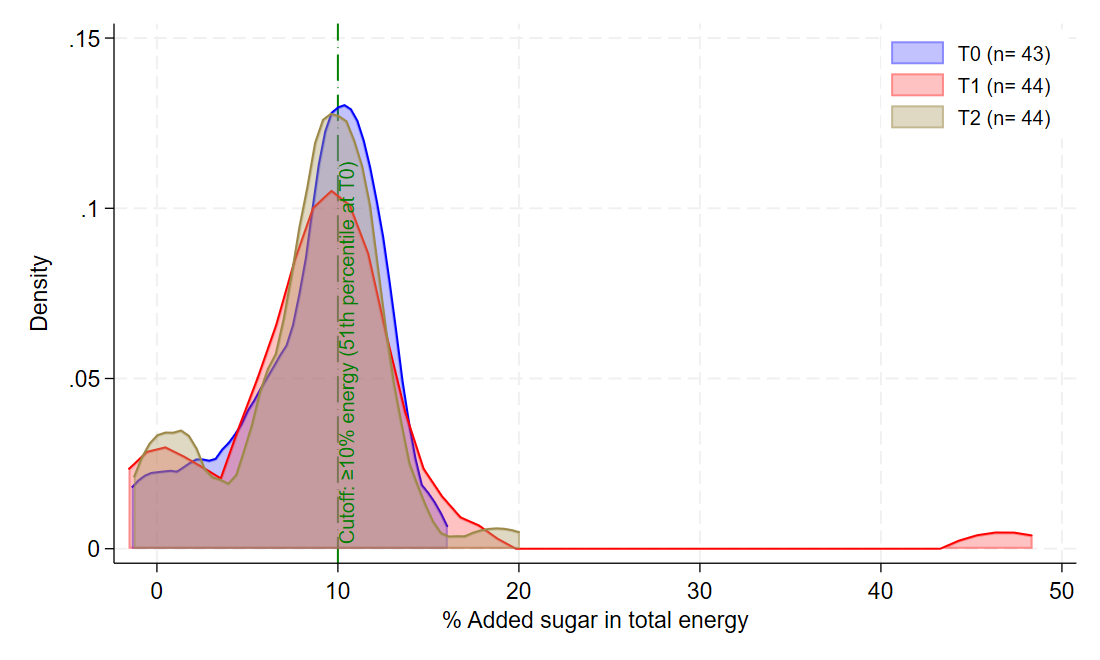 |
| --- | --- |
| **c)**  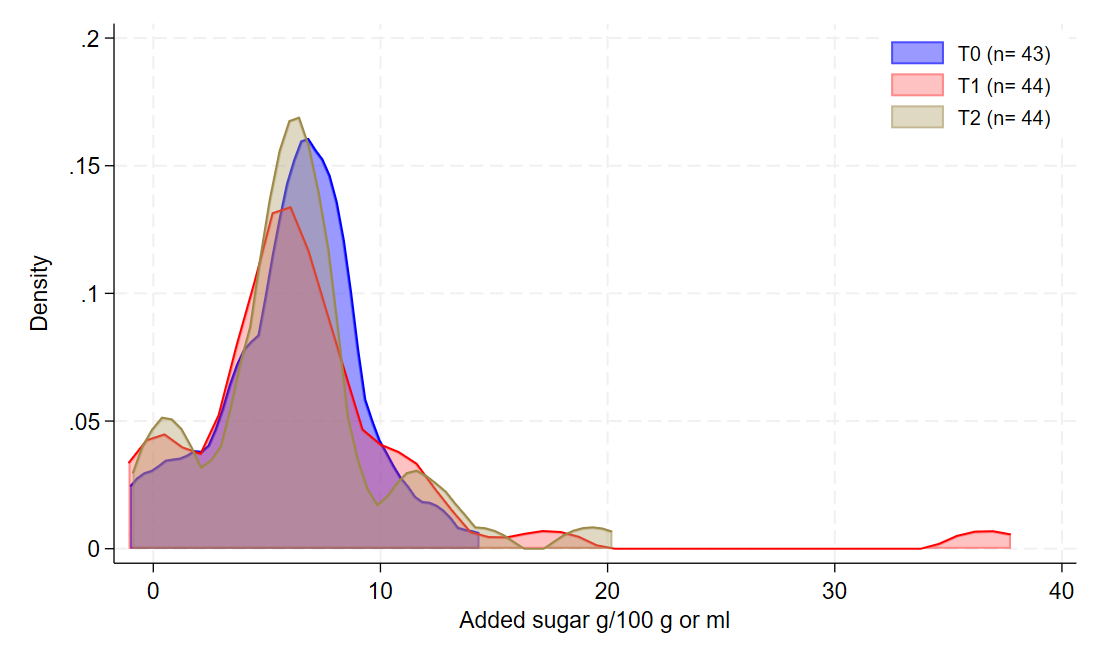 | **d)**  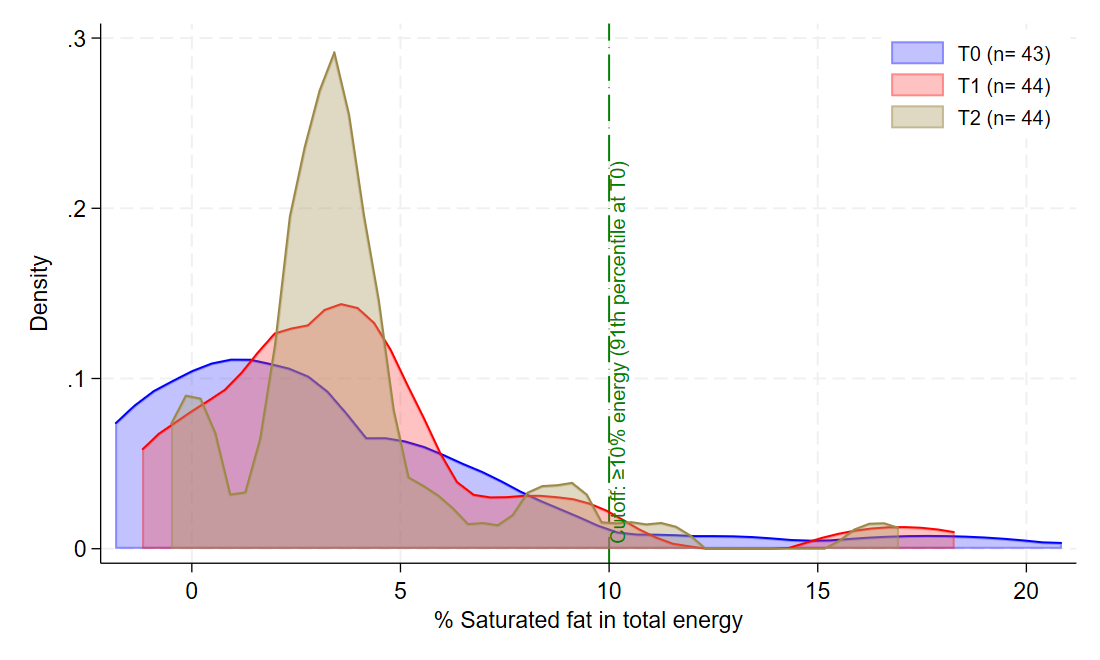 |
| **e)**  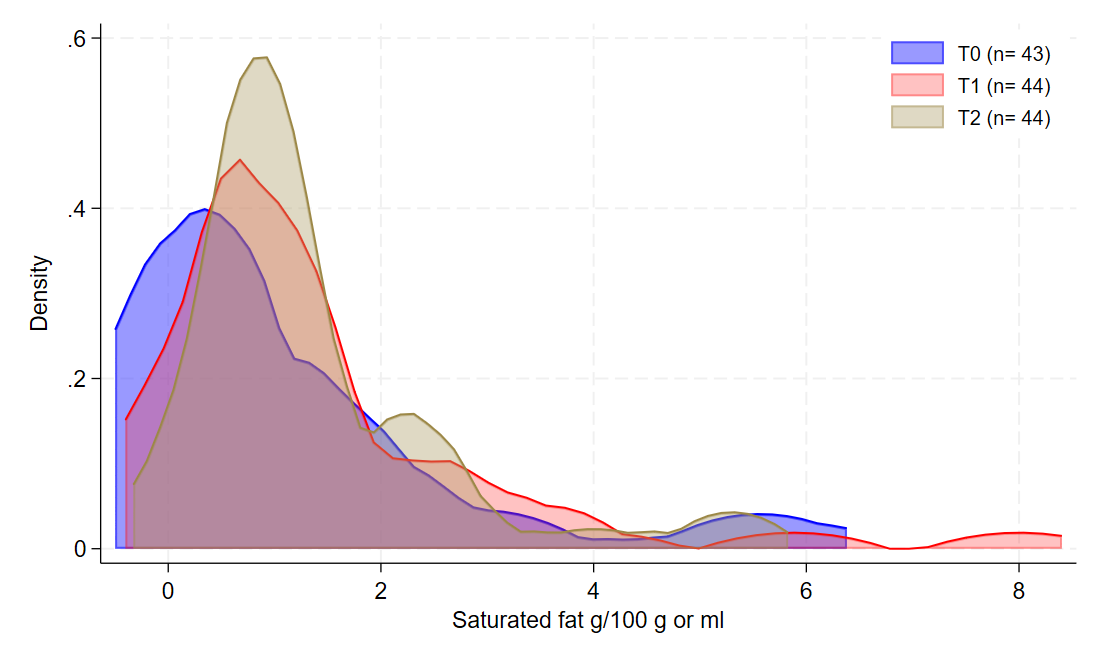 | **f)**  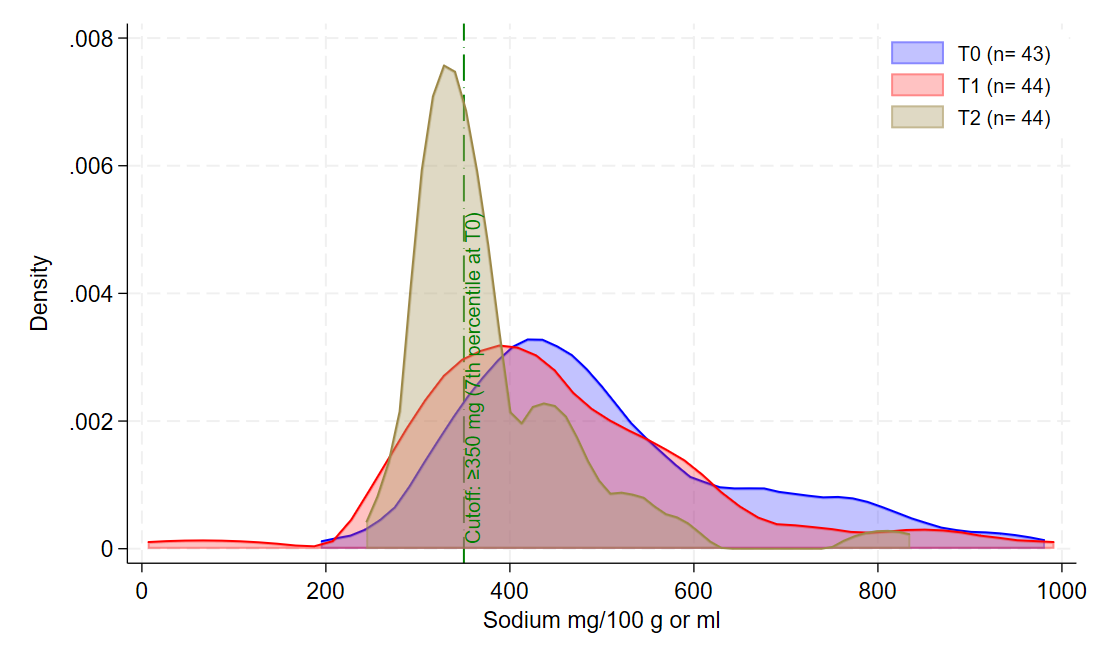 |
| **Note:** a) kcal/100 g or ml, b) Added sugar as % of total energy, c) Added sugar g/100 g or ml, d) Saturated fat as % of total energy, e) Saturated fat g/100 g or ml, f) Sodium mg/100 g or ml. All cutoffs are based on the criteria of the first stage of the front-of-package warning labels, as described in Table 1. | |

## **Figure C.** Kernel densities of calories and critical nutrients for salty snacks

| **a)**  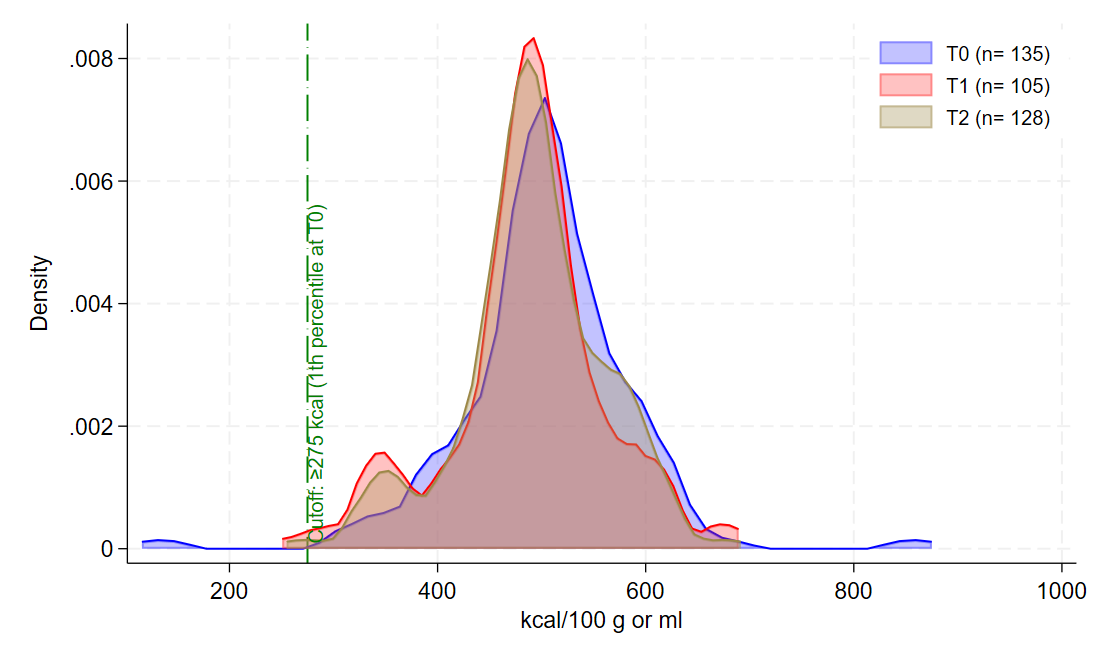 | **b)**  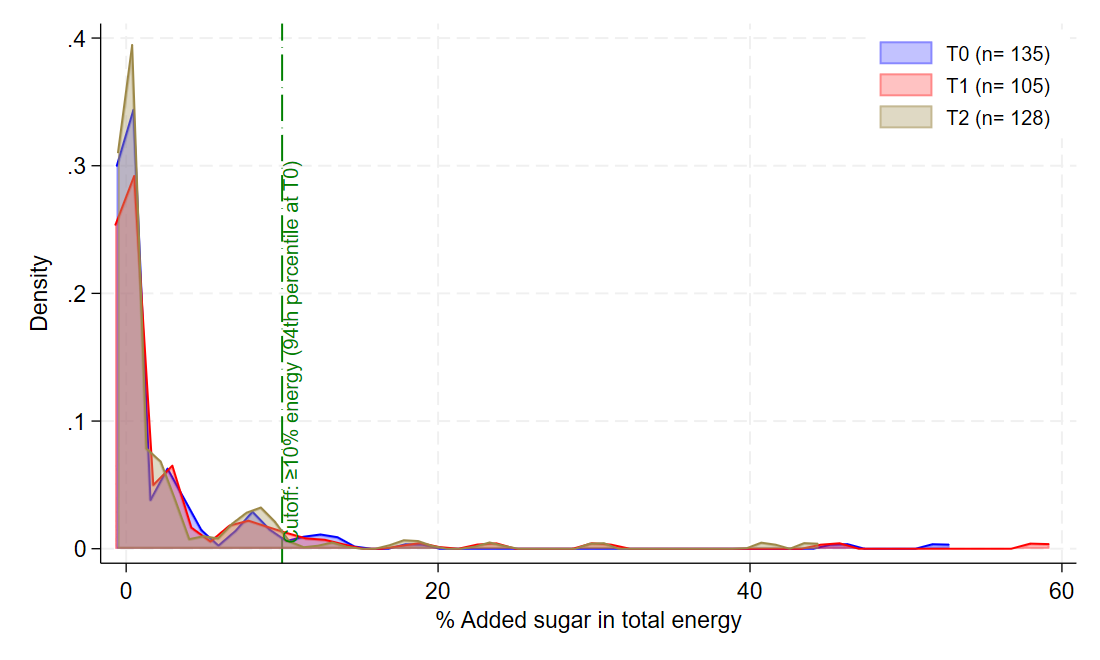 |
| --- | --- |
| **c)**  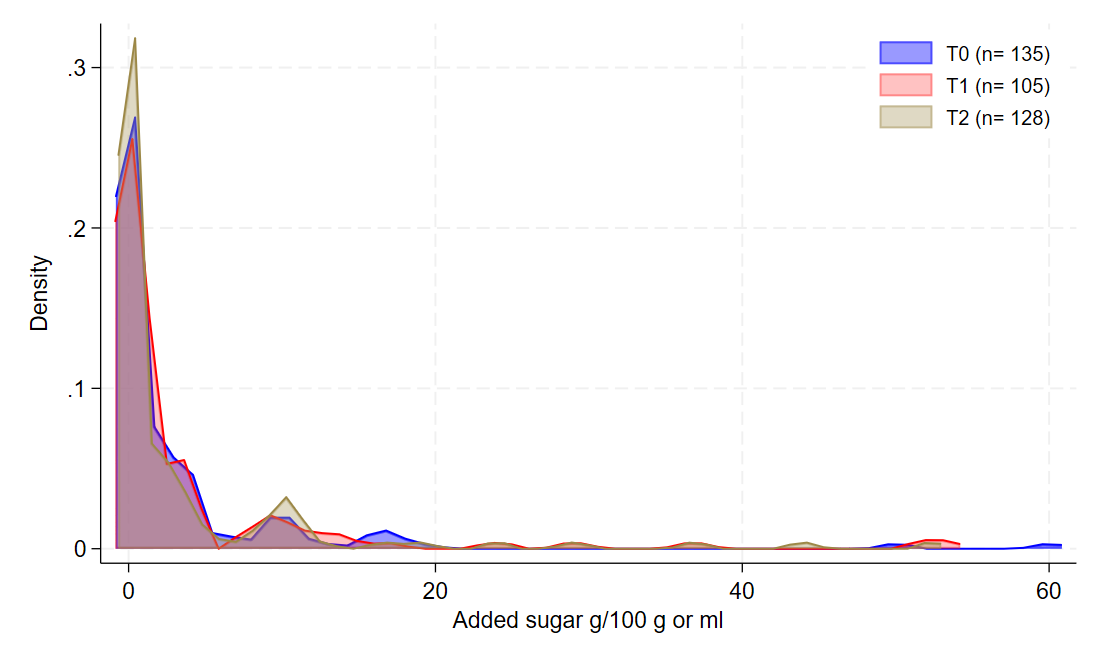 | **d)**  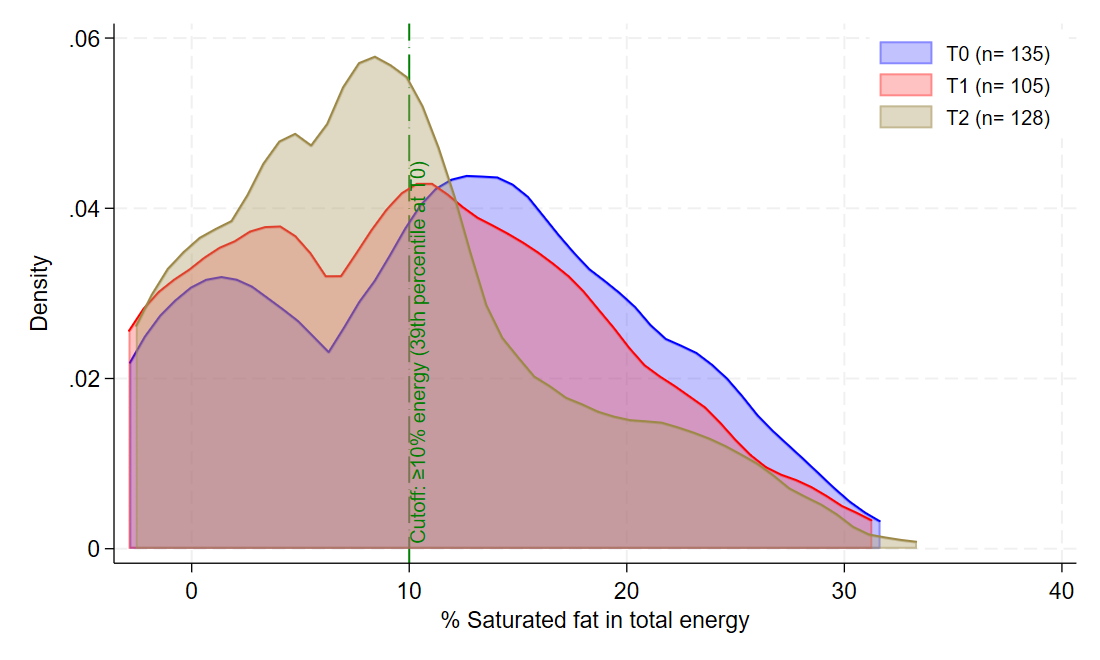 |
| **e)**  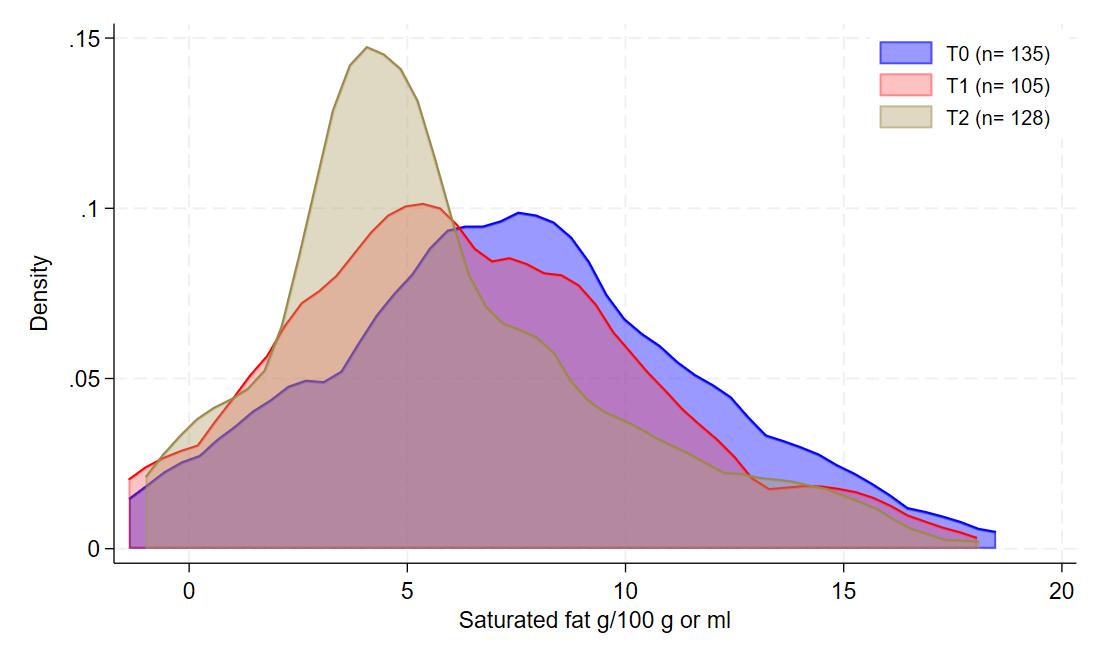 | **f)**  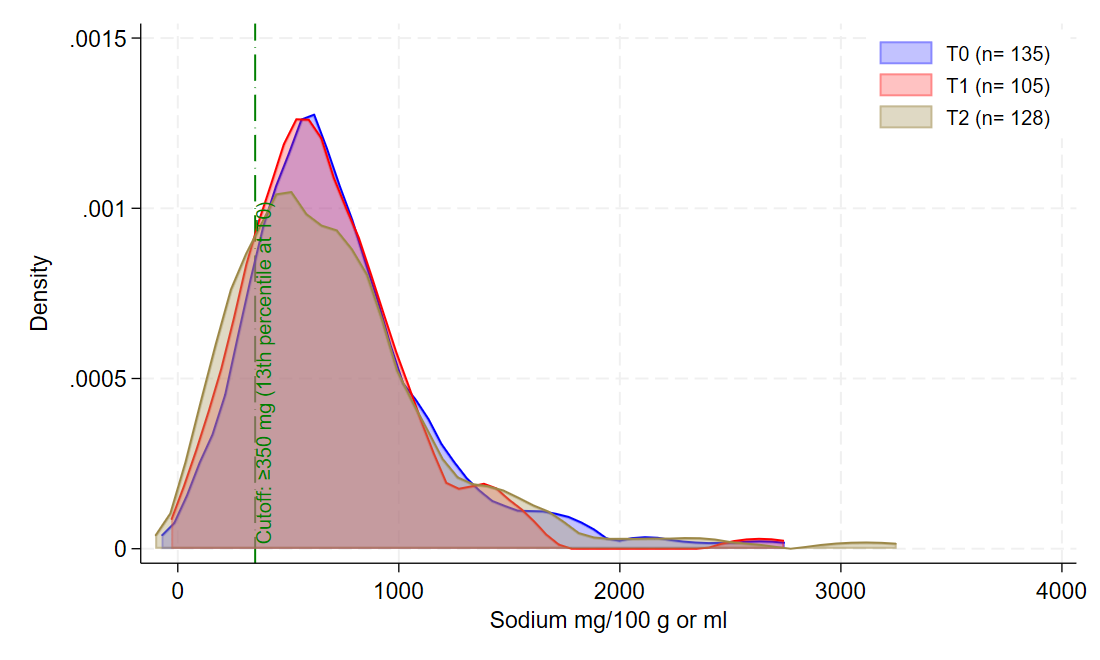 |
| **Note:** a) kcal/100 g or ml, b) Added sugar as % of total energy, c) Added sugar g/100 g or ml, d) Saturated fat as % of total energy, e) Saturated fat g/100 g or ml, f) Sodium mg/100 g or ml. All cutoffs are based on the criteria of the first stage of the front-of-package warning labels, as described in Table 1. | |

## **Figure D.** Kernel densities of calories and critical nutrients for sweetened beverages

| **a)**  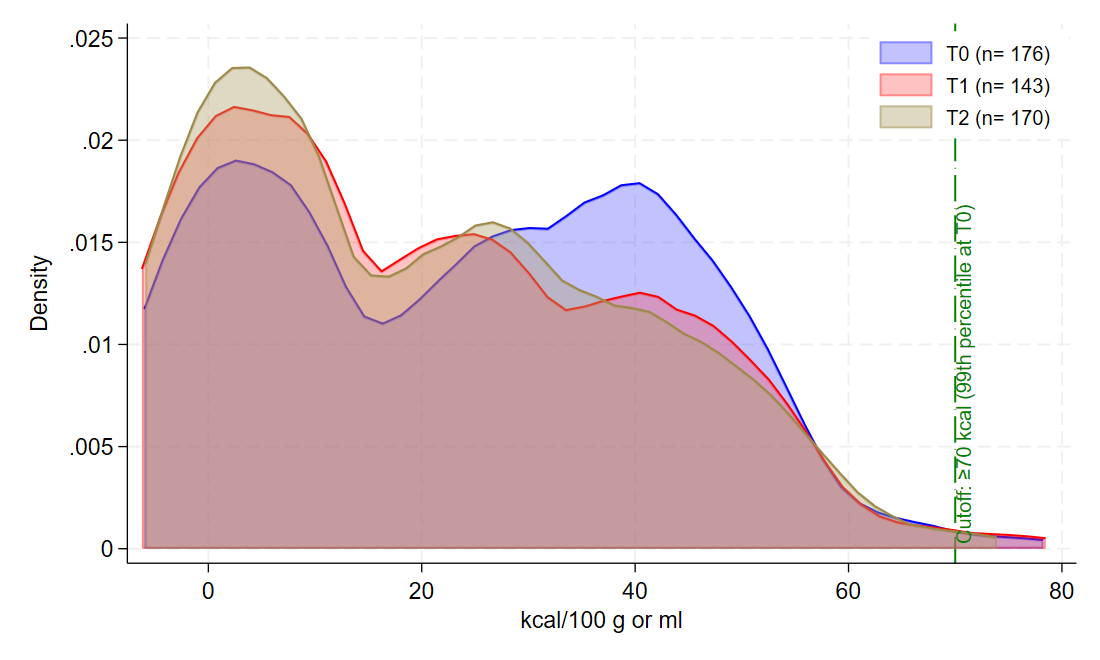 | **b)**  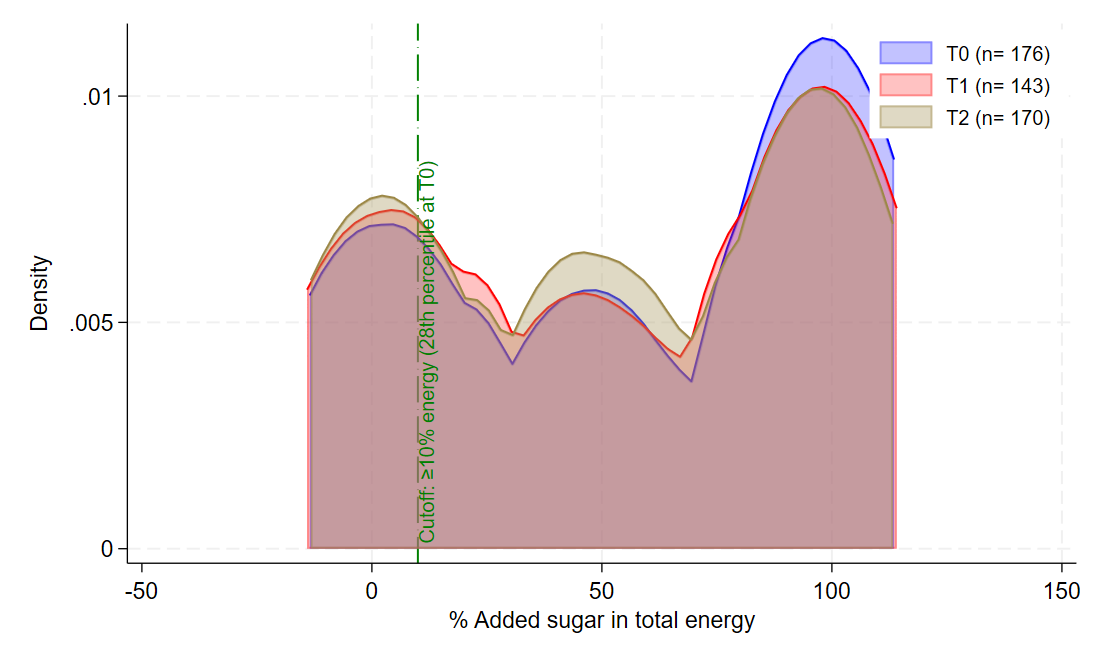 |
| --- | --- |
| **c)**  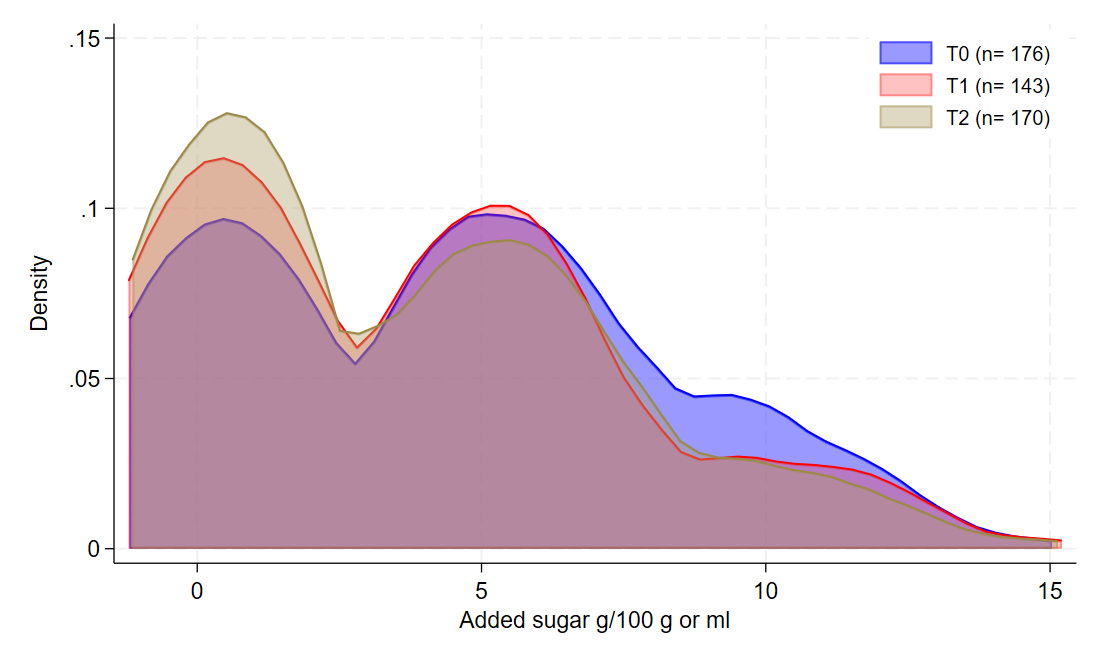 | **d)**  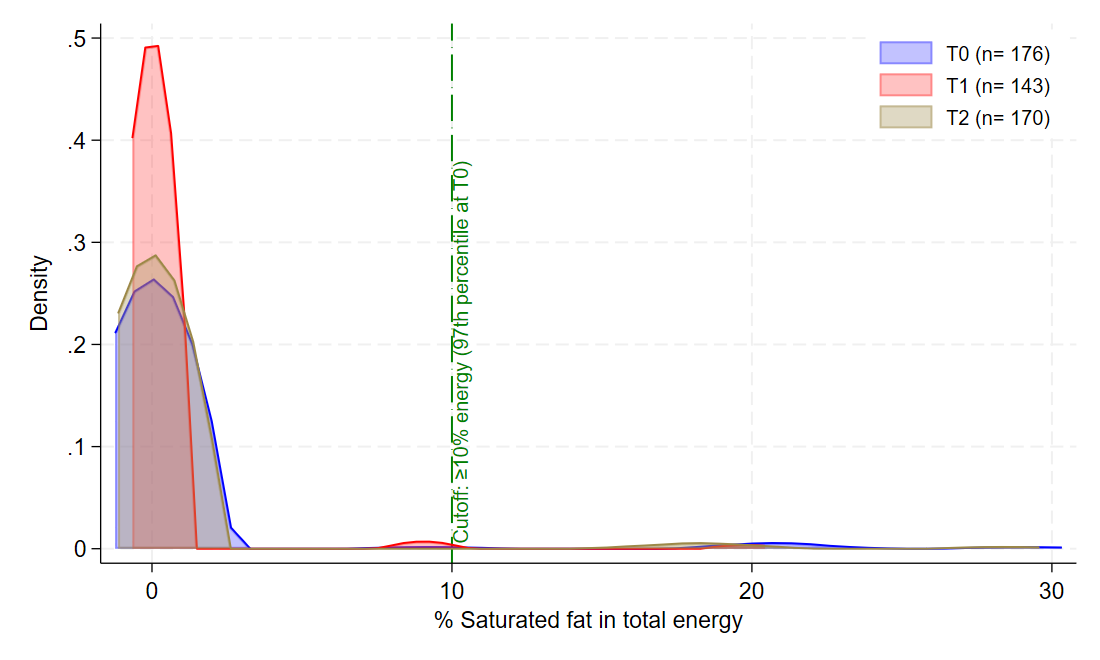 |
| **e)**  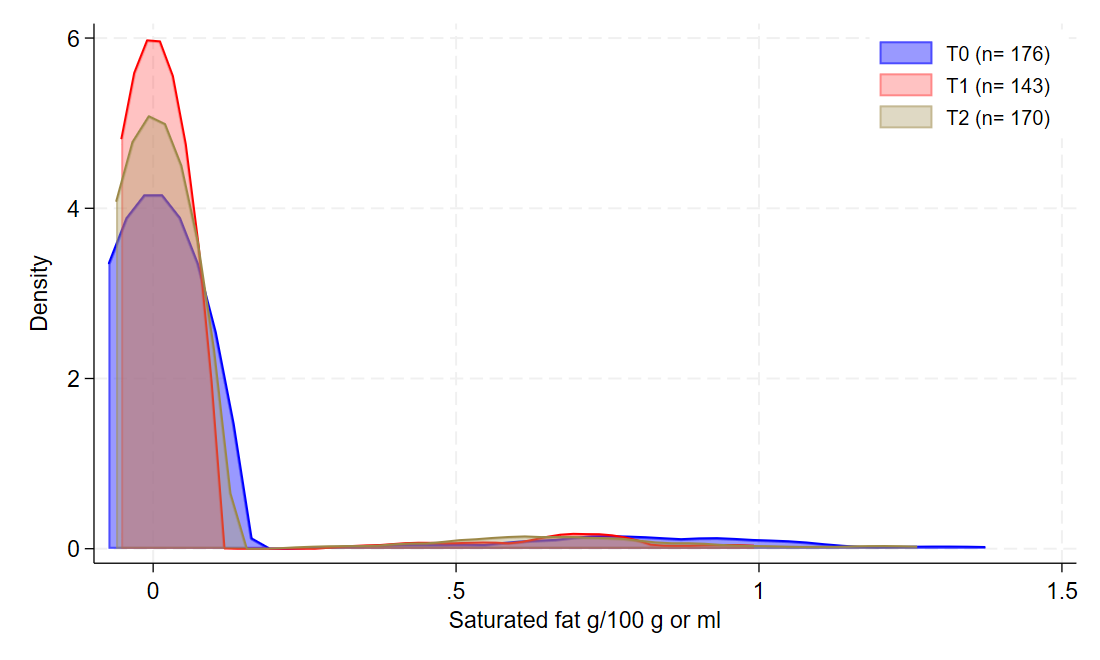 | **f)**  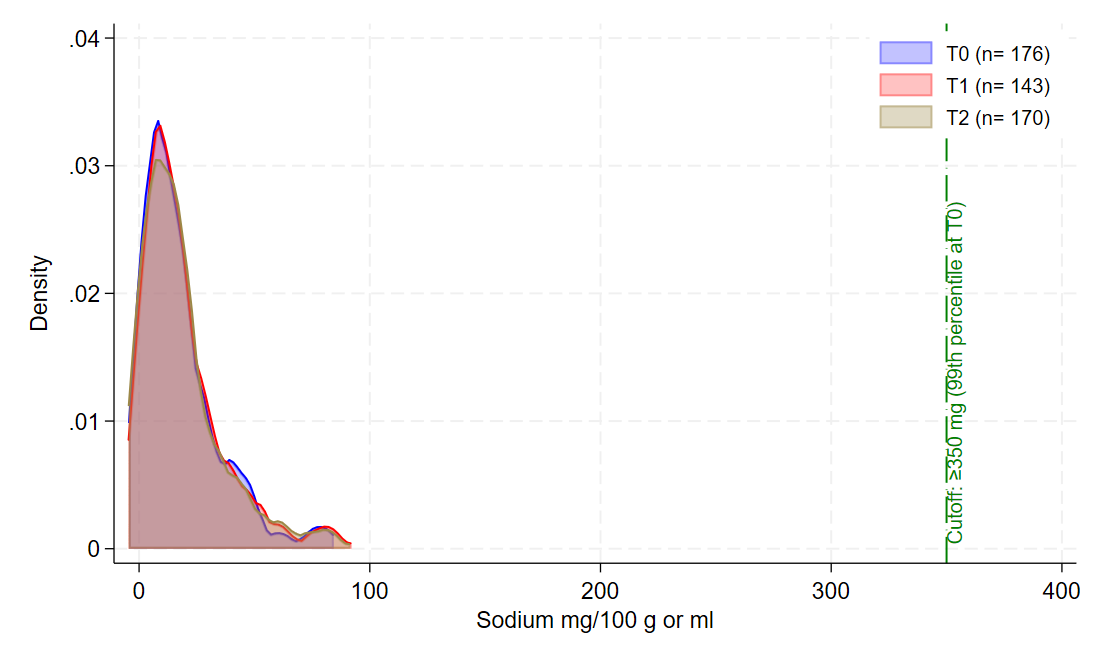 |
| **Note:** a) kcal/100 g or ml, b) Added sugar as % of total energy, c) Added sugar g/100 g or ml, d) Saturated fat as % of total energy, e) Saturated fat g/100 g or ml, f) Sodium mg/100 g or ml. As sugar-sweetened beverages are measured in milliliters in our analytical data (i.e., liquid products), in addition to the cutoffs in panels a), b) and f), the next criteria are also applied: when ≥ 10 calories come from free sugar, the product has to display the warning label of "excess calories"; when less than 10 calories come from free sugar, the product is exempted from displaying the warning label of "excess sugar"; when ≥ 45 mg sodium/ 100ml and the product has no calories, the product has to display the warning label of "excess sodium". All cutoffs are based on the criteria of the first stage of the front-of-package warning labels, as described in Table 1. | |

## **Figure E.** Kernel densities of calories and critical nutrients for liquid dairy

| **a)**  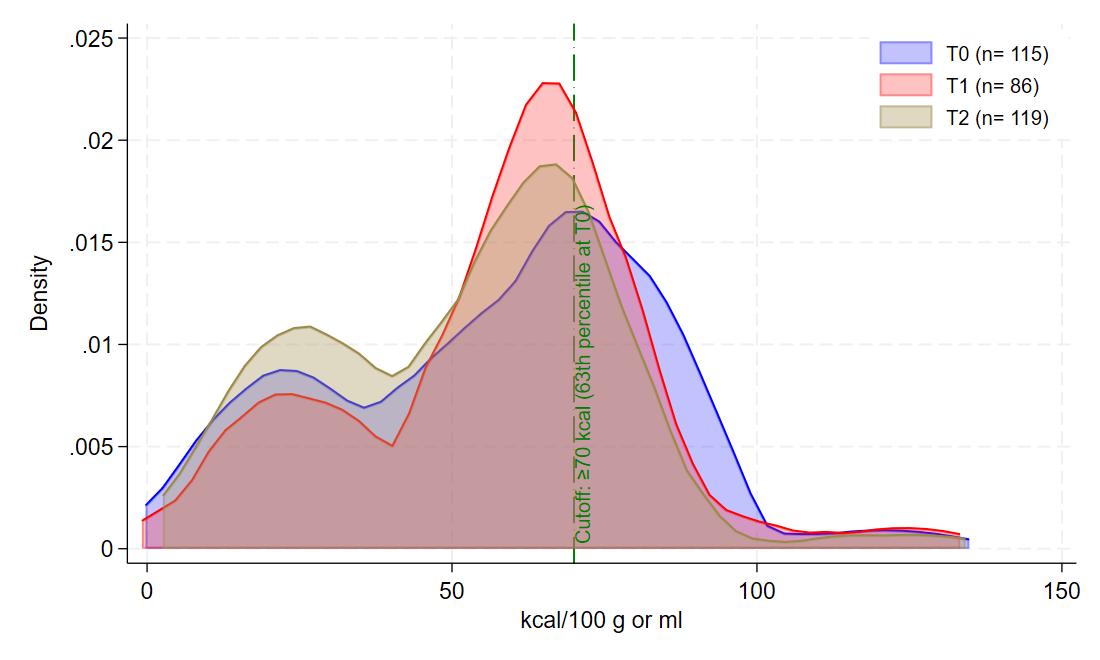 | **b)** 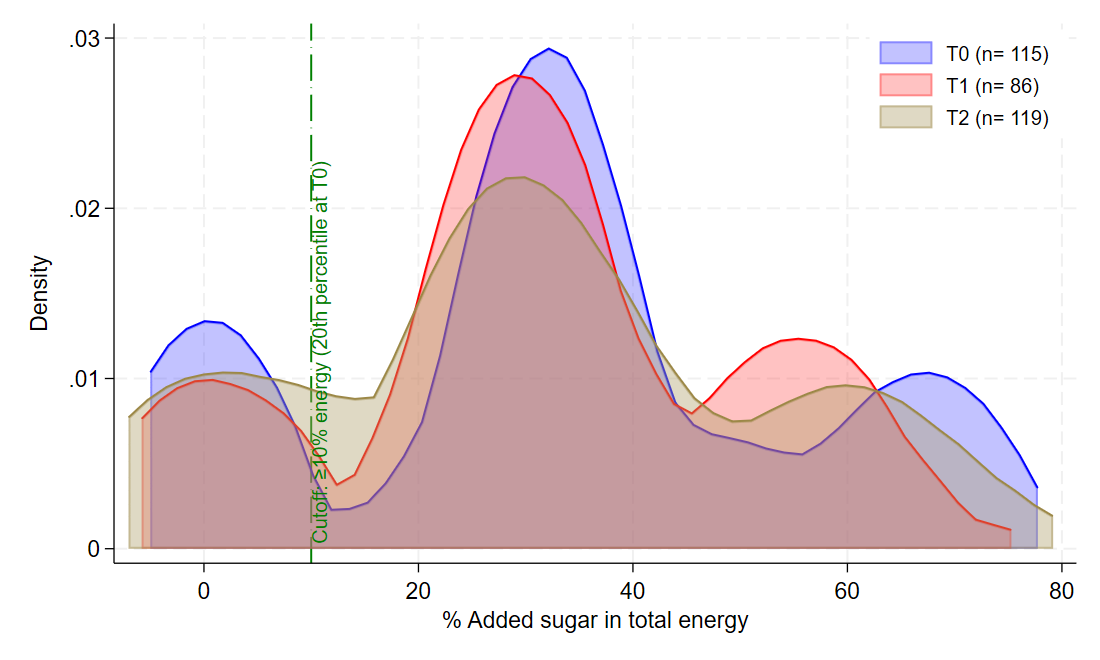 |
| --- | --- |
| **c)**  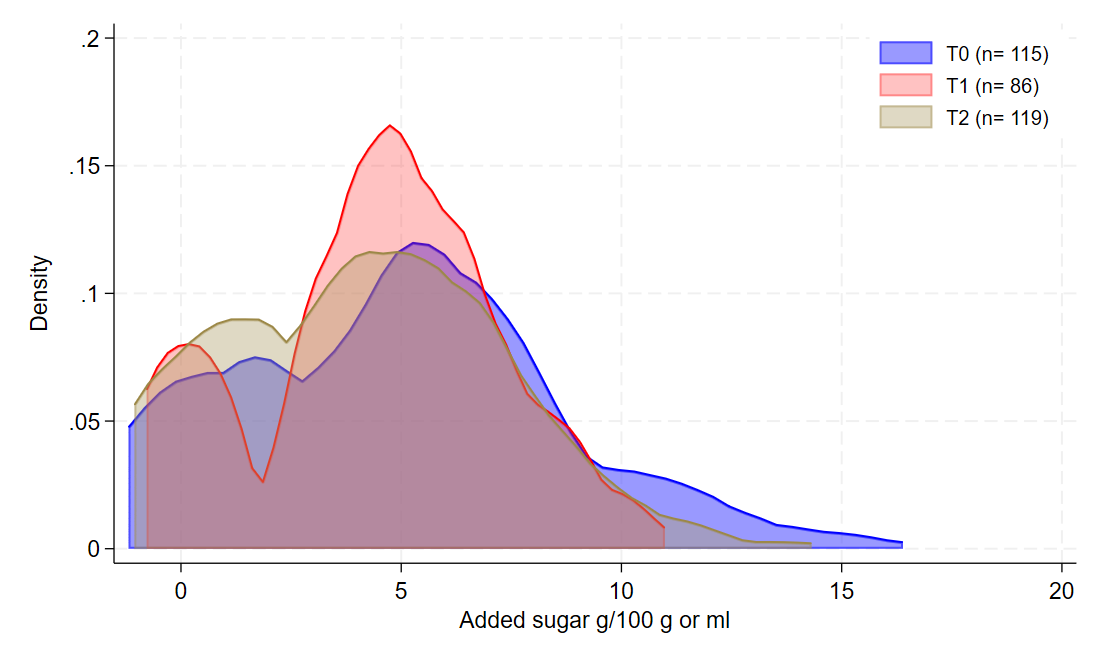 | **d)**  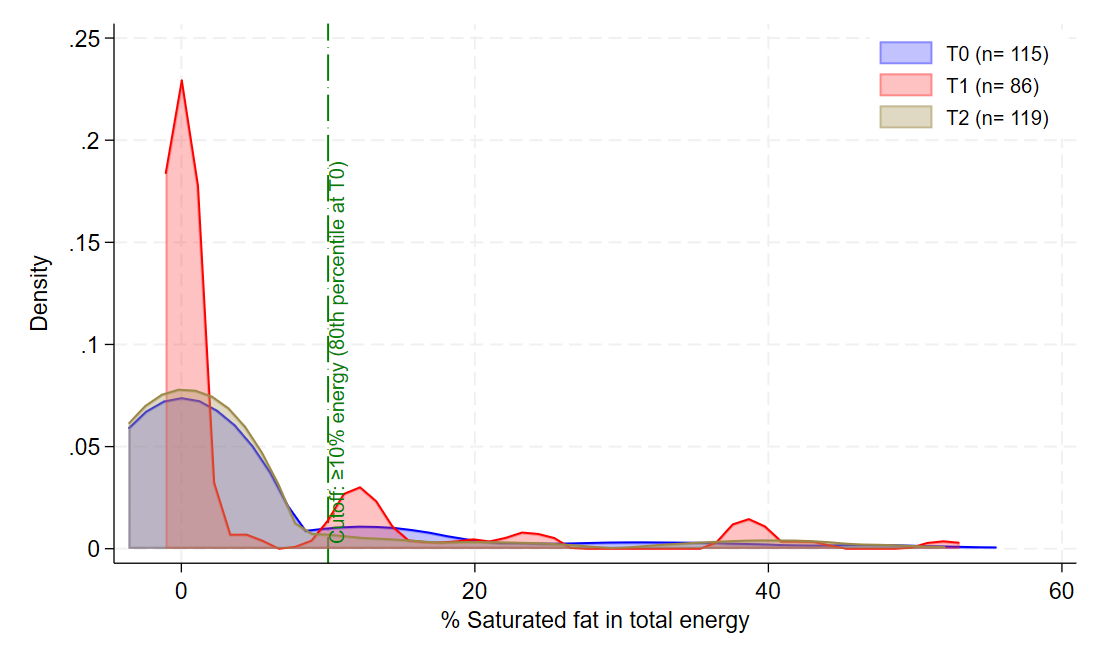 |
| **e)**  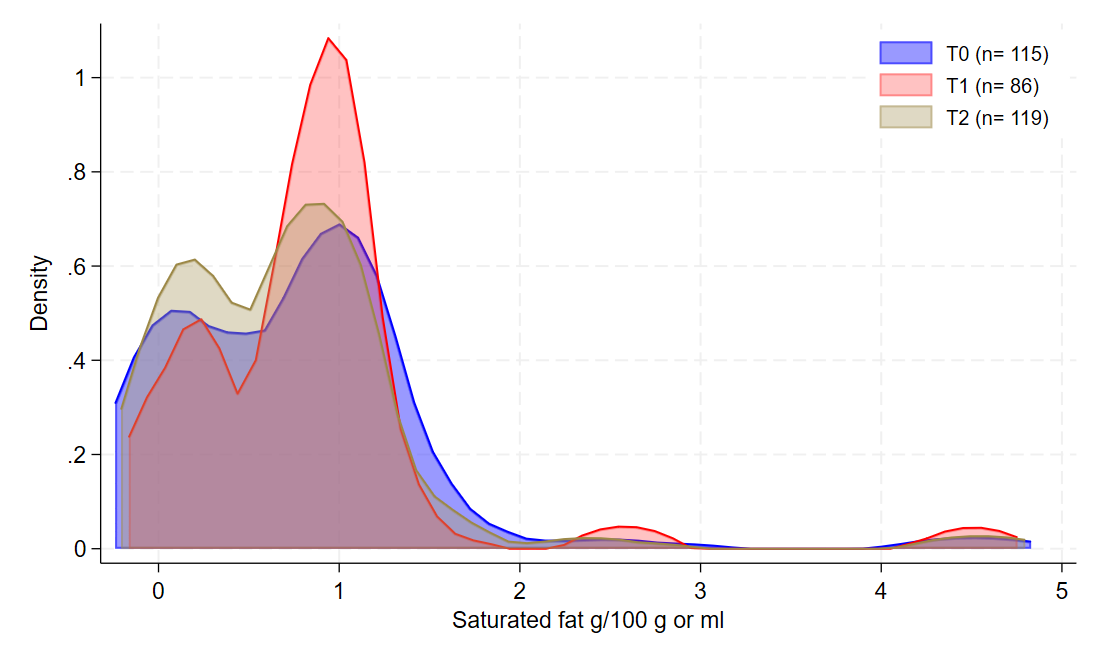 | **f)**  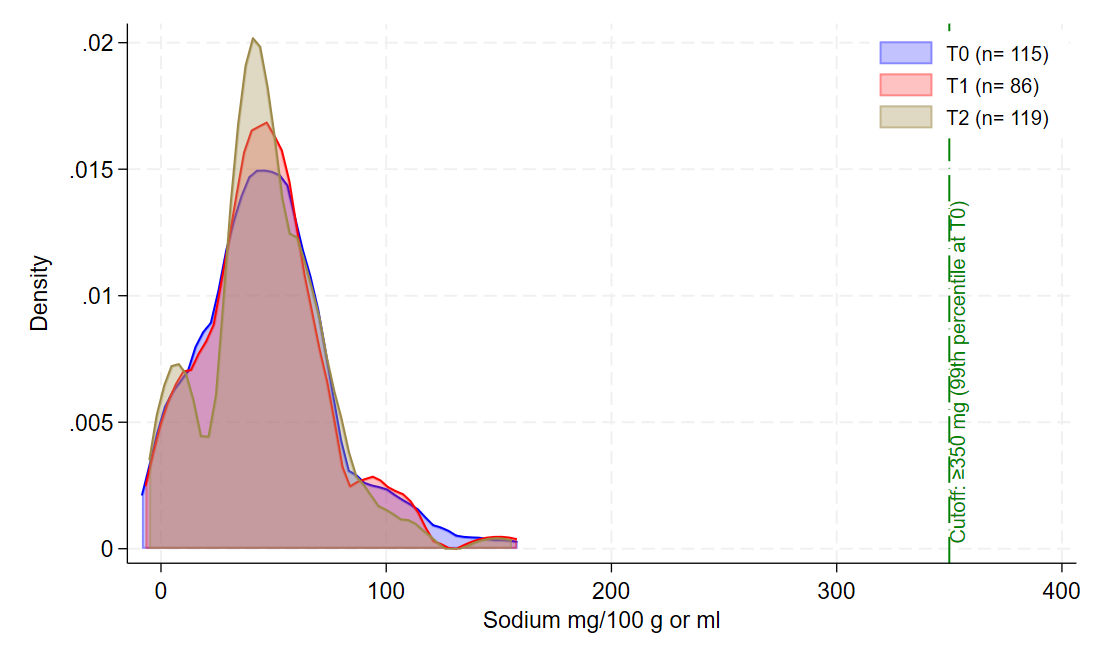 |
| **Note:** a) kcal/100 g or ml, b) Added sugar as % of total energy, c) Added sugar g/100 g or ml, d) Saturated fat as % of total energy, e) Saturated fat g/100 g or ml, f) Sodium mg/100 g or ml. As liquid dairy are measured in milliliters in our analytical data (i.e., liquid products), in addition to the cutoffs in panels a), b) and f), the next criteria are also applied: when ≥ 10 calories come from free sugar, the product has to display the warning label of “excess calories”; when less than 10 calories come from free sugar, the product is exempted from displaying the warning label of “excess sugar”; when ≥ 45 mg sodium/ 100ml and the product has no calories, the product has to display the warning label of “excess sodium”. All cutoffs are based on the criteria of the first stage of the front-of-package warning labels, as described in Table 1. | |

## **Figure F.** Kernel densities of calories and critical nutrients for solid dairy

| **a)**  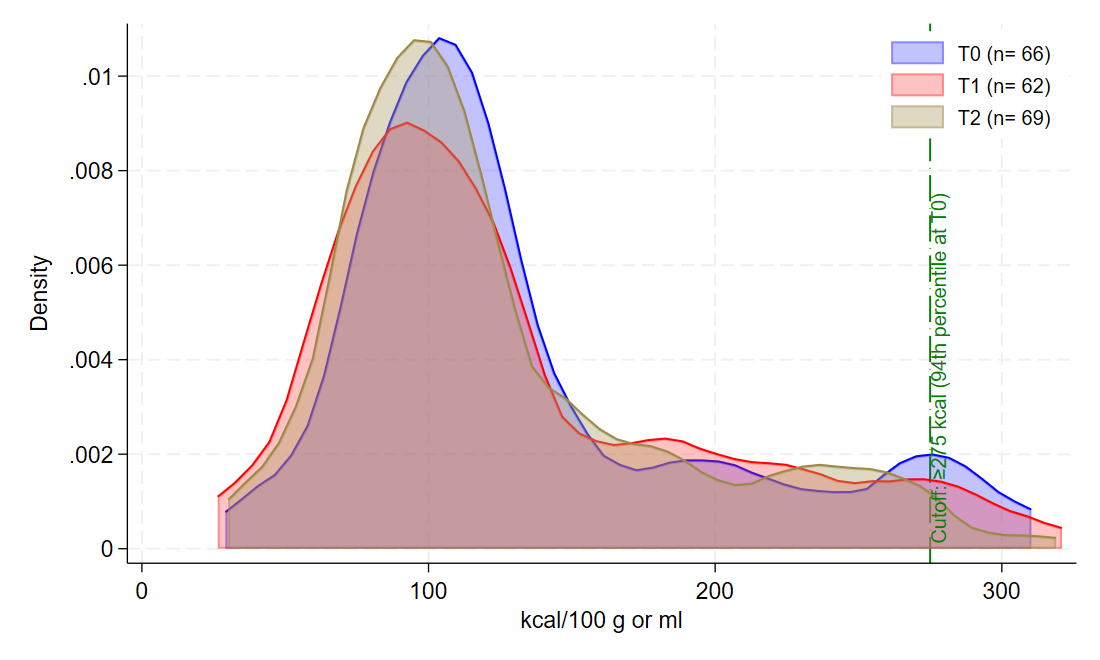 | **b)**  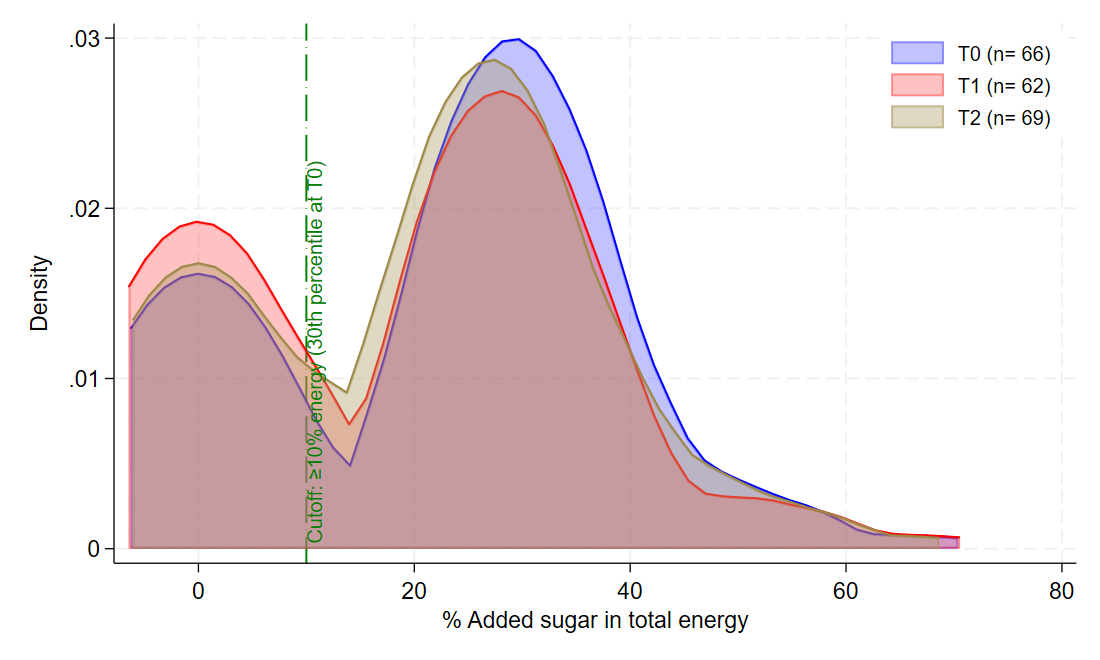 |
| --- | --- |
| **c)**  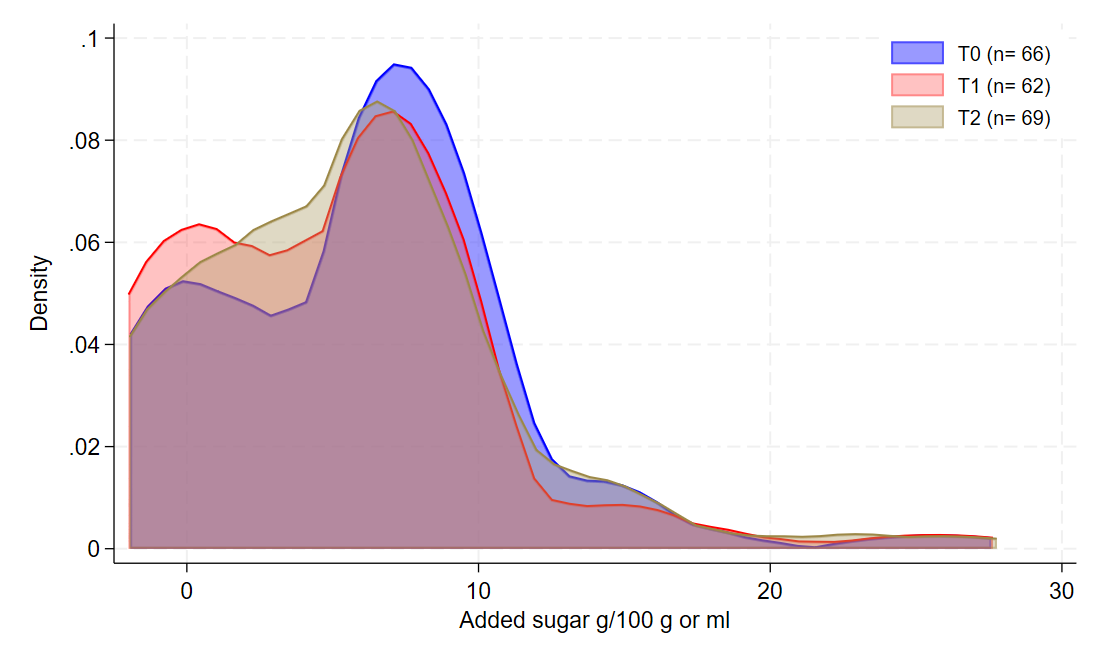 | **d)**  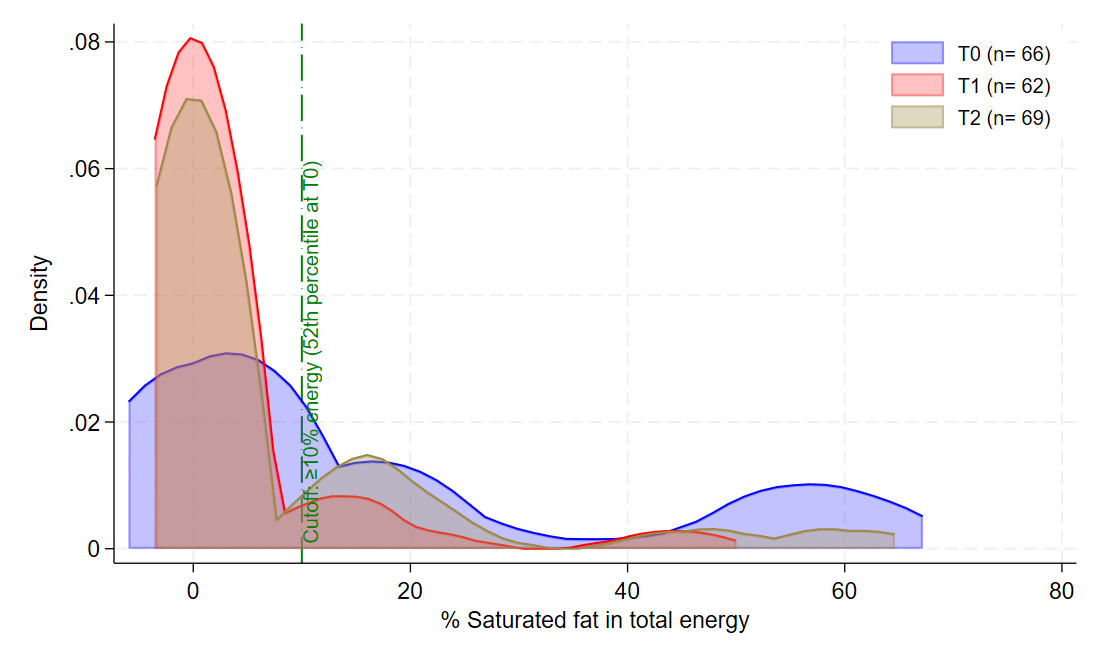 |
| **e)**  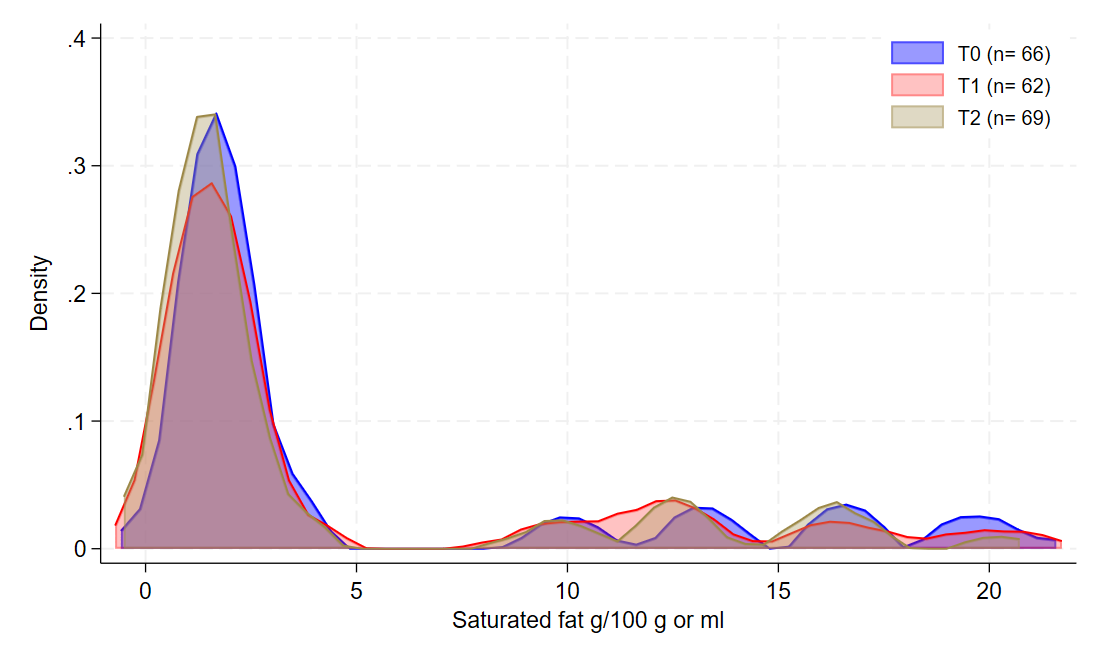 | **f)**  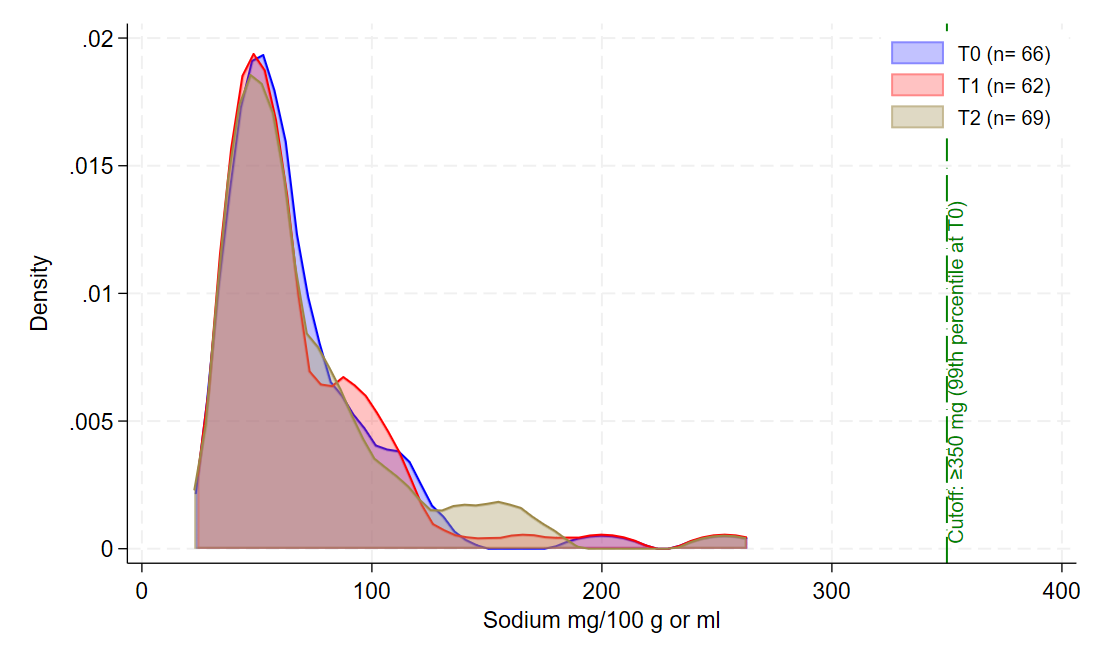 |
| **Note:** a) kcal/100 g or ml, b) Added sugar as % of total energy, c) Added sugar g/100 g or ml, d) Saturated fat as % of total energy, e) Saturated fat g/100 g or ml, f) Sodium mg/100 g or ml. All cutoffs are based on the criteria of the first stage of the front-of-package warning labels, as described in Table 1. | |

## **Figure G.** Kernel densities of calories and critical nutrients for instant food

| **a)**  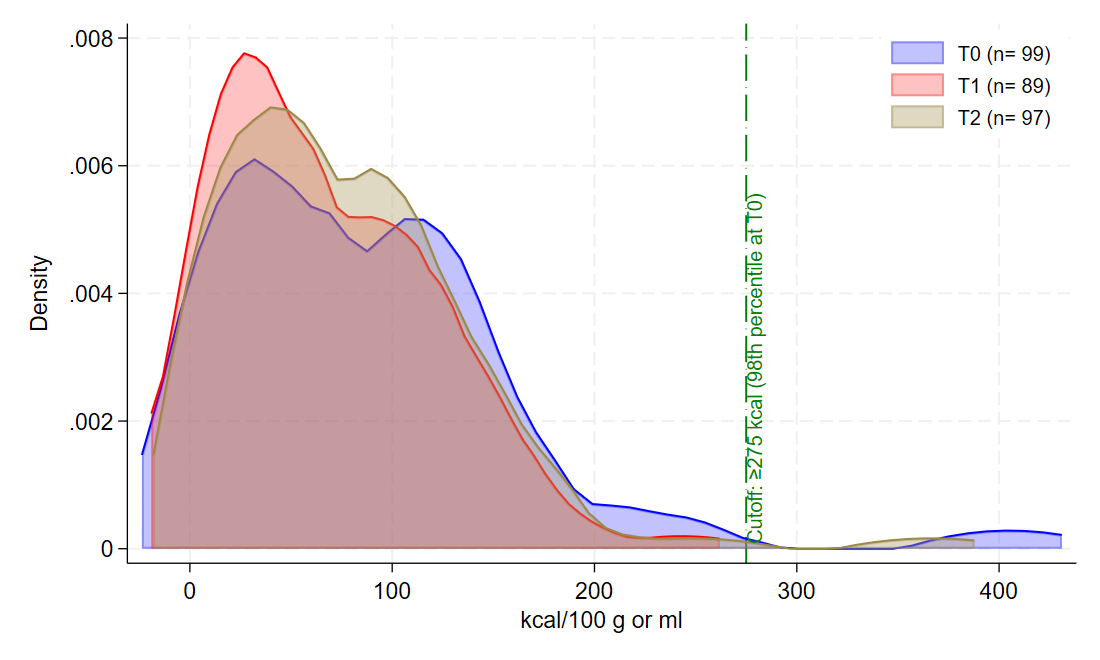 | **b)**  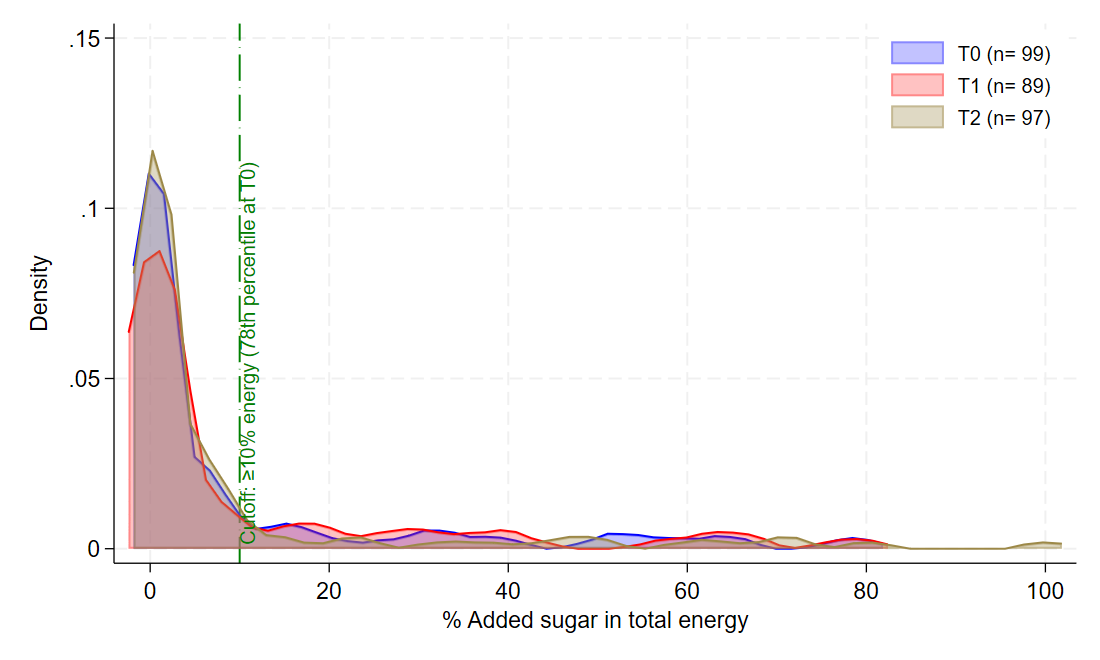 |
| --- | --- |
| **c)**  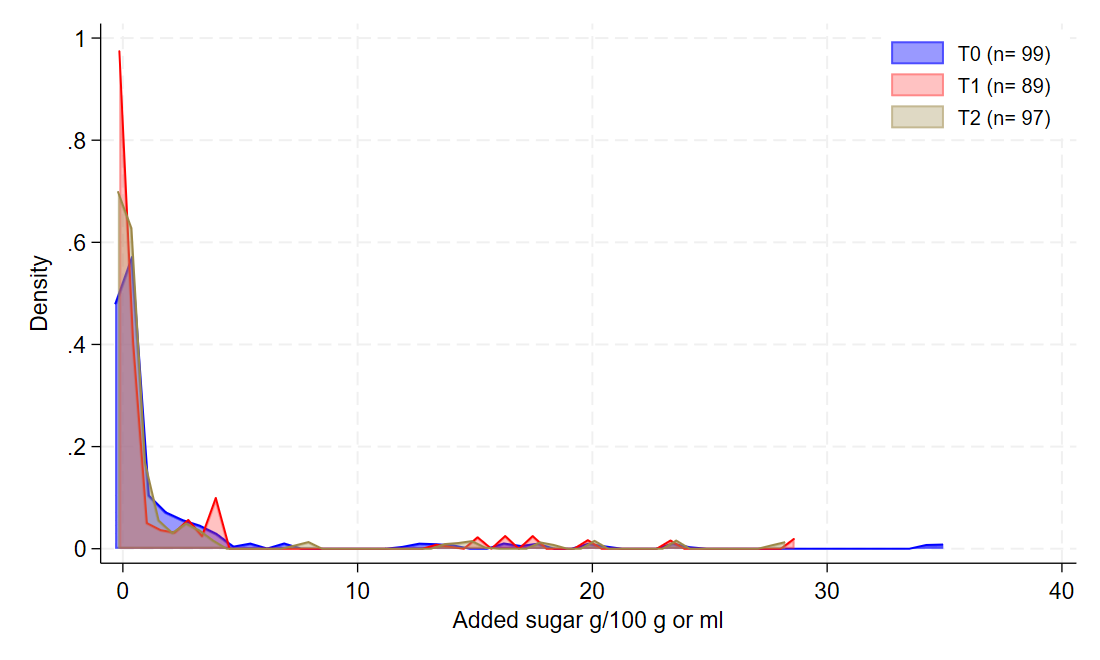 | **d)**  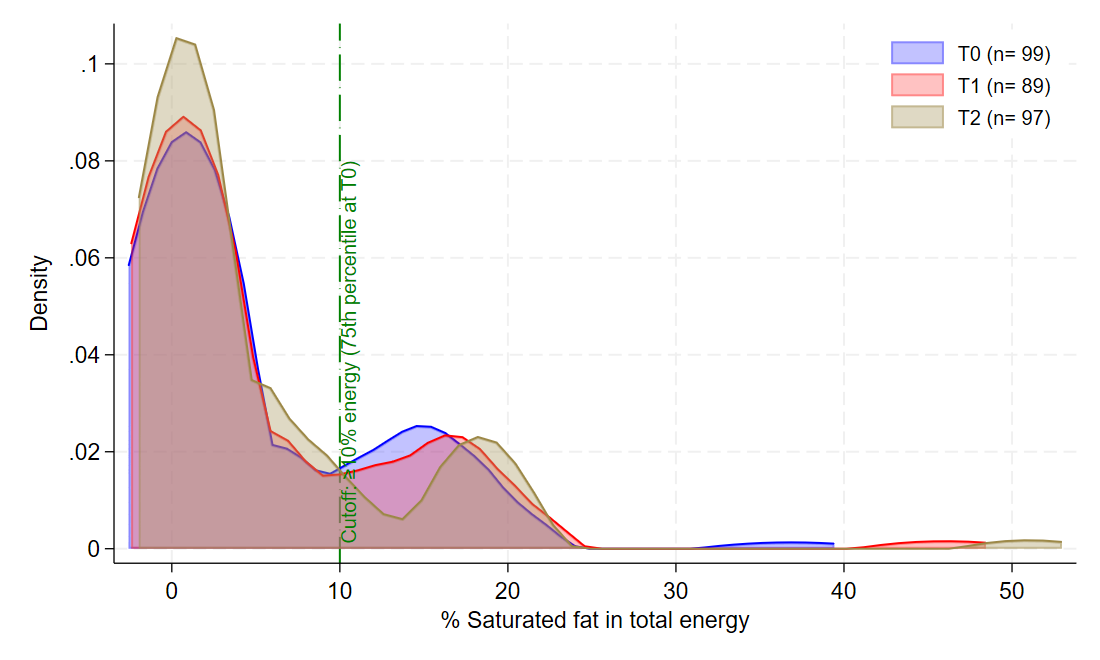 |
| **e)**  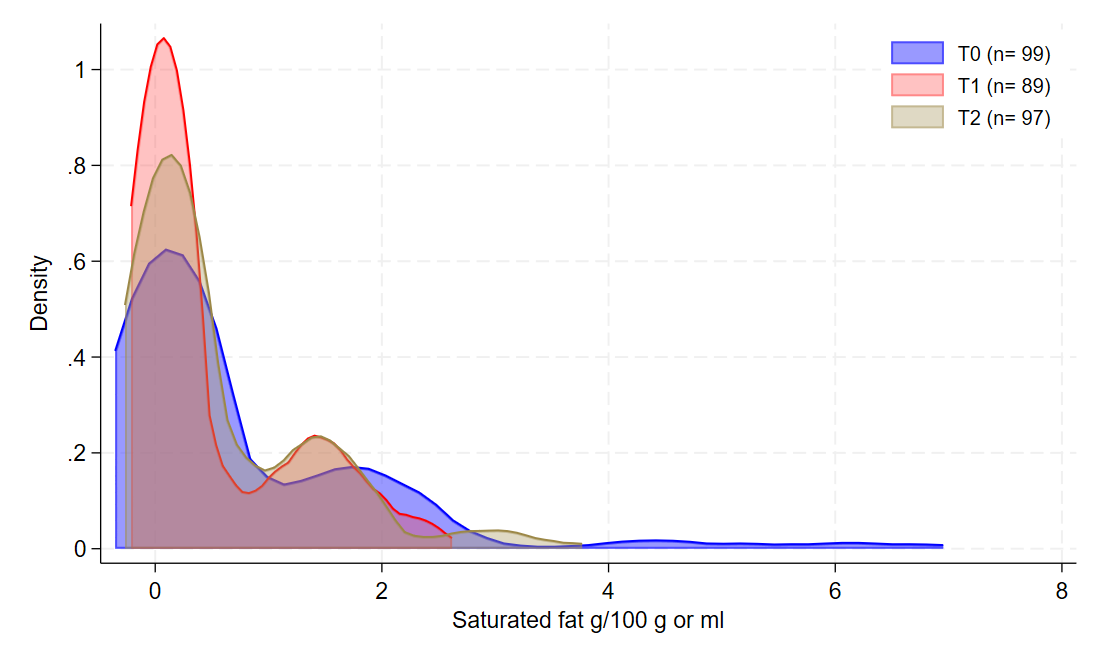 | **f)**  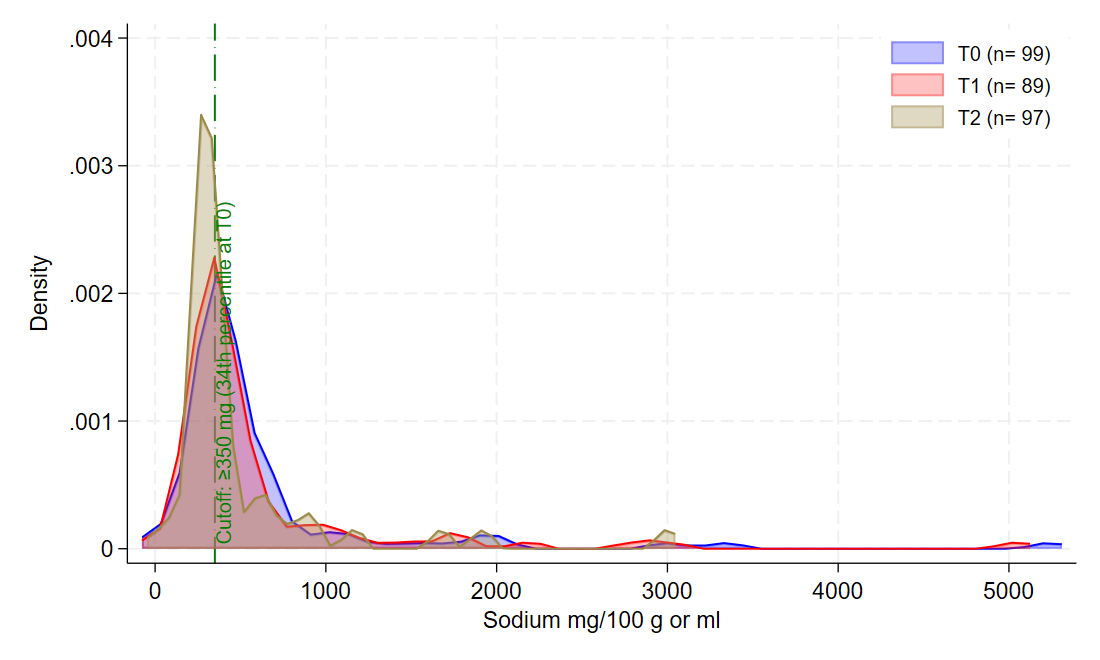 |
| **Note:** a) kcal/100 g or ml, b) Added sugar as % of total energy, c) Added sugar g/100 g or ml, d) Saturated fat as % of total energy, e) Saturated fat g/100 g or ml, f) Sodium mg/100 g or ml. All cutoffs are based on the criteria of the first stage of the front-of-package warning labels, as described in Table 1. | |

## **Figure H.** Kernel densities of calories and critical nutrients for candies

| **a)**  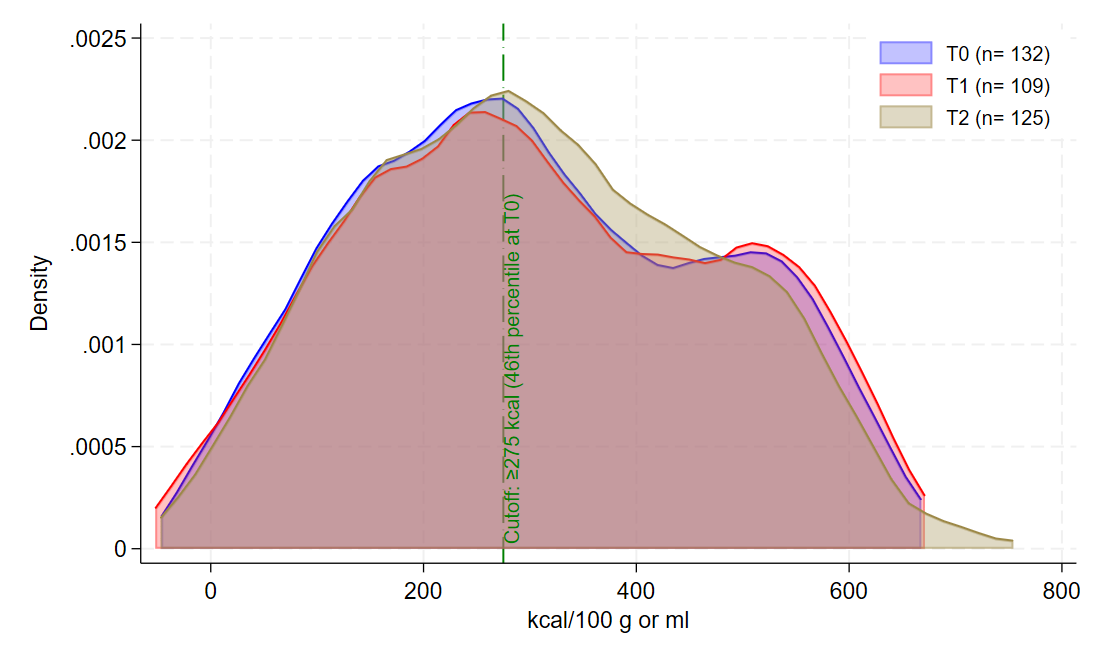 | **b)**  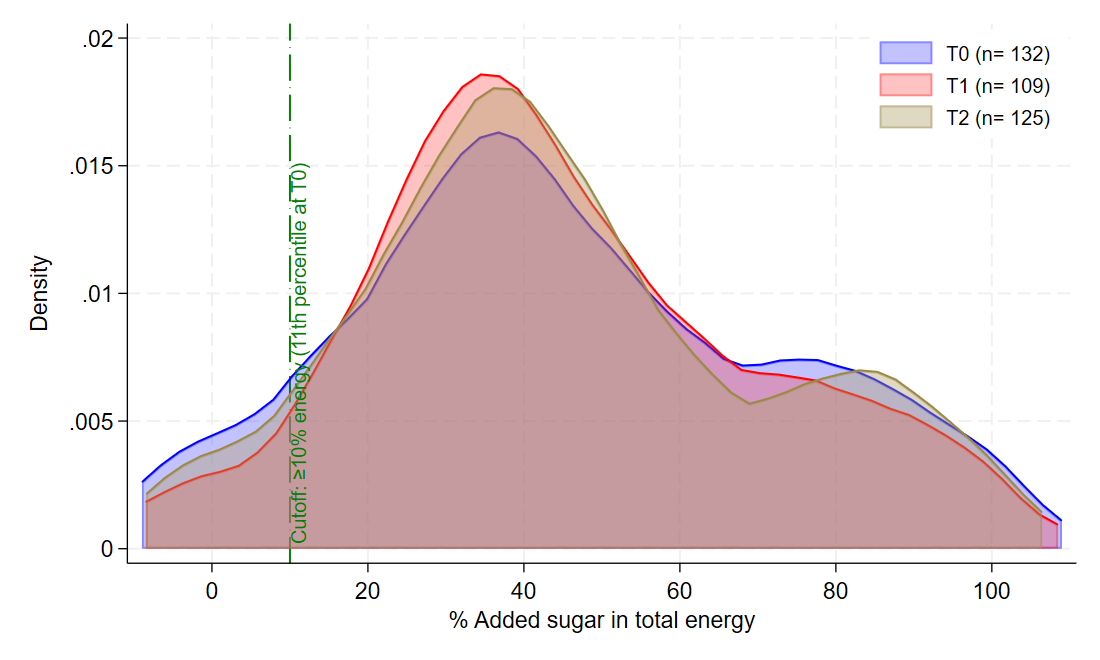 |
| --- | --- |
| **c)**  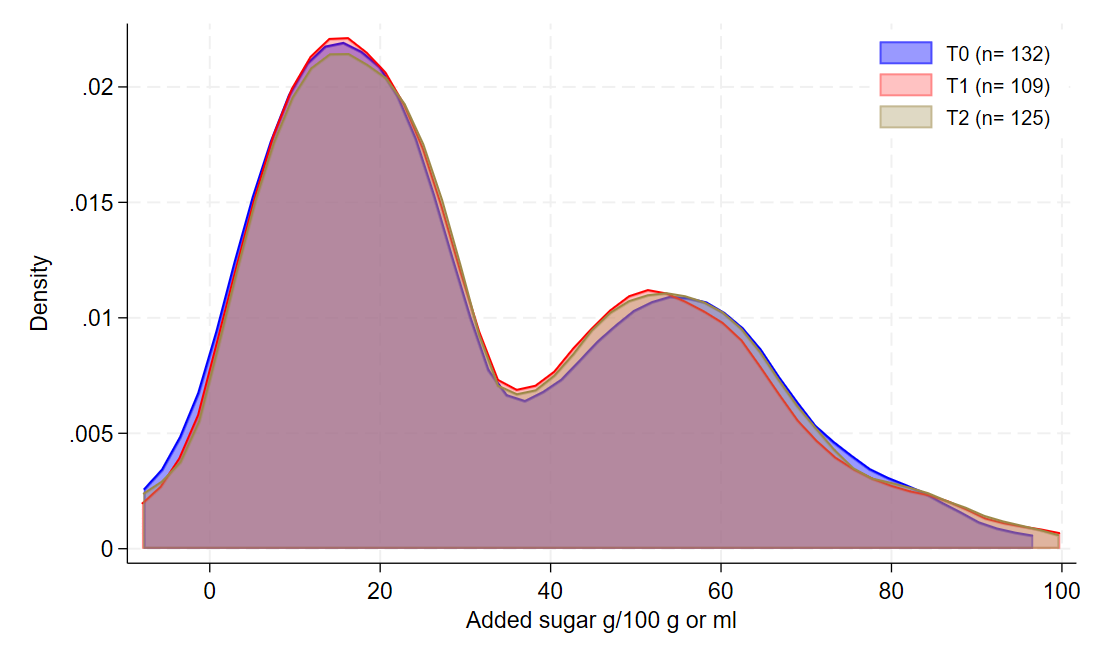 | **d)**  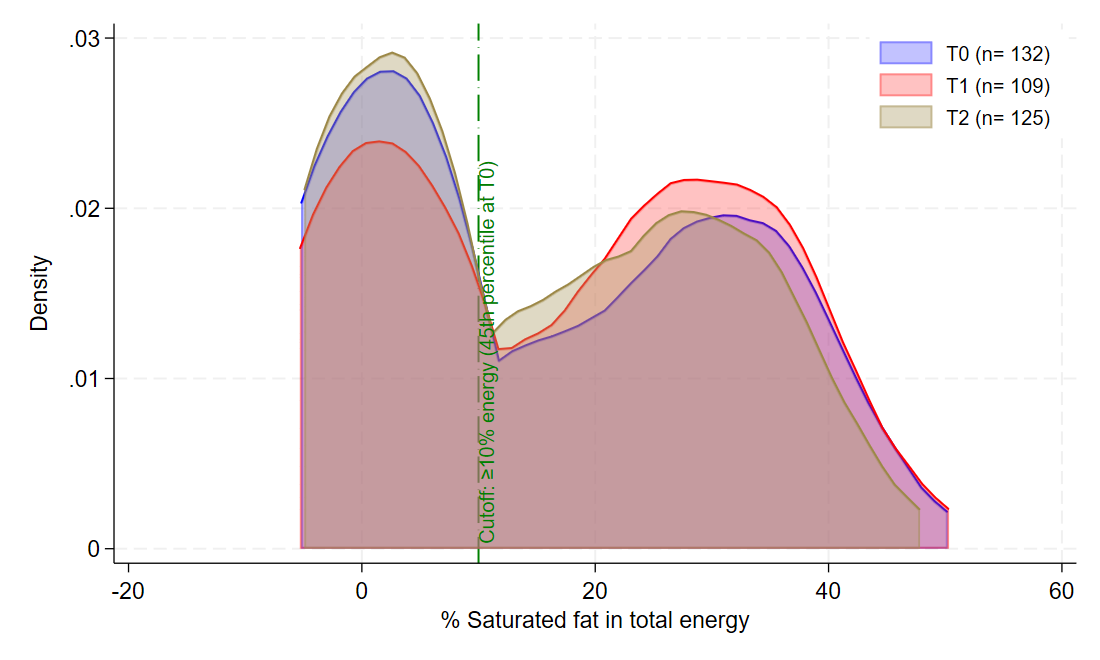 |
| **e)**  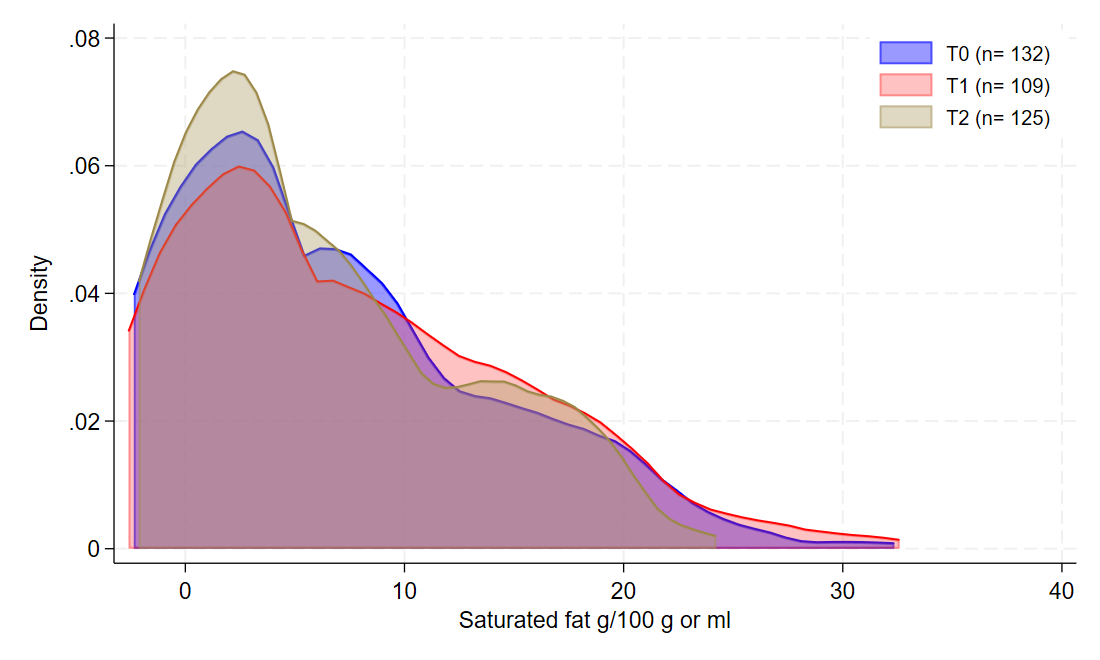 | **f)**  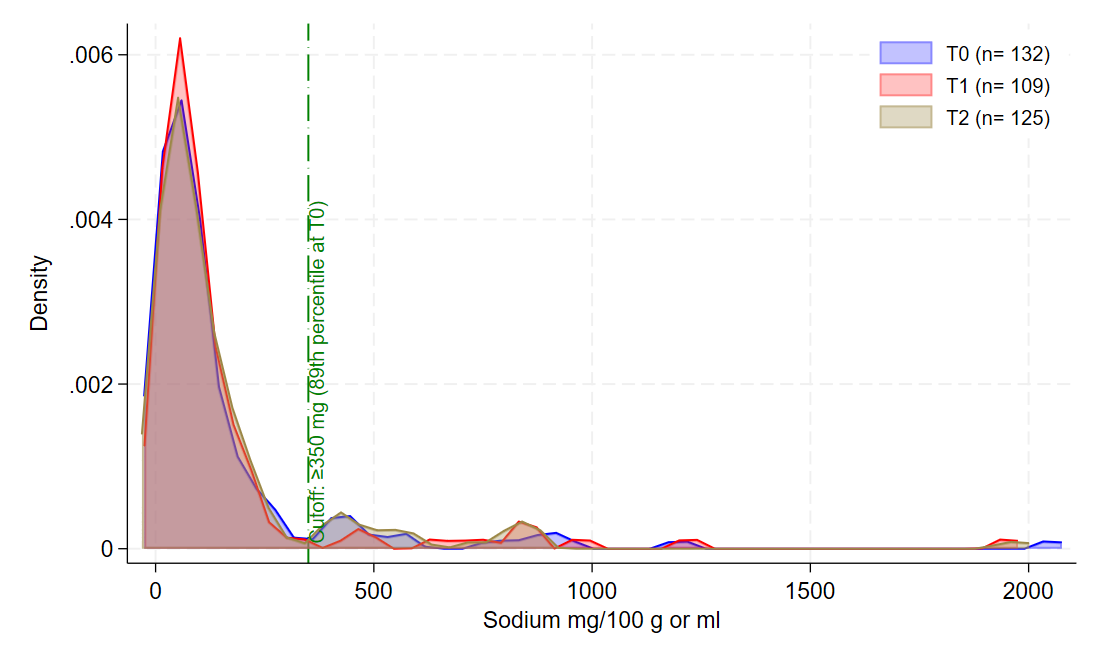 |
| **Note:** a) kcal/100 g or ml, b) Added sugar as % of total energy, c) Added sugar g/100 g or ml, d) Saturated fat as % of total energy, e) Saturated fat g/100 g or ml, f) Sodium mg/100 g or ml. All cutoffs are based on the criteria of the first stage of the front-of-package warning labels, as described in Table 1. | |

## **Table F.** Changes in calories and critical nutrients compared to T0

|  | **Kcal (per 100 g-ml)** | | **Added sugar (g/100 g-ml)** | | **Added sugar (% of energy)** | | **Saturated fat (g/100 g-ml)** | | **Saturated fat (% of energy)** | | **Sodium (mg/100 g-ml)** | |
| --- | --- | --- | --- | --- | --- | --- | --- | --- | --- | --- | --- | --- |
| **Outcome** | **T1** | **T2** | **T1** | **T2** | **T1** | **T2** | **T1** | **T2** | **T1** | **T2** | **T1** | **T2** |
| **Cereal based desserts (n=670 obs)** | | | | | | | | | | | | |
| Mean | 0.33 [.852] | -2.76 [.133] | -0.18 [.480] | -0.49 [.157] | -0.23 [.359] | -0.41 [.236] | 0.01 [.934] | **-0.41 [.003]** | 0.12 [.542] | **-0.74 [.002]** | -7.67 [.071] | **-47.02 [<.001]** |
|  | (-3.18,3.85) | (-6.36,0.85) | (-0.69,0.33) | (-1.17,0.19) | (-0.72,0.26) | (-1.09,0.27) | (-0.21,0.23) | **(-0.67,-0.14)** | (-0.26,0.50) | **(-1.22,-0.27)** | (-16.00,0.67) | **(-59.34,-34.70)** |
| 25th percentile | -2.69 [.438] | -1.65 [.644] | -0.40 [.567] | -0.40 [.633] | -0.47 [.364] | -0.54 [.394] | 0.03 [.850] | -0.19 [.306] | 0.18 [.623] | -0.46 [.398] | 1.32 [.857] | -4.97 [.638] |
|  | (-9.49,4.12) | (-8.68,5.38) | (-1.79,0.98) | (-2.06,1.25) | (-1.49,0.55) | (-1.79,0.71) | (-0.25,0.31) | (-0.56,0.17) | (-0.53,0.89) | (-1.52,0.60) | (-13.09,15.73) | (-25.70,15.77) |
| 50th percentile | 2.08 [.551] | 3.53 [.327] | -0.42 [.331] | -0.64 [.362] | -0.38 [.470] | -0.55 [.410] | 0.07 [.756] | **-0.82 [0.015]** | 0.34 [.396] | **-3.12 [<.001]** | -11.93 [.073] | **-80.19 [<.001]** |
|  | (-4.77,8.94) | (-3.54,10.60) | (-1.26,0.43) | (-2.01,0.73) | (-1.42,0.66) | (-1.87,0.76) | (-0.37,0.51) | **(-1.49,-0.16)** | (-0.45,1.14) | **(-4.55,-1.69)** | (-25.00,1.14) | **(-120.77,-39.62)** |
| 75th percentile | 4.46 [.258] | -1.85 [.639] | -0.20 [.684] | -0.55 [.452] | -0.40 [.433] | -0.22 [.705] | -0.16 [.624] | -0.29 [.482] | -0.80 [.218] | -0.63 [.384] | -19.19 [.060] | **-94.13 [<.001]** |
|  | (-3.27,12.20) | (-9.58,5.89) | (-1.15,0.76) | (-1.98,0.88) | (-1.39,0.60) | (-1.38,0.93) | (-0.82,0.49) | (-1.11,0.52) | (-2.06,0.47) | (-2.06,0.79) | (-39.18,0.79) | **(-126.27,-61.98)** |
| **Bread and other cereals (n=131 obs)** | | | | | | | | | | | | |
| Mean | **-6.90 [.031]** | -8.53 [.050] | -0.06 [.897] | -0.88 [.051] | 0.05 [.935] | -1.02 [.079] | 0.14 [.429] | 0.04 [.842] | 0.47 [.344] | 0.25 [.632] | **-53.99 [.023]** | **-123.96 [<.001]** |
|  | **(-13.14,-0.66)** | (-17.06,0.00) | (-1.00,0.88) | (-1.77,0.01) | (-1.23,1.34) | (-2.17,0.12) | (-0.21,0.49) | (-0.33,0.40) | (-0.52,1.47) | (-0.79,1.29) | **(-100.27,-7.70)** | **(-173.34,-74.58)** |
| 25th percentile | -2.75 [.565] | -8.00 [.196] | -0.25 [.736] | -0.13 [.839] | -0.72 [.561] | -0.19 [.847] | 0.78 [.081] | 0.95 [.074] | 0.00 [1.000] | 0.00 [1.000] | **-34.04 [.035]** | **-107.57 [<.001]** |
|  | (-12.24,6.73) | (-20.21,4.20) | (-1.73,1.23) | (-1.38,1.12) | (-3.17,1.73) | (-2.13,1.75) | (-0.10,1.65) | (-0.09,1.99) | (-2.53,2.53) | (-2.95,2.95) | **(-65.61,-2.46)** | **(-166.85,-48.28)** |
| 50th percentile | -10.14 [.119] | -10.14 [.171] | -0.68 [.147] | -0.89 [.093] | -0.47 [.488] | -0.75 [.274] | 0.31 [.197] | 0.14 [.582] | 1.19 [.074] | 0.35 [.681] | **-51.95 [.040]** | **-123.17 [<.001]** |
|  | (-22.95,2.67) | (-24.73,4.46) | (-1.59,0.24) | (-1.94,0.15) | (-1.83,0.88) | (-2.11,0.61) | (-0.16,0.79) | (-0.38,0.67) | (-0.12,2.50) | (-1.35,2.06) | **(-101.44,-2.45)** | **(-193.96,-52.39)** |
| 75th percentile | -0.07 [.997] | -5.56 [.760] | -0.36 [.543] | -0.70 [.227] | -0.19 [.765] | -0.46 [.419] | 0.00 [.998] | 0.06 [.867] | -0.70 [.463] | -1.28 [.175] | -40.90 [.374] | **-162.73 [0.011]** |
|  | (-31.46,31.33) | (-41.62,30.50) | (-1.52,0.80) | (-1.85,0.45) | (-1.43,1.05) | (-1.57,0.66) | (-0.65,0.65) | (-0.68,0.81) | (-2.59,1.19) | (-3.14,0.58) | (-132.01,50.22) | **(-286.38,-39.07)** |
| **Salty snacks (n=368 obs)** | | | | | | | | | | | | |
| Mean | **-11.30 [.010]** | **-8.80 [.038]** | -0.02 [.902] | -0.04 [.853] | 0.09 [.667] | -0.05 [.765] | **-0.56 [.025]** | **-1.45 [<.001]** | -0.64 [.198] | **-2.58 [<.001]** | -22.25 [.341] | -23.08 [.422] |
|  | **(-19.85,-2.76)** | **(-17.08,-0.52)** | (-0.42,0.37) | (-0.44,0.36) | (-0.33,0.51) | (-0.38,0.28) | **(-1.06,-0.07)** | **(-2.06,-0.84)** | (-1.63,0.34) | **(-3.84,-1.31)** | (-68.25,23.75) | (-79.71,33.56) |
| 25th percentile | -0.47 [.952] | -2.93 [.672] | --- | --- | --- | --- | -0.36 [.303] | -0.37 [.451] | 0.40 [.606] | -0.21 [.753] | 4.11 [.902] | **-89.94 [0.039]** |
|  | (-15.86,14.92) | (-16.54,10.69) |  |  |  |  | (-1.06,0.33) | (-1.33,0.59) | (-1.12,1.92) | (-1.53,1.11) | (-61.83,70.05) | **(-175.22,-4.67)** |
| 50th percentile | **-12.64 [.011]** | **-16.92 [0.002]** | --- | --- | --- | --- | -0.82 [.085] | **-2.77 [<.001]** | -1.32 [.142] | **-5.80 [<.001]** | -22.31 [.477] | 10.76 [.758] |
|  | **(-22.37,-2.90)** | **(-27.64,-6.21)** |  |  |  |  | (-1.76,0.11) | **(-4.05,-1.48)** | (-3.09,0.44) | **(-8.07,-3.53)** | (-84.02,39.41) | (-57.90,79.42) |
| 75th percentile | **-20.11 [.004]** | -8.67 [.274] | -0.25 [.661] | -1.31 [.070] | -0.23 [.647] | -0.63 [.246] | -0.58 [.248] | **-2.02 [0.004]** | -1.14 [.372] | **-4.52 [0.004]** | -28.51 [.555] | -38.18 [.449] |
|  | **(-33.77,-6.45)** | (-24.25,6.91) | (-1.36,0.86) | (-2.73,0.11) | (-1.23,0.77) | (-1.70,0.44) | (-1.57,0.41) | **(-3.39,-0.65)** | (-3.66,1.37) | **(-7.58,-1.45)** | (-123.54,66.52) | (-137.41,61.06) |
| **Sweetened beverages (n=489 obs)** | | | | | | | | | | | | |
| Mean | **-3.08 [<.001]** | **-2.72 [<.001]** | **-0.71 [<.001]** | **-0.68 [<.001]** | **-3.86 [.014]** | **-3.94 [.014]** | 0.00 [.106] | -0.01 [.156] | 0.16 [.272] | 0.02 [.811] | **1.08 [.026]** | 0.79 [.191] |
|  | **(-4.29,-1.88)** | **(-3.92,-1.51)** | **(-1.00,-0.43)** | **(-0.93,-0.43)** | **(-6.92,-0.80)** | **(-7.08,-0.80)** | (-0.01,0.00) | (-0.01,0.00) | (-0.13,0.45) | (-0.13,0.16) | **(0.13,2.02)** | (-0.40,1.98) |
| 25th percentile | -0.64 [.465] | **-3.02 [0.014]** | 0.00 [1.000] | 0.00 [1.000] | 0.00 [1.000] | 0.00 [1.000] | --- | --- | --- | --- | 0.63 [.475] | -0.49 [.674] |
|  | (-2.37,1.09) | **(-5.44,-0.61)** | (-0.14,0.14) | (-0.19,0.19) | (-3.64,3.64) | (-4.46,4.46) |  |  |  |  | (-1.10,2.35) | (-2.77,1.80) |
| 50th percentile | **-4.27 [.010]** | **-3.45 [0.030]** | **-0.86 [.023]** | **-0.98 [0.050]** | -2.68 [.465] | -4.12 [.298] | --- | --- | --- | --- | 0.28 [.764] | 0.57 [.559] |
|  | **(-7.51,-1.03)** | **(-6.57,-0.34)** | **(-1.60,-0.12)** | **(-1.96,0.00)** | (-9.87,4.52) | (-11.89,3.65) |  |  |  |  | (-1.53,2.08) | (-1.35,2.48) |
| 75th percentile | **-5.95 [.002]** | **-6.41 [0.001]** | **-0.64 [.045]** | -0.16 [.644] | **-6.01 [.001]** | **-9.46 [<.001]** | --- | --- | --- | --- | 0.51 [.759] | -0.40 [.855] |
|  | **(-9.75,-2.14)** | **(-10.29,-2.53)** | **(-1.27,-0.01)** | (-0.86,0.53) | **(-9.59,-2.44)** | **(-13.36,-5.55)** |  |  |  |  | (-2.76,3.78) | (-4.67,3.88) |
| **Liquid dairy (n=320 obs)** | | | | | | | | | | | | |
| Mean | **-2.76 [<.001]** | **-4.61 [<.001]** | **-0.66 [<.001]** | **-0.75 [<.001]** | **-2.68 [.003]** | **-2.35 [.004]** | 0.01 [.736] | -0.06 [.102] | 0.58 [.343] | -0.75 [.166] | -0.85 [.529] | -1.49 [.335] |
|  | **(-4.22,-1.31)** | **(-6.59,-2.63)** | **(-0.96,-0.37)** | **(-1.04,-0.45)** | **(-4.43,-0.92)** | **(-3.96,-0.75)** | (-0.06,0.08) | (-0.12,0.01) | (-0.63,1.80) | (-1.82,0.32) | (-3.51,1.81) | (-4.55,1.56) |
| 25th percentile | -5.44 [.057] | -5.86 [.133] | -0.12 [.751] | -0.43 [.356] | -1.23 [.628] | -3.25 [.240] | 0.09 [.276] | 0.03 [.642] | --- | --- | -1.16 [.672] | 4.02 [.114] |
|  | (-11.05,0.17) | (-13.52,1.80) | (-0.89,0.64) | (-1.34,0.48) | (-6.26,3.79) | (-8.68,2.19) | (-0.07,0.26) | (-0.10,0.15) |  |  | (-6.55,4.23) | (-0.98,9.02) |
| 50th percentile | 1.56 [.458] | -0.73 [.747] | -0.46 [.188] | -0.64 [.067] | **-4.94 [.005]** | **-4.12 [0.010]** | 0.06 [.222] | **-0.13 [0.046]** | --- | --- | 1.82 [.474] | -2.62 [.299] |
|  | (-2.57,5.68) | (-5.20,3.73) | (-1.15,0.23) | (-1.32,0.04) | **(-8.38,-1.49)** | **(-7.23,-1.00)** | (-0.04,0.15) | **(-0.25,0.00)** |  |  | (-3.20,6.84) | (-7.57,2.33) |
| 75th percentile | **-8.13 [.003]** | -10.43 [<.001] | **-1.07 [.016]** | **-0.85 [0.038]** | 0.00 [1.000] | 0.00 [1.000] | -0.04 [.351] | -0.07 [.142] | 0.00 [1.000] | 0.00 [1.000] | -1.39 [.655] | -2.69 [.368] |
|  | **(-13.39,-2.87)** | (-14.91,-5.95) | **(-1.93,-0.20)** | **(-1.64,-0.05)** | (-5.24,5.24) | (-4.77,4.77) | (-0.13,0.05) | (-0.16,0.02) | (-132.38,132.38) | (-153.37,153.37) | (-7.53,4.74) | (-8.56,3.19) |
| **Solid dairy (n=197 obs)** | | | | | | | | | | | | |
| Mean | **-3.76 [.013]** | **-4.73 [.005]** | **-0.28 [.032]** | **-0.39 [.036]** | -1.21 [.075] | -1.42 [.074] | **-0.36 [.002]** | **-0.47 [<.001]** | **-13.90 [<.001]** | **-8.93 [.002]** | -0.03 [.982] | 2.40 [.297] |
|  | **(-6.69,-0.82)** | **(-8.00,-1.46)** | **(-0.53,-0.02)** | **(-0.76,-0.03)** | (-2.56,0.13) | (-2.99,0.14) | **(-0.59,-0.14)** | **(-0.73,-0.21)** | **(-19.69,-8.11)** | **(-14.48,-3.38)** | (-2.98,2.92) | (-2.15,6.94) |
| 25th percentile | -5.67 [.131] | -4.69 [.179] | 0.00 [1.000] | 0.00 [1.000] | 0.00 [1.000] | 0.00 [1.000] | -0.27 [.391] | -0.62 [.213] | --- | --- | 5.91 [.080] | 3.30 [.209] |
|  | (-13.06,1.71) | (-11.56,2.17) | (-0.55,0.55) | (-0.68,0.68) | (-1.31,1.31) | (-1.57,1.57) | (-0.90,0.35) | (-1.59,0.36) |  |  | (-0.71,12.52) | (-1.88,8.48) |
| 50th percentile | -7.38 [.146] | **-13.55 [0.009]** | -0.62 [.123] | **-1.51 [0.005]** | -1.67 [.174] | **-4.45 [0.017]** | -0.05 [.848] | -0.04 [.874] | --- | --- | -2.66 [.319] | -1.44 [.632] |
|  | (-17.37,2.61) | **(-23.65,-3.45)** | (-1.41,0.17) | **(-2.55,-0.47)** | (-4.08,0.74) | **(-8.08,-0.81)** | (-0.51,0.42) | (-0.57,0.49) |  |  | (-7.91,2.60) | (-7.39,4.51) |
| 75th percentile | 10.17 [.339] | 12.88 [.325] | -0.13 [.837] | -0.71 [.252] | -1.51 [.378] | -2.41 [.143] | 0.62 [.564] | 0.30 [.672] | -23.51 [.985] | -14.85 [.984] | 0.07 [.991] | -1.98 [.769] |
|  | (-10.80,31.15) | (-12.94,38.71) | (-1.42,1.16) | (-1.93,0.51) | (-4.90,1.87) | (-5.66,0.83) | (-1.50,2.75) | (-1.09,1.68) | (-2483.58,2436.56) | (-1499.69,1469.99) | (-12.08,12.21) | (-15.28,11.32) |
| **Instant food (n=285 obs)** | | | | | | | | | | | | |
| Mean | **-9.47 [.007]** | **-10.75 [.011]** | -0.06 [.546] | -0.37 [.053] | -0.28 [.685] | -0.98 [.557] | **-0.13 [.028]** | **-0.22 [.007]** | **0.92 [.010]** | 0.25 [.570] | -18.93 [.204] | **-111.74 [.035]** |
|  | **(-16.23,-2.70)** | **(-18.97,-2.54)** | (-0.27,0.14) | (-0.74,0.00) | (-1.65,1.09) | (-4.27,2.32) | **(-0.24,-0.01)** | **(-0.37,-0.06)** | **(0.23,1.60)** | (-0.61,1.11) | (-48.31,10.44) | **(-215.41,-8.07)** |
| 25th percentile | 3.14 [.454] | -4.04 [.492] | --- | --- | --- | --- | --- | --- | --- | --- | -27.91 [.183] | -54.36 [.054] |
|  | (-5.13,11.42) | (-15.60,7.52) |  |  |  |  |  |  |  |  | (-69.11,13.28) | (-109.77,1.04) |
| 50th percentile | -2.38 [.593] | 2.10 [.750] | 0.00 [1.000] | 0.00 [1.000] | 0.00 [1.000] | 0.00 [1.000] | -0.01 [.743] | 0.00 [.916] | 0.00 [1.000] | 0.00 [1.000] | -33.49 [.077] | **-98.17 [<.001]** |
|  | (-11.16,6.40) | (-10.85,15.05) | (-0.04,0.04) | (-0.04,0.04) | (-0.47,0.47) | (-0.44,0.44) | (-0.07,0.05) | (-0.09,0.08) | (-0.60,0.60) | (-0.57,0.57) | (-70.66,3.67) | **(-146.84,-49.51)** |
| 75th percentile | -8.82 [.134] | **-17.95 [0.017]** | -0.03 [.888] | -0.26 [.647] | -1.23 [.492] | -2.74 [.457] | -0.03 [.840] | -0.23 [.250] | 1.62 [.335] | -1.15 [.610] | -84.83 [.065] | **-146.61 [0.003]** |
|  | (-20.37,2.74) | **(-32.69,-3.21)** | (-0.39,0.34) | (-1.39,0.87) | (-4.76,2.30) | (-10.00,4.51) | (-0.30,0.25) | (-0.61,0.16) | (-1.68,4.92) | (-5.62,3.31) | (-175.11,5.46) | **(-243.61,-49.62)** |
| **Candies (n=366 obs)** | | | | | | | | | | | | |
| Mean | 2.33 [.230] | 0.71 [.772] | -0.39 [.398] | -0.39 [.535] | -0.24 [.721] | -0.03 [.971] | 0.14 [.574] | -0.20 [.127] | -0.57 [.382] | **-0.86 [.048]** | 7.83 [.226] | -1.99 [.646] |
|  | (-1.49,6.14) | (-4.15,5.58) | (-1.30,0.52) | (-1.62,0.84) | (-1.55,1.07) | (-1.82,1.76) | (-0.35,0.63) | (-0.45,0.06) | (-1.84,0.71) | **(-1.71,-0.01)** | (-4.90,20.55) | (-10.56,6.57) |
| 25th percentile | -0.34 [.971] | -18.55 [.156] | 0.94 [.228] | 0.87 [.436] | 0.32 [.827] | -0.26 [.876] | 0.00 [1.000] | 0.00 [1.000] | 0.00 [1.000] | 0.00 [1.000] | 3.89 [.327] | 1.28 [.808] |
|  | (-18.88,18.20) | (-44.20,7.10) | (-0.59,2.47) | (-1.33,3.07) | (-2.59,3.23) | (-3.61,3.08) | (-0.28,0.28) | (-0.39,0.39) | (0.00,0.00) | (-0.01,0.01) | (-3.90,11.68) | (-9.12,11.69) |
| 50th percentile | 22.84 [.087] | 14.77 [.188] | 0.16 [.852] | 0.34 [.765] | -0.40 [.757] | 1.20 [.480] | -0.20 [.629] | -0.73 [.145] | 0.34 [.865] | -0.01 [.995] | 3.32 [.552] | -5.57 [.451] |
|  | (-3.32,49.00) | (-7.29,36.82) | (-1.56,1.89) | (-1.92,2.61) | (-2.98,2.17) | (-2.14,4.53) | (-1.03,0.62) | (-1.72,0.25) | (-3.62,4.30) | (-3.17,3.15) | (-7.64,14.28) | (-20.10,8.97) |
| 75th percentile | 14.22 [.254] | 2.74 [.781] | -0.79 [.672] | 0.35 [.884] | -1.69 [.375] | -2.18 [.385] | 0.50 [.527] | -0.15 [.812] | -1.67 [.185] | -2.58 [0.036] | 4.66 [.617] | 19.54 [.104] |
|  | (-10.31,38.75) | (-16.67,22.14) | (-4.48,2.89) | (-4.39,5.09) | (-5.43,2.05) | (-7.12,2.75) | (-1.05,2.05) | (-1.42,1.12) | (-4.14,0.80) | (-5.00,-0.17) | (-13.65,22.96) | (-4.05,43.13) |
| Note: P-values in square brackets. 95% confidence intervals in parentheses. P-values are rounded to three decimal places. Coefficients and confidence intervals are rounded to two decimal places. Statistically significant estimates when p-value<0.05 are bolded. Mean changes are calculated with linear models with product fixed effects. Changes at the 25th, 50th, and 75th are calculated with unconditional quantile regressions. | | | | | | | | | | | | |

## **References**

1. Secretaria de Economía. NORMA Oficial Mexicana NOM-051-SCFI/SSA1-2010, Especificaciones generales de etiquetado para alimentos y bebidas no alcohólicas preenvasados-Información comercial y sanitaria. In: Diario Oficial de la Federacion [Internet]. 2010 [cited 14 Jan 2024]. Available: https://www.dof.gob.mx/normasOficiales/4010/seeco11_C/seeco11_C.htm#:~:text=comercial%20y%20sanitaria-,NORMA%20Oficial%20Mexicana%20NOM%2D051%2DSCFI%2FSSA1%2D2010,%2D%20Secretar%C3%ADa%20de%20Econom%C3%ADa

2. Secretaria de Economía. MODIFICACIÓN de la Norma Oficial Mexicana NOM-051-SCFI/SSA1-2010, Especificaciones generales de etiquetado para alimentos y bebidas no alcohólicas preenvasados-Información comercial y sanitaria, publicada el 5 de abril de 2010. Se adicionan los incisos . In: Diario Oficial de la Federacion [Internet]. 14 Aug 2014 [cited 14 Jan 2024]. Available: https://www.dof.gob.mx/nota_detalle.php?codigo=5356328&fecha=14/08/2014#gsc.tab=0

3. Pan American Health Organization. Pan American Health Organization Nutrient Profile Model. Washington, DC; 2016. Available: https://iris.paho.org/bitstream/handle/10665.2/18621/9789275118733_eng.pdf?sequence=9&isAllowed=y

4. U.S. Department of Agriculture ARS. USDA Food and Nutrient Database for Dietary Studies 2019-2020. 2022 [cited 30 Apr 2023]. Available: Food Surveys Research Group Home Page, http://www.ars.usda.gov/nea/bhnrc/fsrg

5. Walmart México. Walmart México. 2023 [cited 31 Jul 2023]. Available: https://www.walmart.com.mx/

6. Food and Agriculture Organization of the United Nations. FAO / INFOODS Databases. Density Database Version 2.0. 2012.
